# Multigene phylogeny reveals a cryptic diversity in the genus *Dinobryon* (Chrysophyceae) with integrative description of five new species

**DOI:** 10.3389/fpls.2023.1150814

**Published:** 2023-04-18

**Authors:** Minseok Jeong, Yitong Wang, Jong Im Kim, Woongghi Shin

**Affiliations:** Department of Biology, Chungnam National University, Daejeon, Republic of Korea

**Keywords:** Chrysophyceae, *Dinobryon*, mixotroph, molecular phylogeny, stomatocyst, lorica morphology, taxonomy

## Abstract

**Introduction:**

The genus *Dinobryon* is one of the most recognizable chrysophyte genera, characterized by dendroid colonies with a biflagellate inside each cellulosic lorica. The representative forms of lorica are cylindrical, conical, vase, or funnel shaped, with undulation on the lorica wall. Traditionally, the morphological characteristics of the lorica and the colony organization have been used for the delimitation of *Dinobryon* species.

**Methods:**

To understand the taxonomy and phylogeny of colonial *Dinobryon* species, we performed molecular and morphological studies using 39 unialgal cultures and 46 single colony isolations from environmental specimens collected in Korea. We used a nuclear internal transcript spacer (ITS1-5.8S-ITS2) to find the genetic diversity of *Dinobryon* from environmental samples and a combined dataset from six gene sequences (nuclear SSU and LSU rRNA, plastid LSU rRNA, *rbc*L and *psa*A, and mitochondrial CO1 genes) for phylogenetic analysis.

**Results and discussion:**

We found 15 different lineages based on the genetic diversity of the nuclear ITS sequences. The phylogenetic tree of the colonial species based on the combined multigene dataset were divided into 18 subclades, including five new species, each with unique molecular signatures for the E23-5 helix of the V4 region in the nuclear SSU rRNA and the E11-1 helix of D7b, and the E20-1 helix of D8 regions in the nuclear LSU rRNA. Morphological studies were focused on lorica dimension and shape, and stomatocyst morphology. The *Dinobryon* species showed similarities or differences in lorica morphologies between and within species, and also differences in lorica size between culture and environmental samples. Five *Dinobryon* species formed distinctive stomatocysts, their stomatocyst morphologies, including collar structure, surface ornamentation, and cyst shape, showed unique characteristics in each species and were useful for identification. Here, we propose five new species based on morphological and molecular evidences: *D. cylindricollarium*, *D. exstoundulatum*, *D. inclinatum*, *D. similis*, and *D. spinum*.

## 1 Introduction

*Dinobryon* is a mixotrophic lineage within Chrysophyceae and its species are widely distributed in marine and freshwater environments, ranging from mainly oligotrophic alpine lakes to coastal seawaters (Siver and Chock, 1986; Halse and Heimdal, 1992; Kononen et al., 1992; Hitchman and Jones, 2000; Kamjunke et al., 2007). *Dinobryon* species have one or two yellow-greenish chloroplasts, including chlorophyll *a* and *c*, and are one of the major grazers of bacteria in oligotrophic lakes (Bird and Kalff, 1986; Domaizon et al., 2003; Kamjunke et al., 2007). Therefore, they are capable of both phototrophy and phagotrophy, with ingestion rates equal to those of heterotrophic flagellates like *Katablepharis*, *Spumella* and unidentified choanoflagellates (Bird and Kalff, 1986; Domaizon et al., 2003; Caron, 2016).

The genus was established by Ehrenberg (1834), characterized by free-living, membranous vase-shaped lorica, branching tree-like colony, and eyespot, and included two species: *Dinobryon sociale* and *D. sertularia*. A few years later, Ehrenberg (1838) suggested the additional dinobryonid genus *Epipyxis*, with its species having an epiphytic life and lacking a stigma. Stein (1878) added one new species, *D. stipitatum*, with a long champagne flute-like lorica, and also presented the first cyst morphology of *D. sertularia*, which has a slightly curved collar. In past floristic studies of *Dinobryon*, Imhof (1883; 1887; 1890) reported six new *Dinobryon* species from freshwater lakes and revised the nine described species. After Imhof’s description of the new species, the taxonomic delimitation of the genus and species levels became ambiguous and confusing due to morphological plasticity of the loricae and colonies. Lemmermann (1899) erected the new genus *Dinobryopsis*, characterized by its free-living lifestyle and solitary loricated cell. However, Lemmermann (1900) synonymized the genera *Dinobryopsis* and *Epipyxis* into the single genus *Dinobryon* and established three sections (*Epipyxis*, *Dinobryopsis*, and *Eudinobryon*) under the genus. Pascher (1913) followed Lemmermann’s classification system and further divided the section *Eudinobryon* into three series (*Sertularia*, *Stipitata*, and *Divergentia*). Schütt (1893) described the *Dinobryon*-like species from the Bay of Kiel, Baltic Sea, *Dinodendron balticum*, but the species was transferred into the genus *Dinobryon* (Lemmermann, 1900). Schiller (1925) described two additional species (*Dinobryon coalescens*, *D. porrectum*) from the Adriatic Sea. At that time, in addition to Lemmermann’s classification system, Schiller had divided the genus *Dinobryon* into two groups; *Dinobrya intracrescentia* and *Dinobrya extracrescentia*, based on the newly recognized characteristics of the difference in location of daughter lorica formation (inner vs. outer surface of mother lorica). Krieger (1930) followed Schiller’s classification system, and divided the group *Dinobrya intracrescentia* into five subgroups based on lorica morphology (vase, wineglass, conical, cylindrical, and funnel-shape) in his classification key.

Since the erection of the genus *Dinobryon*, 68 species have been recorded in the public database AlgaeBase (https://www.algaebase.org/), and there are about 40 colonial *Dinobryon* species including five marine *Dinobryon* species (Imhof, 1890; Lemmermann, 1899; Lemmermann, 1900; Lemmermann, 1901a; Lemmermann, 1901b; Lemmermann, 1904; Pascher, 1913; Reverdin, 1919; Schiller, 1925; Krieger, 1930; Hilliard and Asmund, 1963; Matvienko, 1965; Throndsen, 1997; Kristiansen and Preisig, 2011). In traditional taxonomic studies, colonial *Dinobryon* species were described based mainly on morphological characteristics, such as lorica shape and colony architecture. However, the morphological features are difficult to clearly distinguish in lorica morphotypes owing to the transition forms between *Dinobryon* species and the phenotypic plasticity depending on environmental conditions (Ahlstrom, 1937). Considering these characteristics, it is almost impossible to draw a sharp line of demarcation for species identification among the morphologically similar, colonial *Dinobryon* species. Therefore, it is considered that these taxonomic schemes are artificial and should be evaluated with molecular phylogeny based on multigene data.

Recently, the populations assigned to the *D. divergens* species complex have showed a high genetic diversity and were divided into two separate clusters in the phylogenetic analyses based on ITS (ITS1-5.8S-ITS2), mitochondrial cytochrome oxidase 1 (CO1) and nuclear SSU rDNA sequence data (Jost et al., 2010; Bock et al., 2014). Recently, two new species, *D. ningwuensis* and *D. taiyuanensis*, which are morphologically similar to *D. sertularia* and *D. sociale*, were established based on the nuclear SSU rDNA and ITS sequence data generated by a single-cell PCR from environmental samples without cell cultivation (Jiang et al., 2018a; Jiang et al., 2018b).

*Dinobryon* species produce an endogenously formed siliceous cyst called stomatocyst. The cyst is sometimes ornamented and sculptured on its surface and is a characteristic features of each species. Therefore, stomatocyst morphology can be used as an important diagnostic character to identify *Dinobryon* species (Brown et al., 1997). With the development of scanning electron microscopy (SEM), the ultrastructure of stomatocysts in *Dinobryon* species have received scientific attention, for example, as ecological paleoindicators in the sediment of certain lakes (Duff et al., 1995; Wilkinson et al., 2001). Since stomatocysts in *Dinobryon* species were first described by Stein (1878) and Bütschli (1878), the morphologies of stomatocysts have been reported in approximately 20 *Dinobryon* taxa; however, the stomatocyst ultrastructure of only five *Dinobryon* species have been described in detail using SEM (Sandgren, 1983; Piatek and Kowalska, 2008; Piatek et al., 2012; Piatek et al., 2020). Most of these were described from field specimens, and the cyst morphology on the basis of culture materials remains little known, except for that of *D. cylindricum* (Sandgren, 1983). Because species identification based only on lorica morphology may not be accurate, the description of the cyst morphological characteristics of one species from lake sediments and preserved environmental specimens is not particularly useful for species identification. For example, Sandgren (1983) reported that *D. cylindricum* produces spiny spherical cysts with slightly curved collar, while others suggested that the species has a smooth, spherical cyst with a long, hooked collar (Zeeb and Smol, 1993; Piatek et al., 2020). A way to resolve these discrepancies would be to identify the species by culturing each and observing the stomatocyst morphologies.

In light of these facts, to overcome the limitations of previous taxonomic studies based on the morphologies of the loricae and stomatocysts, the multigene phylogeny using field and culture materials is indispensable. To reevaluate the taxonomy of colonial *Dinobryon* species, we have established many culture strains from field samples, investigated the morphological diversity in *Dinobryon* by observing the shapes of loricae, colonies, and stomatocysts, and finally performed phylogenetic analyses based on the nuclear SSU and LSU rRNA; plastid LSU rRNA, *rbc*L and *psa*A; and mitochondrion cytochrome *c* oxidase (COI) gene sequences. In addition, to confirm the morphological plasticity between culture and environmental samples, after observing the morphology of the field specimens under a light microscope, they were placed in 10% Chelex^®^ 100 resin solution to extract the DNA and amplify the ITS region to identify the species

## 2 Materials and methods

### 2.1 Cultures

The newly established strains of colonial *Dinobryon* species were collected from Korean freshwater ponds. We performed single-cell isolation using a Pasteur capillary pipette and established them as unialgal cultures. Some strains were obtained from the National Center for Marine Algae and Microbiota culture collection at Bigelow Laboratory. All freshwater *Dinobryon* cells were grown in DY-V medium (Andersen et al., 2005) with distilled freshwater and *D. balticum* CCMP1766 was grown in DY-V medium with distilled seawater. All cultures were maintained at 10–17°C with a 14:10 h light:dark cycle and light intensity of 30 μmol photon·m^−2^·s^−1^ supplied by cool-white fluorescent tubes (OSRAM Korea Co., Ansan, Korea). Details on the collection sites and the Genbank accession numbers for each strain are listed in **Supplementary Table 1**.

### 2.2 Single colony isolation

To obtain genomic DNA from environmental samples, the 46 single colonies were isolated using the single-cell isolation method and transferred to a droplet of DNA nuclease-free water (Bioneer Co., Daejeon, Korea). After taking pictures of each colony, they were washed three times with the DNA nuclease-free water and transferred to a 0.2 mL PCR tube containing 100 μL of 10% (w/v) Chelex^®^ 100 resin solution (Bio-Rad Laboratories, Hercules, CA, USA). Chelex-stored samples were kept from 1 to 7 d in the dark at 4°C until DNA extraction.

### 2.3 DNA extraction, amplification, sequencing, and sequence alignment

The culture cells were harvested by centrifugation at 9,391 × *g* for 3 min (10,000 rpm, model 5424; Eppendorf, Hamburg, Germany). Genomic DNA extraction from culture cell pellets was performed using Exgene Cell SV (GeneAll Biotechnology Co., Seoul, Korea) following the protocol provided by the manufacturer. Chelex-stored samples were incubated at 95°C for 20 min. They were centrifuged at 18,407 × *g* (14,000 rpm) for 30 s and the supernatant liquid that held the genomic DNA was stored in a new 1.5 mL microcentrifuge tube. PCR was performed for the nuclear (nr) SSU, ITS, and LSU rRNA; plastid (pt) LSU rRNA; *rbc*L and *psa*A genes; and the mitochondrion (mt) COI gene using specific primers (**Supplementary Table 2**). PCR was performed in a total volume of 25 µL of the following: 1 µL of AccuPower^®^ PCR premix (Bioneer Co., Daejeon, Korea), 1 µL of forward primer, 1 µL of reverse primer, 2–5 µL of template DNA, and 12–20 µL of distilled water. The genes were amplified using a T100™ Thermal Cycler. The first denaturation was run at 94°C for 5 min, followed by 35 cycles of second denaturation at 94°C for 30 s, annealing at 42–52°C for 30–60 s, extension at 60–72°C for 1–2 min, and a final extension at 60–72°C for 7 min, with a final hold at 12°C. All PCR products were purified using the Labopass™ PCR Purification Kit or Labopass™ Gel Purification Kit (Cosmogenetech Co., Seoul, Korea) following the protocol provided by the manufacturer. The purified PCR products were sequenced using an ABI PRISM™ (3730xL; Perkin-Elmer Applied Biosystems, Foster City, CA, USA). Sequence alignments were performed visually using the Genetic Data Environment (2.6) program (Smith et al., 1994). The nucleotide sequences of the pt *rbc*L, *psa*A, and mt COI genes were aligned based on the translated amino acid sequences.

### 2.4 Molecular signatures

The secondary structures of the nuclear SSU and LSU rRNA genes were aligned based on secondary structures of the nr SSU and LSU rRNA gene sequences of the dictyochophycean species *Apendinella radians* (Ali et al., 2001). For species delimitation among the *Dinobryon* species, the E23-5 helix of the V4 region in nr SSU rRNA and the E11-1 helix of D7b and the E20-5 helix of the D8 regions in nr LSU rRNA were selected as molecular signatures.

### 2.5 Phylogenetic analyses

**Figure 1:**
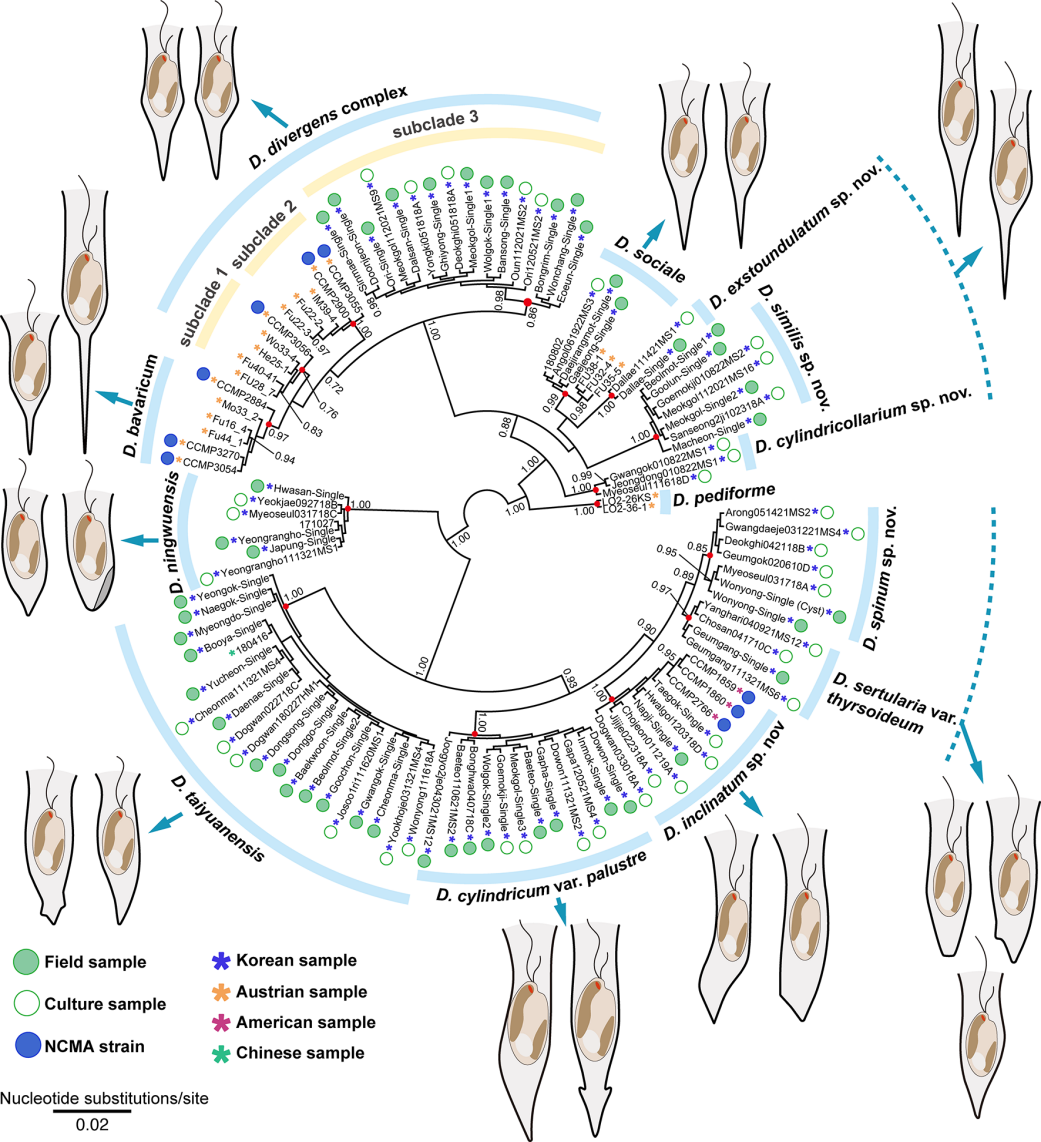
Bayesian tree of the genus *Dinobryon* based on nuclear encoded ITS sequences. Bayesian posterior probabilities (PP, right) are shown at each node. The node of each species-level lineages is indicated by red circles; the scale bar indicates the number of substitutions/site; (-) denotes values < 0.70 for PP.

**Figure 2:**
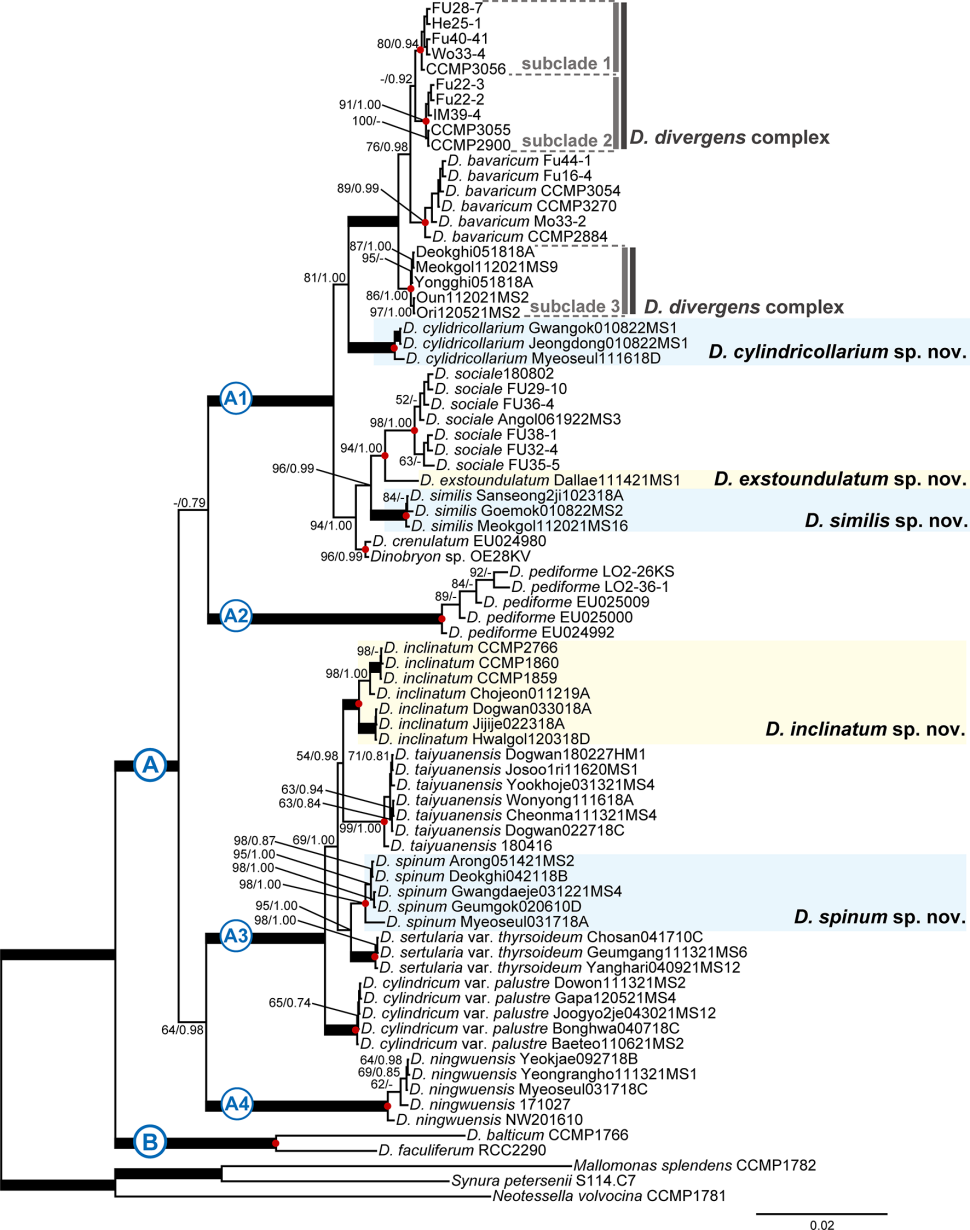
Bayesian tree of the genus *Dinobryon* based on combined nuclear SSU and LSU rDNA and plastid LSU rDNA, *rbc*L, *psa*A and mitochondrion CO1 genes data. The maximum-likelihood bootstrap values (MLBS values, left) and Bayesian posterior probabilities (PP, right) are shown at each node. The node of each species-level lineages is indicated by red circles; the scale bar indicates the number of substitutions/site; the thick line indicates full support (100% MLBS and 1.00 PP), and (-) denotes values < 50% for MLBS or 0.70 for PP.

The phylogenetic tree of Chrysophyceae was constructed by using a combined dataset of 2,429 nucleotides (nr SSU rDNA = 1,589 bp and *rbc*L gene = 840 bp) from 143 chrysophycean taxa (**Supplementary Figure 1**). The Bayesian analysis of *Dinobryon* species from culture and environmental samples was performed using 413 nucleotides of nuclear-encoded ITS sequences from 112 *Dinobryon* taxa (**Figure 1**). The phylogenetic analysis of *Dinobryon* species was performed using six genes with a combined dataset of 9,860 nucleotides (nr SSU = 1,737 bp, nr LSU = 2,500 bp, pt LSU = 2,618 bp, *rbc*L = 736 bp, *psa*A = 1,503 bp, mt CO1 = 766 bp) from 77 *Dinobryon* taxa (**Figure 2**). Three species of Synurophyceae, *Mallomonas splendens*, *Synura petersenii*, and *Neotessella volvocina* were selected as outgroup taxa. Only the conserved regions of the genes were used for phylogenetic analyses, and ambiguously aligned regions were excluded. The third codon of the *rbc*L gene was excluded from phylogenetic analysis because it is known as a fast-evolving site (Škaloud et al., 2013; Jo et al., 2016). The maximum likelihood (ML) analysis was performed using RAxML version 8.2.10 (Stamatakis, 2014) with the substitution model GTR+Γ. We generated 1,000 independent tree inferences, using the ‘#’ option of the program to identify the best tree. Maximum likelihood bootstrap (MLBS) values were calculated using 1,000 pseudo-replicates with the same substitution model. Bayesian analyses were performed using MrBayes version 3.7 (Ronquist et al., 2012), and the best-fitting model for the nucleotide dataset was selected using the Bayesian information criterion in jModelTest2 (Posada and Crandall, 1998) with the selected GTR+I+G model. Each analysis was performed using a Metropolis-coupled Markov chain Monte Carlo (MC^3^) approach, with 10,000,000 cycles for each chain. Trees were saved to a file every 1,000 cycles, and the burn-in point was identified graphically by tracking the likelihoods (Tracer version 1.7.1; http://tree.bio.ed.ac.uk/software/tracer/). The first 3,000 trees were discarded, and the remaining 7,001 trees were used to calculate the posterior probability (PP) of each clade. The trees were visualized using FigTree v.1.4.4 (http://tree.bio.ed.ac.uk/software/figtree/).

### 2.6 Light microscopy

Culture and environmental samples were observed using an Axio Imager A2 microscope (Carl Zeiss Inc., Hallbergmoos, Germany) with differential interference contrast (DIC) optics. Images were captured with an AxioCam 712 color (Carl Zeiss Inc.) photomicrographic system connected to the microscope. The numerical values of the morphological characteristics were obtained using photographic images from 25 cells (**Table 1** and **Supplementary Table 3**).

**Table 1:** Summary of the lorica morphological characteristics of new Dinobryon species.

Taxon	Strain	Habitat	Adjoining of daughter cell	Lorica morphology						
				Opening	Upper part	Lower part	Length (µm)	Opening width (µm)	Narrowest region of upper part width (µm)	Transition region width (µm)
*D. cylindricollarium*	Myeoseul111618D	Freshwater	Internal wall of mother cell	Widened	Straight to transition region or slightly narrowed to middle of upper part then widened to transition region from opening with undulation on lorica surface.	Attenuate to pointed tip from transition region	37.7–52.6	5.6–8.2	6.4–7.2	7.2–8.6
*D. exstoundulatum*	Dallae111421MS1	Freshwater	Internal wall of mother cell	Widened	Straight or slightly widened to transition region from opening with undulation on lorica surface.	Attenuate to pointed tip from transition region	33.2–45.2	6.2–8.3	–	7.4–9.4
*D. inclinatum*	Chojeon011219A	Freshwater	Internal wall of mother cell	Widened	Slightly narrowed to middle of upper part from slightly opening then widened to transition region with undulation on lorica surface.	Inclined, obliquely narrowed to pointed apex from transition region, unusual presence of protuberance.	29.1–39.1	8.1–12.4	6.7–8.3	7.4–10.6
*D. similis*	Sanseong2ji102318A	Freshwater	Internal wall of mother cell	Widened	Straight or slightly widened to transition region from opening with undulation on lorica surface.	Attenuate to pointed tip from transition region	42.0–53.8	5.8–8.2	–	6.6–8.8
*D. spinum*	Geumgok020610D	Freshwater	Internal wall of mother cell	Widened	Slightly narrowed to middle of upper from opening, then widened to transition region with undulation on lorica surface.	Obliquely narrowed to pointed or flattened apex from transition region, unusual presence of protuberance.	22.0–29.1	6.9–10.0	6.3–7.4	6.9–9.0

(-) indicates that the species lack the narrow region in upper lorica part.

### 2.7 Scanning electron microscopy

To observe stomatocyst morphology, encysted stomatocysts in cultures were collected by centrifugation for 5 min at 9,391 × *g* and boiled for 15 min in 10% HCl to remove the lorica. After washing three times with distilled water, stomatocysts were collected in a 1.5 mL microcentrifuge tube and sonicated using an ultrasonic cleaner (Sae Han Ultrasonic Co., Seoul, Korea) to completely remove the lorica from the stomatocyst. The 200–400 stomatocysts per strain were mounted on 0.45 µm nylon membrane filters (**Table 2** and **Supplementary Table 4**). The filter was mounted onto aluminum stubs using double-sided tape. The stubs were coated with platinum and viewed with a TESCAN CLARA field emission scanning electron microscope (TESCAN Ltd., Brno, Czech Republic) at 2 keV. The stomatocysts are described according to International Statospore Working Group guidelines (Cronberg and Sandgren, 1986; Wilkinson et al., 2001).

**Table 2:** Summary of newly recorded stomatocyst morphological characteristics of *Dinobryon* species.

Taxon	Strain	Stomatocyst morphology						
		Cyst body	Length of cyst body (µm)	Width of cyst body (µm)	Collar	Height of collar (µm)	Diameter of collar (µm)	Pore
*D. cylindricollarium*	Myeoseul111618D	Spherical with smooth surface	9.8–12.8	9.8–13.0	Flat planar annulus	1.5–3.3	2.3–3.1	Regular
*D. cylindricum* var. *palustre*	Bonghwa040718C	Spherical with smooth surface	11.0–13.5	11.8–13.6	Flat planar annulus with conical base and wrinkled apex	1.4–2.6	Basal 2.9–3.7 Apical 1.5–2.0	Regular
*D. inclinatum*	Chojeon011219A	Spherical to oblate with smooth surface	9.9–13.7	10.6–14.3	Hooked, flat planar annulus with conical base and wrinkled apex	Collar base 1.1–1.9 Hooked apex 1.5–2.7	Basal 1.4–3.6 Apical 1.0–2.4	Regular
*D. spinum*	Geumgok020610D	Spherical with echinate spines on cyst body surface	10.2–12.2	9.6–12.2	Flat planar annulus with conical base and wrinkled apex	1.2–2.3	Basal 2.7–3.4 Apical 1.3–2.0	Swollen pseudoannulus
*D. taiyuanensis*	Yookhoje031321MS4	Spherical with smooth surface	9.3–12.1	9.8–12.6	Slightly curved flat planar annulus	2.1–5.8	2.6–3.2	Regular

## 3 Results

### 3.1 Morphological comparison between culture and environmental samples

We performed both environmental single-colony and culture-based PCR to elucidate the genetic diversity of colonial *Dinobryon* individuals (**Figure 1**). *Dinobryon* taxa from 112 environmental and culture strains (85 strains from Korea) had high ITS sequence diversity and were divided into 15 clades with moderate-to-high support values. Single-celled lorica morphotypes on the ITS tree are representative of each clade, and the three dotted lines show the same lorica morphology with closely or distantly related lineages. For example, *Dinobryon divergens* subclade 3, including 16 Korean strains, was morphologically similar to *D. divergens* subclade 1 and 2, but the subclade was separated from *D. divergens* subclade 1 and 2. The morphological similarities in other species were also found among three clades of *D. cylindricollarium*, *D. exstoundulatum*, and *D. similis* and two clades of *D. spinum* and *D. sertularia* var. *thyrsoideum*.

We compared the lorica shape and length between culture and environmental samples of 10 species, *D. divergens* subclade 3, *D. sociale*, *D. exstoundulatum*, *D. similis*, *D. inclinatum*, *D. taiyuanensis*, *D. cylindricum* var. *palustre*, and *D. ningwuensis* (**Supplementary Figure 2** and **Supplementary Table 3**). There were no differences in lorica morphology between culture and environmental samples for most species, but there were differences in lorica length, except for in *D. ningwuensis*. The environmental samples usually had longer lorica length than did culture samples, and the biggest length difference was found in *D. inclinatum* (field sample = 41.0–49.7 μm and culture sample = 23.6–39.1 μm). In the case of *D. taiyuanensis*, the lorica length of Chinese and Korean populations showed geographic variation, with Korean specimens displaying longer lorica length (Chinese population = 20.0–28.0 μm and Korean population = 31.6–38.5 μm).

Occasionally, different morphotypes of *Dinobryon* colonies coexisted in the same habitat. For example, at Beolmot pond (36°03’02.2”N 128°54’51.5”E), both *D. exstoundulatum* and *D. taiyuanensis* occurred simultaneously, while at Dogwan pond (36°25’16.0”N 128°24’15.4”E), *D. inclinatum* and *D. taiyuanensis* were present together. At Meokgol pond (36°44’44.5”N 128°43’50.7”E), three species, namely *D. cylindricum* var. *palustre*, *D. divergens* subclade 3 and *D. similis* were found together.

### 3.2 Phylogenetic analyses

The phylogenetic analyses of Chrysophyceae based on the combined dataset of nr SSU rRNA and plastid *rbc*L gene sequences included nine orders (**Supplementary Figure 1**; Kristiansen and Škaloud, 2017), and their phylogenetic relationships were not resolved. The seven orders were well supported as a monophyletic lineage among the chrysophytes. However, Chromulinales formed a monophyletic lineage with weak supported values, and Paraphysomonadales was even a paraphyletic lineage. Ochromonadales was supported as a monophyletic clade with high support values (MLBS = 95, phylogenetic inference [PP] = 0.92) and included the genus *Dinobryon*. Within Ochromonadales, the genus *Dinobryon* formed a monophyletic clade (MLBS = 77, PP = 0.87), and had a sister relationship with the ‘*Kephyrion’* sp. CCMP3057 (MLBS = 70, PP = 0.99). The phylogenetic relationships among colonial *Dinobryon* species were not fully resolved in the phylogeny based on the combined dataset of nr SSU rDNA and pt *rbc*L gene sequences (**Supplementary Figure 1**).

Based on the combined sequence dataset of six genes, a phylogenetic tree of *Dinobryon* taxa was divided into two main clades (freshwater [A] vs. marine [B]), and then the A clade was further divided into four subclades (A1–A4) (**Figure 2**). The A1 clade consisted of eight taxa, including four new species (*Dinobryon divergens* species complex, *D. bavaricum*, *D. cylindricollarium* sp. nov., *D. sociale*, *D. exstoundulatum* sp. nov., *D. similis* sp. nov., and *D. crenulatum*). The *D. divergens* species complex clade was separated into three subclades (subclade 1, 2 and 3). *D. divergens* subclade 1 and 2 grouped together with very weakly supported values (MLBS = 48, PP = 0.92), and formed sister relationship with the clade of *D. bavaricum* (MLBS = 68, PP = 0.98). The *D. divergens* subclade 3 grouped together with the *D. divergens* subclade 1, 2 and *D. bavaricum* with strong support values (MLBS = 100, PP = 1.00). The new species *D. cylindricollarium* grouped together with the three subclades in *D. divergens* complex and *D. bavaricum* (MLBS = 71, PP = 1.00). Another new species, *D. exstoundulatum*, was a single-isolate lineage and had a sister relationship with *D. sociale* (MLBS = 89, PP = 1.00). The new species *D. similis* grouped together with *D. sociale* and *D. exstoundulatum* (MLBS = 89, PP = 0.99). The closely related species *D. crenulatum* and *Dinobryon* sp. OE28KV (MLBS = 94, PP = 0.99) were grouped with the three species *D. sociale*, *D. exstoundulatum*, and *D. similis* (MLBS = 93, PP = 1.00). The clade A2 consisted of five strains of *D. pediforme* and showed a sister relationship with the clade A1 (MLBS = 86, PP = 1.00). The clade A3 consisted of five species, including two new species: *D. inclinatum* sp. nov., *D. taiyuanensis*, *D. spinum* sp. nov., *D. sertularia* var. *thyrsoideum*, and *D. cylindricum* var. *palustre*. The two species *D. taiyuanensis* and *D. inclinatum* grouped together with weak support values (MLBS = 47, PP = 0.98). The new species *D. spinum* formed a sister relationship with *D. sertularia* var. *thyrsoideum* with high support values (MLBS = 94, PP = 1.00). The species *D. cylindricum* var. *palustre* was grouped together with four species *D. inclinatum*, *D. taiyuanensis*, *D. spinum*, and *D. sertularia* var. *thyrsoideum* (MLBS = 100, PP =1.00). The clade A4 consisted of five strains of *D. ningwuensis*, and showed a sister relationship with the clade A3 with moderate support values (MLBS = 81, PP = 1.00). The main clade B was composed of two marine species *D. balticum* and *D. faculiferum* and was separated from the freshwater *Dinobryon* species (clade A).

### 3.3 Molecular signatures

**Figure 3:**
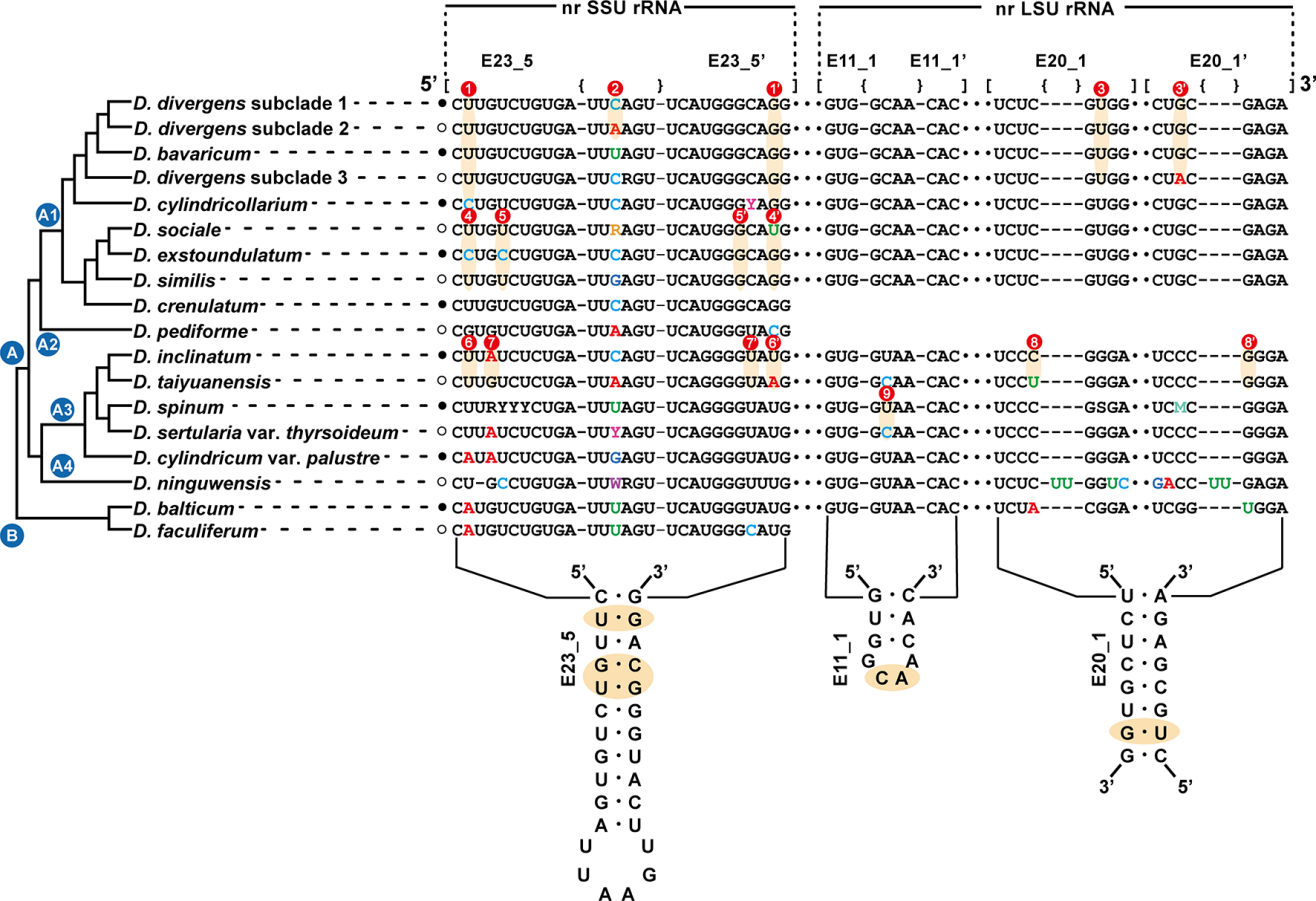
Molecular signatures of the E23-5 helix of the V4 region in the nuclear SSU rRNA, the E11-1 helix of the D7b region and the E20-1 helix of the D8 region in the nuclear LSU rRNA differentiating the *Dinobryon* species. The secondary structure was constructed based on the rRNA molecules of *D. divergens* CCMP2900. The nr LSU rRNA sequence data of *D. crenulatum* and *D. pediforme* was not available. The nomenclature of nucleotides and base pairs depends on the polarity of the DNA: increasing numbers generally indicate the 5’ to 3’ direction. [] indicates the beginning and end of the stem, with {} indicating nonbinding (loops, bulges) of the base pairs.

The sequences of the E23-5 helix of the V4 region from nr SSU rRNA, the E11-1 helix of the D7b region, and the E20-1 helix of the D8 region from nr LSU rRNA were selected as molecular signatures for the species delimitation of the genus *Dinobryon* (**Figure 3**). *Dinobryon* had unique molecular signatures in each species. The molecular signatures of new species were compared with those of closely related species. The species *D. cylindricollarium* had a different base pair, ‘C:G’, compared to the ‘U:G’ base pairs of the closely related species *D. divergens* complex and *D. bavaricum* in the E23-5 helix of nr SSU rRNA (**Figure 3**①). The *D. divergens* subclade 1 had a different base, ‘C’, compared to the ‘A’ base of *D. divergens* subclade 2 in the loop region of the E23-5 helix from nr SSU rRNA (**Figure 3**②). The *D. divergens* subclade 3 had different base pair, ‘U:A’, compared to the ‘U:G’ base pairs of the closely related species *D. divergens* subclade 1, 2 and *D. bavaricum* in the E20-1 helix from nr LSU rRNA (**Figure 3**③). Base differences between three species *D. sociale*, *D. exstoundulatum*, and *D. similis* were found in the E23-5 helix from nr SSU rRNA (**Figure 3**④, ⑤). The base pair differences between *D. inclinatum* and *D. taiyuanensis* were found in the E23-5 helix from nr SSU rRNA (**Figure 3**⑥, ⑦) and in the E20-1 helix from nr LSU rRNA (**Figure 3**⑧). The species *D. spinum* was distinguished from the closely related species *D. sertularia* var. *thyrsoideum* by their different base pairs in the E11-1 helix from nr LSU rRNA (**Figure 3**⑨).

### 3.4 Lorica

**Figure 4:**
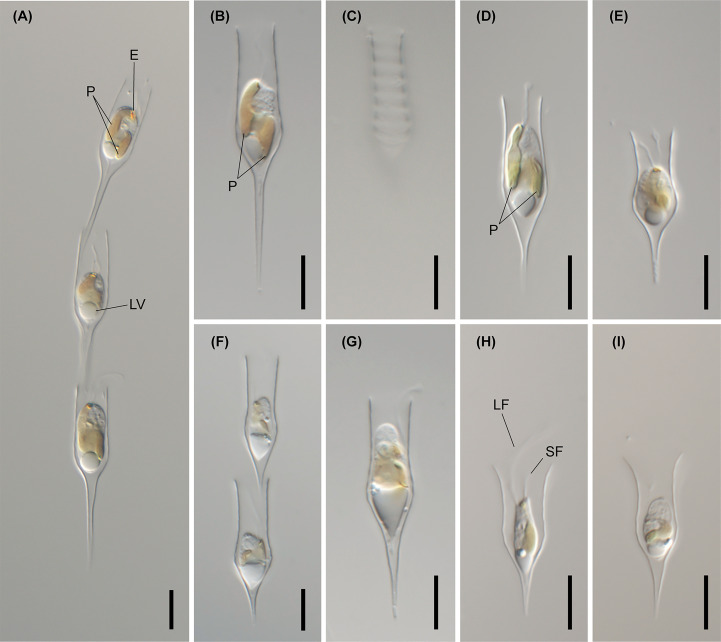
Light micrographs of *D. bavaricum*. Images showing a cell having a long (LF) and short flagella (SF), plastids (P), eyespot (E) and leucosin vesicle (LV) contained in a lorica. **(A–E)** Images of *D. bavaricum* CCMP3270. **(F, G)** Images of *D. bavaricum* CCMP3054. **(H, I)** Images of *D. bavaricum* CCMP2884. Scale bars = 10 µm.

**Figure 5:**
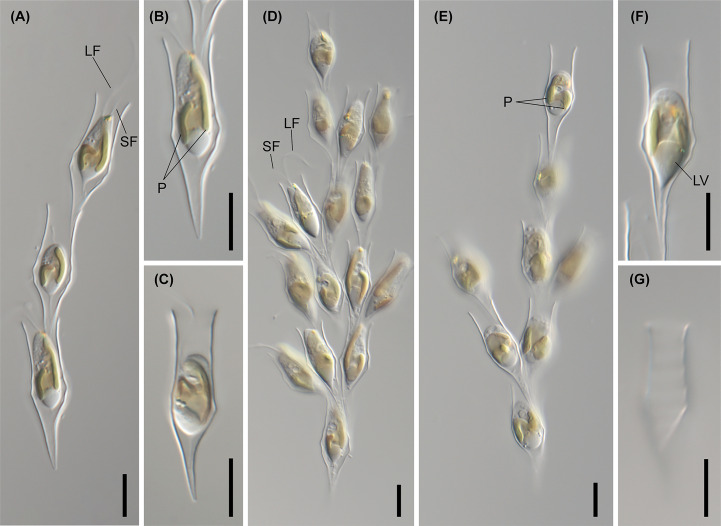
Light micrographs of *Dinobryon* species. **(A, B)** Images of *D. divergens* CCMP2900. **(C)** Image of *D. divergens* CCMP3056. **(D–G)** Images of *D. divergens* Deokghi051818A. LF, long flagellum; LV, leucosin vesicle; P, plastid; SF, short flagellum. Scale bars = 10 µm.

**Figure 6:**
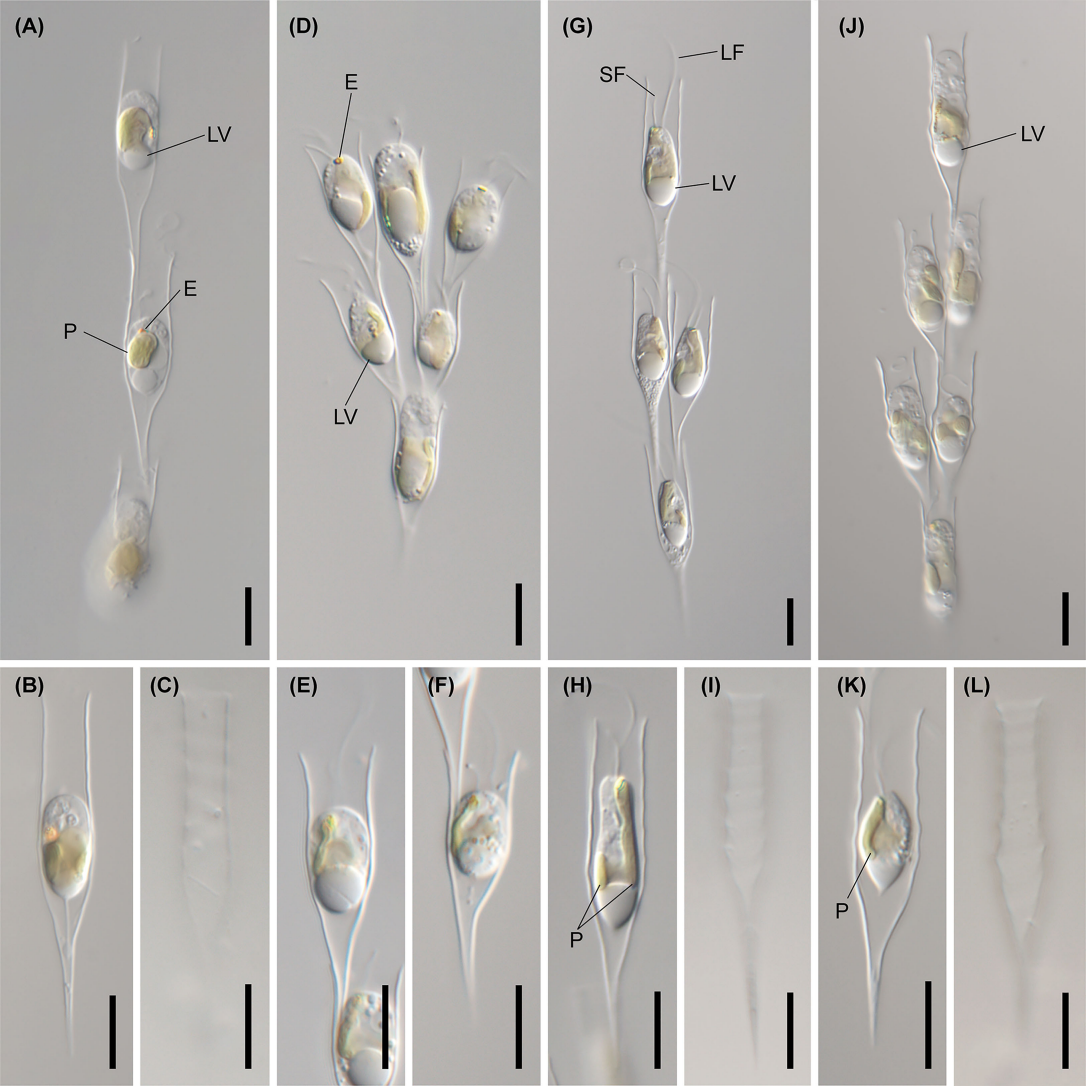
Light micrographs of *Dinobryon* species. **(A–C)** Images of *D. cylindricollarium* Myeoseul111618D. **(D–F)** Images of *D. sociale* Angol061922MS3. **(G–I)** Images of *D. similis* Sanseong2ji102318A. **(J–L)** Images of *D. exstoundulatum* Dallae111421MS1. LF, long flagellum; LV, leucosin vesicle; P, plastid; SF, short flagellum. Scale bars = 10 µm.

**Figure 7:**
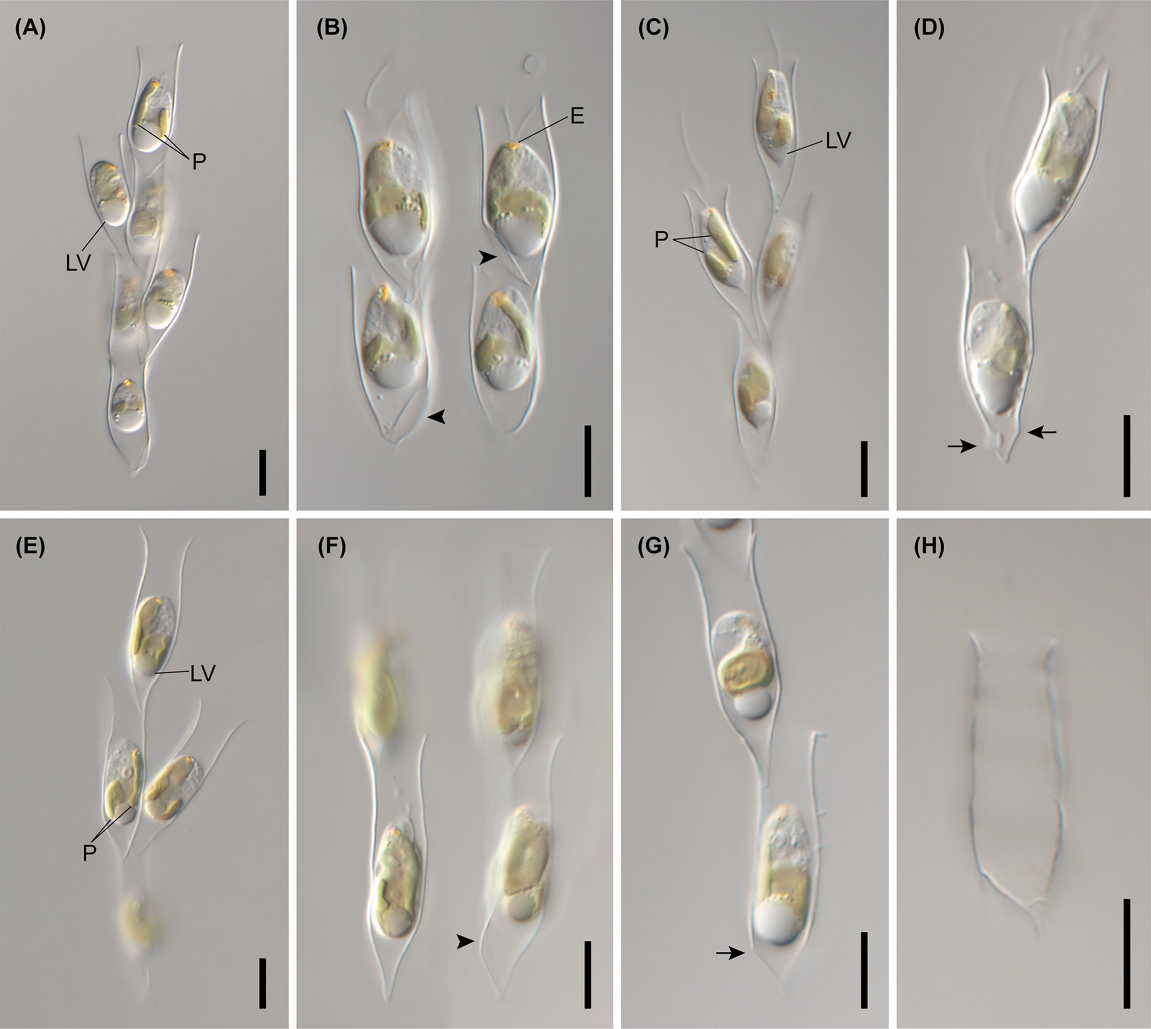
Light micrographs of *Dinobryon* species. **(A, B)** Images of *D. ningwuensis* Yeongrangho111321MS1. One side of the lower lorica cone was caved in (arrowhead). **(C, D)** Images of *D. taiyuanensis* Yookho031321MS4. Two protuberances are in both sides of the lower lorica part (arrow). **(E–H)** Images of *D. inclinatum* Chojeon011219A. Lower lorica part was inclined to one side (arrowhead). E, eyespot; LV, leucosin vesicle; P, plastid. Scale bars = 10 µm.

**Figure 8:**
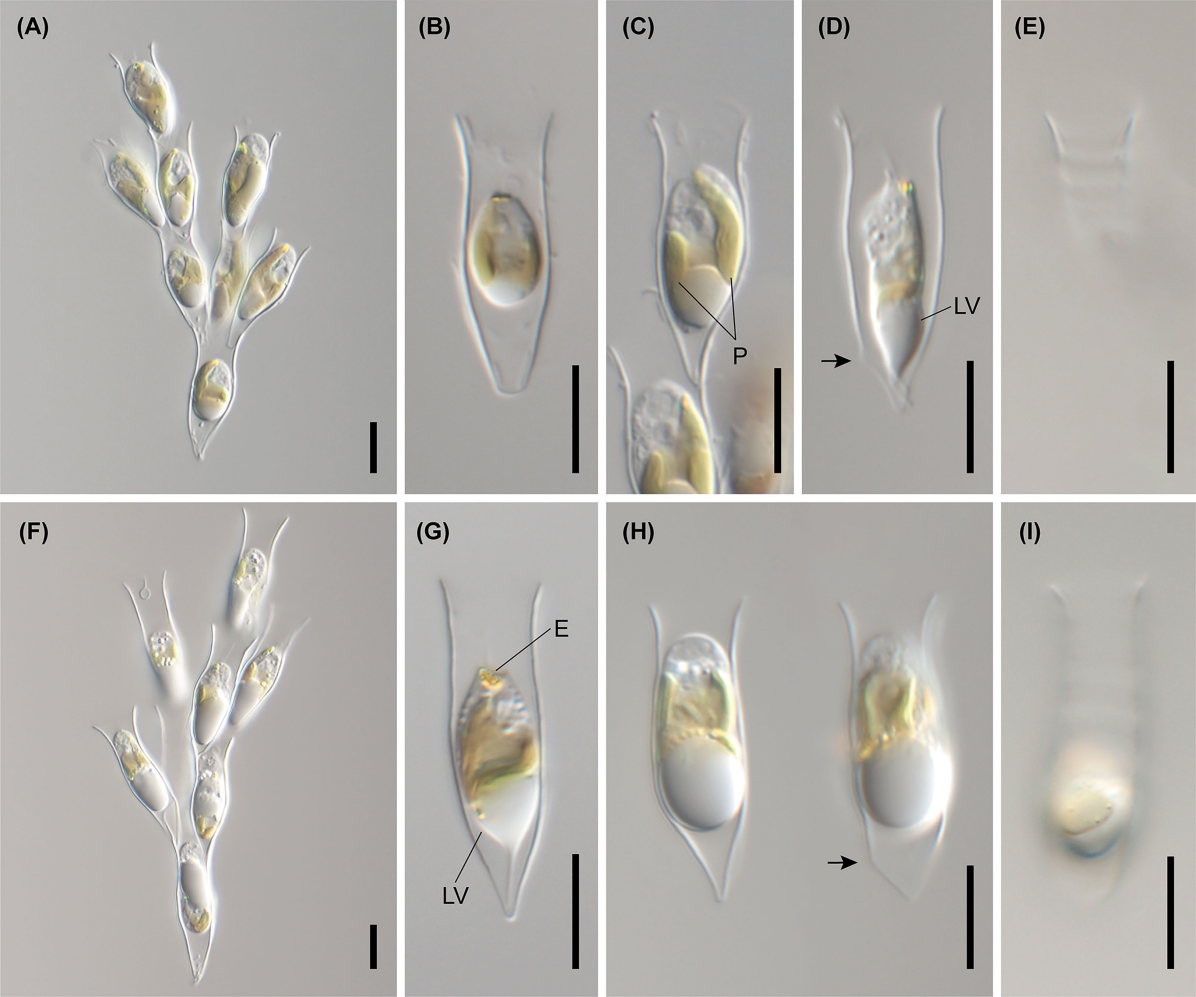
Light micrographs of *Dinobryon* species. **(A–E)** Images of *D. spinum* Geumgok020610D. **(F–I)** Images of *D. sertularia* var. *thyrsoideum* Chosan041710C. An irregular protuberance is in one side of the lower lorica part (arrow). E, eyespot; LV, leucosin vesicle; P, plastid. Scale bars = 10 µm.

**Figure 9:**
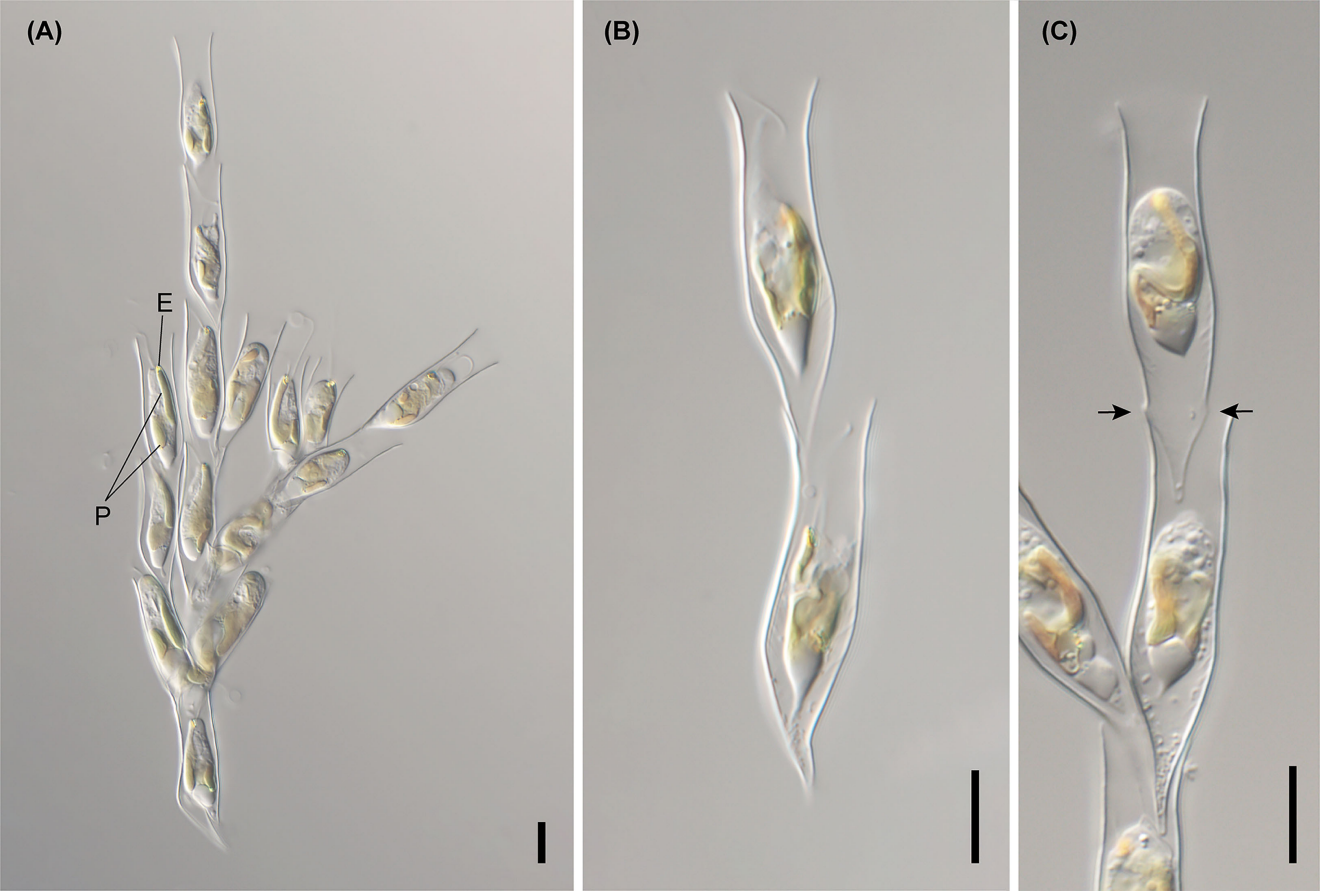
Light micrographs of *D. cylindricum* var. *palustre* Bonghwa040718C. **(A)** Colony morphology. **(B, C)** Lorica morphologies. Two protuberances are in both sides of the lower lorica part (arrow). E, eyespot; P, plastid. Scale bars = 10 µm.

The morphological characteristics of lorica among colonial *Dinobryon* species are summarized (**Table 1** and **Supplementary Table 3**). *Dinobryon* cells commonly had two unequal flagella, two plastids, one stigma, and leucosin vesicles in the posterior end of the cell. *Dinobryon* cells are enveloped by cellulosic loricae and form a dendroid colony (**Figures 4**–**9**). The lorica of *Dinobryon* was characterized by the opening part, upper part, lower part, and transition region between the upper and lower parts.

The six species (*D. divergens* complex, *D. bavaricum*, *D. cylindricollarium*, *D. sociale*, *D. exstoundulatum*, and *D. similis*) included in the subclade A1 had similar lorica shapes by having attenuated lower lorica parts (**Figures 4**–**6**). The *D. bavaricum* strains showed two different morphologies of lorica opening (**Figures 4A–I**). The two strains, *D. bavaricum* CCMP3054 and CCMP3270, had slightly widened opening (**Figures 4A–G**), whereas *D. bavaricum* CCMP2884 had a widened flaring opening (**Figures 4H, I**). Additionally, *D. bavaricum* CCMP3054 and CCMP3270 showed a striking variation in lorica length. *D. bavaricum* CCMP2884 had a shorter lorica length than did *D. bavaricum* CCMP3054 and CCMP3270 (**Figure 4**). Lorica morphology of *D. divergens* complex was extremely similar to each other (**Figure 5**). Interestingly, although *D. bavaricum* grouped together with the *D. divergens* complex (**Figures 1**, **2**), the species was morphologically differentiated from the *D. divergens* complex by its long lorica length and symmetrical lower lorica part. *D. cylindricollarium* was closely related with two *Dinobryon* species (*D. divergens* complex and *D. bavaricum*) in the phylogenetic tree (**Figure 2**); however, its lorica morphology (**Figures 6A–C**) was more similar to *D. exstoundulatum* and *D. similis* (**Figures 6G–I, J–L**) than to *D. sociale* (**Figures 6D–F**). *D. cylindricollarium* differed from the *D. divergens* complex in terms of lorica length, and *D. bavaricum* in terms of the symmetry of the lower lorica part. The species *D. exstoundulatum* formed a sister relationship with *D. sociale* in the tree, but its lorica morphology was more similar to that of *D. similis* than *D. sociale* owing to its long, cylindrical upper lorica part (**Figures 6D–L**).

The six species included in subclades A3 and A4, *D. inclinatum*, *D. taiyuanensis*, *D. spinum*, *D. sertularia* var. *thyrsoideum*, *D. cylindricum* var. *palustre*, and *D. ningwuensis*, shared a common lorica shape (**Figures 7**–**9**). They all had asymmetric or monosymmetric loricae, and the upper parts were slightly bent or not and the lower parts were obliquely curved to a pointed or obtuse end. Among these six *Dinobryon* species, concaveness in the lower lorica was a discriminative characteristic in *D. ningwuensis* (**Figures 7A, B**). Additionally, *D. inclinatum* was characterized distinctively by having a slanting region between the transition region and lower lorica (**Figure 7F**). The presence of regular protuberans at both sides in the lower lorica was found in each independent clade of *D. taiyuanensis*, as well as *D. cylindricum* var. *palustre* (**Figures 7D**, **9C**). *D. spinum* and *D. sertularia* var. *thyrsoideum* sometimes had protuberans at one side in the lower lorica (**Figures 8D, H**). All *Dinobryon* species had undulations on the cellulosic lorica surface.

### 3.5 Stomatocyst

**Figure 10:**
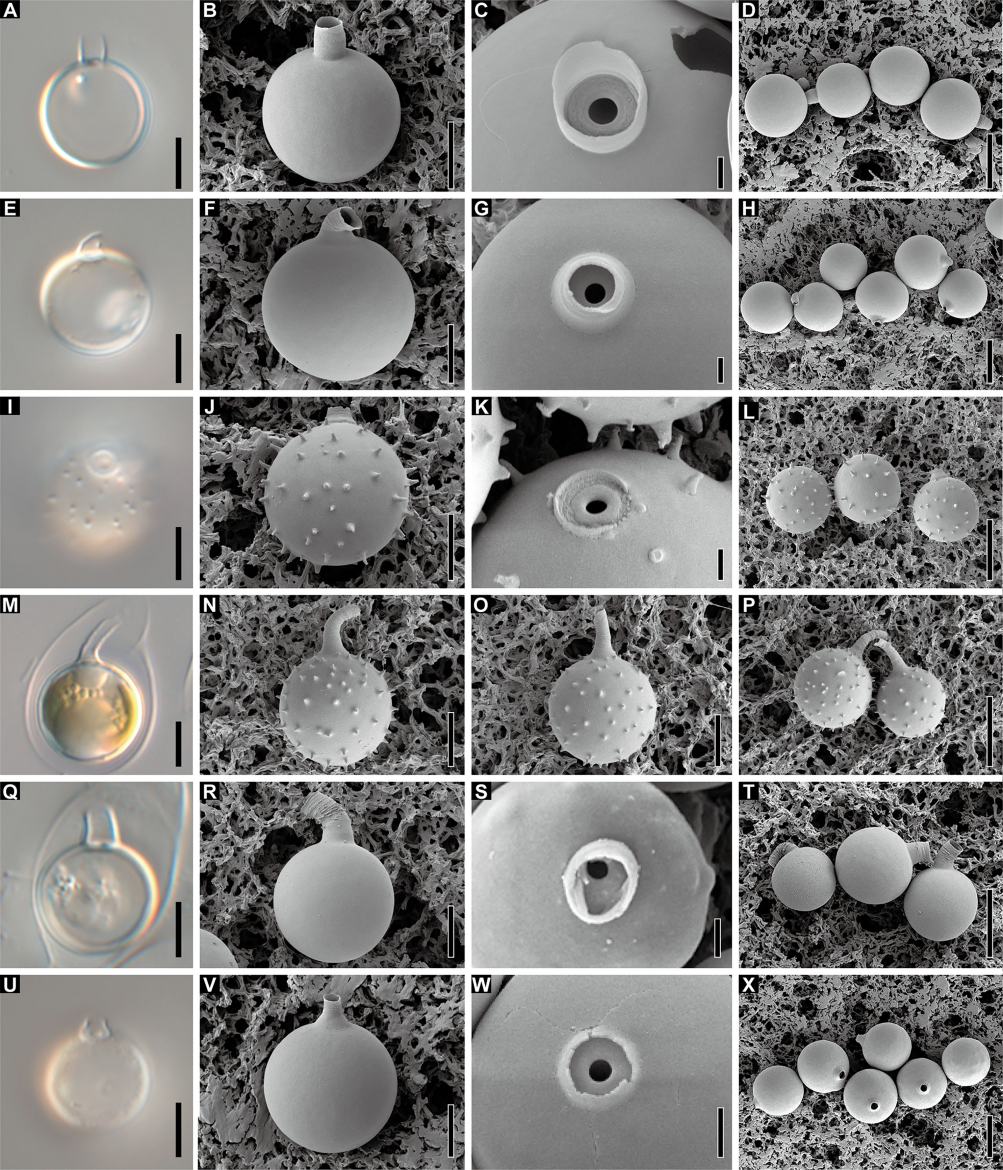
Light and scanning electron micrographs of stomatocyst. **(A–D)** Images of the spherical stomatocyst of *D. cylindricollarium* Myeoseul111618D. **(E–H)** Images of the oblate stomatocyst of *D. inclinatum* Chojeon011219A. **(I–L)** Images of the spherical stomatocyst of *D. spinum* Geumgok020610D. **(M–P)** Images of the spherical stomatocyst of *D. taiyuanensis* Yookho031321MS4. **(Q–T)** Images of the spherical stomatocyst of *D. cylindricum* var. *palustre* Bonghwa040718C. Scale bars of **A, B, E, F, I, J, M, N, Q, R** = 5 µm; **C, G, K, O, S** = 1 µm; **D, H, L, P, T** = 10 µm.

Five *Dinobryon* species, *D. cylindricollarium*, *D. inclinatum*, *D. spinum*, *D. taiyuanensis*, and *D. cylindricum* var. *palustre*, formed stomatocysts under culture conditions with distinctive collar structures (**Figure 10**). The stomatocyst shapes of the five *Dinobryon* species were spherical. *D. cylindricollarium* was characterized by its cylindrical collar (**Figures 10A, B, D**). The *D. inclinatum* had both spherical and oblate shapes (**Figures 10E–H**). The species *D. spinum* had a conspicuous ornamentation of spines on their stomatocyst surface and a swollen pseudoannulus surrounding the pore (**Figures 10I–P**). Surprisingly, the collar length of the environmental samples, collected on December 28, 2022, was very long compared to that of culture ones (**Figures 10I-P**). The stomatocysts of two previously recorded species, *D. taiyuanensis* and *D. cylindricum* var. *palustre*, were newly observed and described in this study. *D. taiyuanensis* has a spherical stomatocyst without ornamentation, characterized by a long, curved cylindrical collar with a flat planar annulus surrounding the pore (**Figures 10Q–T**). *D. cylindricum* var. *palustre* had a spherical stomatocyst without ornamentation, characterized by having a progressively narrowed collar from the cyst body and a flat planar annulus surrounding the pore (**Figures 10U–X**).

### 3.6 Description of new species

*Dinobryon cylindricollarium* Jeong, M., Kim, J. I. et Shin, W. sp. *nov*.

DESCRIPTION: Living cells are housed in loricae within an organized colony (**Figures 6A, B**). The colony is elongated and compact and composed of monosymmetric loricae (**Figure 6A**). The daughter cells attach their loricae to inside near the opening of the mother lorica (**Figure 6A**). The opening of the lorica is slightly widened (**Figures 6A, B**). The lorica slightly narrows to the middle of the upper part from the opening, then widened to the transition region (**Figures 6A, B**). The upper lorica and transition region are undulated (**Figure 6C**). The lower lorica is asymmetric when viewed from one side, but symmetric when viewed from the other. The lower part is sharply attenuated to the pointed end from the transition region, then becomes a straight or slightly diagonal long spine (**Figures 6A, B**). Lorica is 37.7–52.6 µm in length, 5.6–8.2 µm in opening width, and 7.2–8.6 µm in transition region width (n = 25).

The stomatocyst is spherical, ranges in size from 9.9 to 13.0 × 9.8 to 12.8 µm (n = 25), and has a smooth surface (**Figures 10A–D**). The collar of the stomatocyst is cylindrical, ranging in height from 1.5 to 3.3 µm and in diameter from 2.3 to 3.1 µm (n = 25), with a flat planar annulus surrounding a pore (**Figures 10A–D**).

The nr SSU and LSU rRNA molecular signature sequences are as follows:

E23-5 helix of V4 region from nr SSU rRNA: CCUGUCUGUGA;
Loop region in E23-5 helix of V4 region from nr SSU rRNA: UUCAGU;
E23-5′ helix of V4 region from nr SSU rRNA: UCAUGGGYAGG;
E11-1 helix of D7b region from nr LSU rRNA: GUG;
Loop region in E11-1 helix of D7b region from nr LSU rRNA: GCAA;
E11-1′ helix of D7b region from nr LSU rRNA: CAC;
E20-1 helix of D8 region from nr LSU rRNA: UCUCGUGG;
E20-1′ helix of D8 region from nr LSU rRNA: CUGCGAGA.

HOLOTYPE: NNIBRPR 25447, stomatocyst on SEM stub from the strain Myeoseul111618D, deposited in the Nakdonggang National Institute of Biological Resources, Sangju, Korea (NNIBR).

TYPE LOCALITY: Freshwater, pond Yeosool, Yangsa-ri, Bibong-myeon, Cheongyang-gun, Chungcheongnam-do, Korea (36°29′17.0′′N 126°45′44.9′′E), 16 Nov., 2018.

ETYMOLOGY: The specific epithet ‘*cylindricolla*’, derived from the Latin ‘*cylindri*-’ (= cylindrical) and ‘*collarium*’ (= collar), refers to the distinct, cylindrical collar of the stomatocyst.

*Dinobryon similis* Jeong, M., Kim, J. I. et Shin, W. sp. *nov*.

DESCRIPTION: Living cells are housed in loricae within an organized colony (**Figures 6G, H**). The colony is elongated and compact and composed of monosymmetric loricae (**Figure 6G**). The daughter cells attach their loricae to inside near the opening of the mother lorica (**Figure 6G**). The opening of the lorica is slightly widened or is not widened (**Figures 6G, H**). The lorica is straight or slightly widened from the opening to the transition region (**Figures 6G, H**). The upper lorica and transition region are undulated (**Figures 6G–I**). The lower lorica is asymmetric when viewed from one side, but symmetric when viewed from the other. The lower part is sharply attenuated to the pointed end from the transition region, then becomes a straight or slightly diagonal long spine (**Figures 6G, H**). Lorica is 42.0–53.8 µm in length, 6.6–8.8 µm in the transition region width, and 5.8–8.2 µm in the opening width (n = 25).

The nr SSU and LSU rRNA molecular signature sequences are as follows:

E23-5 helix of V4 region from nr SSU rRNA: CUUGUCUGUGA;
Loop region in E23-5 helix of V4 region from nr SSU rRNA: UUGAGU;
E23-5′ helix of V4 region from nr SSU rRNA: UCAUGGGCAGG;
E11-1 helix of D7b region from nr LSU rRNA: GUG;
Loop region in E11-1 helix of D7b region from nr LSU rRNA: GCAA;
E11-1′ helix of D7b region from nr LSU rRNA: CAC;
E20-1 helix of D8 region from nr LSU rRNA: UCUCGUGG;
E20-1′ helix of D8 region from nr LSU rRNA: CUGCGAGA.

HOLOTYPE: NNIBRPR 25448, a permanent microscope slide prepared from strain Sanseong2ji102318A, deposited in the Nakdonggang National Institute of Biological Resources, Sangju, Korea (NNIBR).

TYPE LOCALITY: Freshwater, pond Sanseong-2, Janggok-myeon, Hongseoeng-gun, Chungcheongnam-do, Korea (36°29′33.7′′N 126°44′19.9′′E), 23 Oct., 2018.

ETYMOLOGY: The Latin specific epithet *similis* (= similar) refers to the shape being extremely similar to *D. exstoundulatum*.

*Dinobryon exstoundulatum* Jeong, M., Kim, J. I. et Shin, W. sp. *nov*.

DESCRIPTION: Living cells are housed in loricae within an organized colony (**Figures 6J, K**). The colony is elongated and compact and composed of monosymmetric loricae (**Figure 6J**). The daughter cells attach their loricae to inside near the opening of the mother lorica (**Figure 6J**). The opening of the lorica is widened (**Figures 6J, K**). The upper lorica and transition region are broadly undulated (**Figures 6J–L**). The lorica is straight or slightly widened from the opening to the transition region (**Figures 6J, K**). The lower lorica is asymmetric when viewed from one side, but symmetric when viewed from the other. The lower part is sharply attenuated to the pointed end from the transition region, then becomes a straight or slightly diagonal long spine (**Figures 6J, K**). The lorica is 33.2–45.2 µm in length, 7.4–9.4 µm in transition region width, and 6.2–8.3 µm in opening width (n = 25).

The nr SSU and LSU rRNA molecular signature sequences are as follows:

E23-5 helix of V4 region from nr SSU rRNA: CCUGCCUGUGA;
Loop region in E23-5 helix of V4 region from nr SSU rRNA: UUCAGU;
E23-5′ helix of V4 region from nr SSU rRNA: UCAUGGGCAGG;
E11-1 helix of D7b region from nr LSU rRNA: GUG;
Loop region in E11-1 helix of D7b region from nr LSU rRNA: GCAA;
E11-1′ helix of D7b region from nr LSU rRNA: CAC;
E20-1 helix of D8 region from nr LSU rRNA: UCUCGUGG;
E20-1′ helix of D8 region from nr LSU rRNA: CUGCGAGA.

HOLOTYPE: NNIBRPR 25449, a permanent microscope slide prepared from strain Dallae111421MS1, deposited in the Nakdonggang National Institute of Biological Resources, Sangju, Korea (NNIBR).

TYPE LOCALITY: Freshwater, pond Dallae, Hawolcheon-ri, Hyeonnam-myeon, Yangyang-gun, Gangwon-do, Korea (37°55′23.7′′N 128°43′58.6′′E), 14 Nov., 2021.

ETYMOLOGY: The Latin specific epithet ‘*exsto*’ (= conspicuous) and ‘*undulatum*’ (= undulation) refers to the conspicuous undulation on the lorica wall.

*Dinobryon inclinatum* Jeong, M., Kim, J. I. et Shin, W. sp. *nov*.

DESCRIPTION: Living cells are housed in loricae within an organized colony (**Figures 7E–G**). The colony is compact or slightly divergent and composed of asymmetric lorica (**Figure 7E**). The daughter cells attach their loricae to inside near the opening of the mother lorica (**Figure 7E**). The opening of the lorica is widened (**Figures 7E–H**). The lorica is narrowed to the middle of the upper part from the opening, then widened to the transition region (**Figures 7E–H**). The upper lorica and the transition region are undulated (**Figure 7H**). The lower lorica is obliquely narrowed to the pointed end from the transition region, then becomes a cone (**Figures 7E–G**). The lower lorica is distinctively inclined to one side (**Figure 7F**). An irregular protuberance sometimes occurs on one side of the lower lorica (**Figure 7G**). The lorica is 29.1–39.1 µm in length, 6.7–8.3 µm in narrow region of the upper part width, 7.4–10.6 µm in transition region width, and 8.1–12.4 µm in opening width (n = 25).

The stomatocyst is spherical to oblate, ranges in size from 9.9 to 13.7 × 10.6 to 14.3 µm (n = 25), and has a smooth surface (**Figures 10E–H**). The collar of the stomatocyst is conical with a sharply hooked apex with flat planar annulus surrounding a pore (**Figures 10K–H**). The collar base is 1.1–1.9 µm in height (n = 18) and the hooked apex is 1.5–2.7 µm in length (n = 9). The collar is 1.4–3.6 µm in basal diameter and 1.0–2.4 µm in apical diameter (n = 18).

The nr SSU and LSU rRNA molecular signature sequences are as follows:

E23-5 helix of V4 region from nr SSU rRNA: CUUAUCUCUGA;
Loop region in E23-5 helix of V4 region from nr SSU rRNA: UUCAGU;
E23-5′ helix of V4 region from nr SSU rRNA: UCAGGGGUAUG;
E11-1 helix of D7b region from nr LSU rRNA: GUG;
Loop region in E11-1 helix of D7b region from nr LSU rRNA: GUAA;
E11-1′ helix of D7b region from nr LSU rRNA: CAC;
E20-1 helix of D8 region from nr LSU rRNA: UCCCGGGA;
E20-1′ helix of D8 region from nr LSU rRNA: UCCCGGGA.

HOLOTYPE: NNIBRPR 25450, stomatocyst on an SEM stub from strain Chojeon011219A, deposited in the Nakdonggang National Institute of Biological Resources, Sangju, Korea (NNIBR).

TYPE LOCALITY: Freshwater, pond Chojeon, Songjeong-ri, Mijo-myeon, Namhae-gun, Gyeongsangnam-do, Korea (34°43′45.1′′N 128°01′47.3′′E), 12 Jan., 2019.

ETYMOLOGY: The Latin specific epithet ‘*inclinatum*’ (= incline) refers to the inclined lower lorica.

*Dinobryon spinum* Jeong, M., Kim, J. I. et Shin, W. sp. *nov*.

DESCRIPTION: Living cells are housed in loricae within an organized colony (**Figures 8A–D**). The colony is compact or slightly divergent and composed of asymmetric lorica (**Figure 8A**). The daughter cells attach their loricae to inside near the opening of the mother lorica (**Figure 8A**). The lorica narrowed to the middle of the upper part from the opening, then widened to the transition region (**Figures 8A–D**). The upper lorica is undulated (**Figure 8E**). The lower lorica is obliquely narrowed pointed or an obtuse end from the transition region, then becomes a cone (**Figures 8A–D**). An irregular protuberance sometimes occurs on one side of the lower lorica (**Figure 8D**). The lorica is 22.0–29.1 µm in length, 6.3–7.4 µm in narrowed region of the upper part width, 6.9–9.0 µm in transition region width, and 6.9–10.0 µm in opening width (n = 25).

The stomatocyst is spherical, ranges in size from 9.6 to 12.1 × 10.2 to 12.2 µm (n=16, culture sample) or 10.4 to 11.7 × 9.8 to 11.8 µm (n=25; field sample) and has echinate spines on cyst body surface (**Figures 10I–P**). The collar of the stomatocyst is a truncated cone, ranging in length from 1.2 to 2.3 µm (n = 16; culture sample), with a swollen pseudoannulus surrounding a pore and have an elongated collar apex, ranging in length from 4.0 to 7.2 µm (n=25; field sample) (**Figures 10I–P**). The collar is 2.7–3.4 µm in basal diameter and 1.3–2.0 µm in apical diameter (n = 16; culture sample) or 2.0–3.0 µm in basal diameter and 1.0–2.7 µm in apical diameter (n=25; field sample).

The nr SSU and LSU rRNA molecular signature sequences are as follows:

E23-5 helix of V4 region from nr SSU rRNA: CUURYYYCUGA;
Loop region in E23-5 helix of V4 region from nr SSU rRNA: UUUAGU;
E23-5′ helix of V4 region from nr SSU rRNA: UCAGGGGUAUG;
E11-1 helix of D7b region from nr LSU rRNA: GUG;
Loop region in E11-1 helix of D7b region from nr LSU rRNA: GUAA;
E11-1′ helix of D7b region from nr LSU rRNA: CAC;
E20-1 helix of D8 region from nr LSU rRNA: UCCCGSGA;
E20-1′ helix of D8 region from nr LSU rRNA: UCMCGGGA.

HOLOTYPE: NNIBRPR 25451, stomatocyst on an SEM stub from strain Geumgok020610D, deposited in the Nakdonggang National Institute of Biological Resources, Sangju, Korea (NNIBR).

TYPE LOCALITY: Freshwater, pond Geumgok, Geumseong-ri, Hamla-myeon, Iksan-si, Jeollabuk-do, Korea (36°04’14.2”N 126°55’05.9”E), 12 Jan., 2019.

ETYMOLOGY: The Latin specific epithet ‘*spinum*’ (= spine) refers to the ornamentation on the surface of the stomatocyst.

## 4 Discussion

### 4.1 Single-cell PCR approach and field sample morphology

In this study, we performed a culture-independent PCR using nuclear-encoded ITS primer pairs of *Dinobryon* species to survey the integrated results of morphological and molecular characteristics from environmental samples. The investigation of molecular diversity from field samples unveiled cryptic diversity of simple and ambiguous morphologies of non-cultivated protist taxa, such as diatoms and dinoflagellates, as well as *Dinobryon* (Ki et al., 2004; Richlen and Barber, 2005; Jost et al., 2010; Lang and Kaczmarska, 2011; Bock et al., 2014; Hamilton et al., 2015). Cryptic molecular diversity in the *Dinobryon divergens* species complex has been reported using nr SSU rRNA, ITS, and mt CO1 gene sequences (Jost et al., 2010; Bock et al., 2014). Particularly, the nuclear-encoded ITS sequences were well-known, offering high-resolution for species delimitation of chrysophyte species (Auinger et al., 2008; Jost et al., 2010; Bock et al., 2017). Single-colony PCR with increased taxon sampling from Korea revealed 15 clades, more than the six previously known clades introduced by previous studies (Jost et al., 2010; Bock et al., 2014). Additionally, cryptic diversity between morphologically similar species were newly found in this study. Morphological differences between culture and environmental samples cause taxonomic difficulties to identify the species in the non-scaled chrysophycean species (Pusztai and Škaloud, 2022). In this study, there was also differences in the lorica length of *Dinobryon* species between environmental and cultured samples. For example, the environmental samples of *D. cylindricum* var. *palustre*, *D. divergens* subclade 3, *D. exstoundulatum*, *D. inclinatum*, *D. sertularia* var. *thyrsoideum*, *D. similis* and *D. spinum* had longer lorica lengths than those of the culture samples. In the case of *D. taiyuanensis*, there was difference in lorica dimension between the culture and environmental samples, and also there was geographical difference in lorica length between Chinese and Korean populations. Given these results, growth condition and geographical distribution are thought to lead to morphological variations in the lorica of *Dinobryon* species.

Morphology of stomatocyst is an important diagnostic character, but the collar length of the cyst appears to be affected by environmental factors. According to Sandgren (1983), the collar length of *D. cylindricum* stomatocysts grown at 10°C is longer than that of culture samples grown at 20°C, but the diameter of the collar apex is little variable. In this study, the collar length of *D. spinum* stomatocysts from environmental samples (frozen pond; temperature: −2.4°C to 0.6°C) was longer than that of culture samples grown in 17°C, suggesting that the collar length of the stomatocyst varies somewhat depending on environmental conditions.

### 4.2 Taxonomy of colonial *Dinobryon*

The genus *Dinobryon* have been characterized by cells that are free-living or attached to substrata, enveloped by cellulosic lorica, and form solitary or dendroid colonies (Ehrenberg, 1834; Ehrenberg, 1838; Imhof, 1887; Lemmermann, 1899; Lemmermann, 1900; Lemmermann, 1901a; Lemmermann, 1901b; Lemmermann, 1910; Pascher, 1913; Krieger, 1930). The classification system has continued to change with the addition of sections (subgenera) or series at the infrageneric level according to the views of taxonomists (Lemmermann, 1901a; Lemmermann, 1910; Pascher, 1913; Schiller, 1925; Krieger, 1930). The genus *Epipyxis*, established by Ehrenberg (1838), was transferred into one of three sections in the genus *Dinobryon* (Pascher, 1913). The section *Epipyxis* was discriminated from the sections *Dinobryopsis* and *Eudinobryon* by having silica scales on the lorica wall, but the section was resurrected eventually as the genus *Epipyxis* (Hilliard and Asmund, 1963). Our phylogenetic analyses based on nr SSU rRNA and pt *rbc*L sequence data showed a monophyly of the two genera *Epipyxis* and *Dinobryon*, and supported that *Epipyxis* and *Dinobryon* should be accepted as independent genera (**Supplementary Figure 1**).

Among three sections, colonial species have been classified into the section *Eudinobryon* and the unicellular, solitary species have been classified into the section *Dinobryopsis* (Pascher, 1913). All of the investigated *Dinobryon* species in this study were members of the section *Eudinobryon*. The section *Eudinobryon* has been further divided into three series, *Divergentia*, *Stipitata*, and *Sertularia* (Pascher, 1913). When Pascher divided the section into three series, the most important key characters were the morphotype and the symmetry of the lorica and colony architecture. In this study, we included diverse species belonging to the three series, and the resultant molecular phylogeny based on six multigene data were not congruent with the previous classification system based on lorica and colony morphology. For example, the series *Divergentia* is characterized by a monosymmetric lorica owing to a straight or oblique base part when viewed in the median, and bulky colonies, including *D. divergens*, *D. cylindricum*, and their variety species. However, *D. divergens* and *D. cylindricum* var. *palustre* were not grouped together in our phylogeny (**Figure 2**). *D. cylindricum* var. *palustre* was more closely related to *D. sertularia* var. *thyrsoideum*, which is classified to the series *Sertularia*. In addition, *D. bavaricum* and *D. sociale* of the series *Stipitata* were not supported as a monophyletic lineage. Taxa belonging in all three series were intermixed in multigene phylogeny. Therefore, the previous classification system could not be accepted as infrageneric ranks of *Dinobryon*. Given the results, the genus *Dinobryon* was divided into two groups (marine vs. freshwater) by the species’ habitats. The freshwater inhabiting *Dinobryon* species were largely divided into two groups according to the symmetry of the lorica. In the phylogenetic tree (**Figure 2**), taxa of the A1 clade have symmetric or monosymetric lorica morphotypes, and the A2, A3, and A4 clades are characterized by monosymmetric or asymmetric lorica owing to the lateral process or concave at the lower part of the lorica.

The *Dinobryon* species showed interspecific morphological similarity as well as intraspecific morphological difference. Populations with the lorica morphology of *D. divergens* have been divided into two separate clades in previous molecular phylogenies (Jost et al., 2010; Bock et al., 2014). In this study, *D. divergens* species complex had an extremely similar lorica morphology, but formed an independent clade from the *D. divergens* complex in the phylogenetic tree. The morphological similarity between the lorica of species which had para- or polyphyletic relationships was also revealed in *D. cylindricollarium*, *D. exstoundulatum*, and *D. similis*. The high morphological plasticity of lorica among intraspecies populations was found in the species *D. bavaricum*, and characterized by a lorica morphology of the upper undulated cylindrical part and lower long stem part of 80–88 µm, and the colony consisted of only a few individuals (Imhof, 1890; Krieger, 1930). However, the *D. bavaricum* CCMP2884 strain had a different lorica morphotype from the originally reported lorica shape (Imhof, 1890), with a widened flaring opening and a short lorica length. The morphological features of *D. bavaricum* CCMP2884 are more similar to those of *D. stipitatum* var. *americanum* and *D. campanulostipitum* to those of other *D. bavaricum* strains. Therefore, according to our results, the lorica morphotype-based taxonomy of the genus *Dinobryon* could be interpreted as artificial classifications, and hinders finding precise criteria for species delimitation and biodiversity.

Each new species of *Dinobryon* based on molecular phylogeny and molecular signatures was well separated from previously known species. Additionally, we compared the morphological characteristics of the lorica to distinguish new *Dinobryon* species from previous species that can be traditionally recognized based on the lorica shape. The three new species included in the A1 clade of the multigene tree, *D. cylindricollarium*, *D. exstoundulatum*, and *D. similis*, are similar to three previously known species, *D. bavaricum*, *D. bavaricum* var. *vanhöffenii*, and *D. sociale* var. *stipitatum*, in terms of having long champagne flute-like loricae. However, the three previously known species had a longer length and a more symmetric lower lorica than did our three new species. Additionally, the three new species differed from *D. bavaricum* var. *vanhöffenii* by having a divergent colony, lanceolate tip and irregular bending at the end of the lower lorica. The new species *D. inclinatum* showed the unique feature of an inclined lower cone part compared to that of other *Dinobryon* species with general cylindrical or vase-shaped lorica: *D. spinum*, *D. sertularia*, *D. sertularia* var. *thyrsoideum*, and *D. cylindricum*.

### 4.3 Stomatocyst morphology

The stomatocyst provides a more stable taxonomic characteristic to recognize certain species than the lorica morphology in *Dinobryon* species. The formation of the stomatocyst has been reported in many chrysophycean genera: *Ochromonas*, *Uroglena*, *Chromulina*, *Epipyxis*, *Mallomonas*, and *Spumella* (Scherffel, 1911; Pascher, 1914; Krieger, 1930; Lund, 1942; Hilliard and Asmund, 1963; Nygaard, 1979; Holen, 2014; Jeong et al., 2021). In the genus *Dinobryon*, the stomatocyst morphology was described in several species by line drawing and images using light and electron microscopes (Krieger, 1930; Hilliard and Asmund, 1963; Sheath et al., 1975; Smith and White, 1985; Duff et al., 1995; Piatek et al., 2012; Piatek et al., 2020). The stomatocyst morphologies from five *Dinobryon* species were newly described in this study. Especially, the three new species, *D. cylindricollarium*, *D. inclinatum*, and *D. spinum*, showed species-specific features in the ultrastructure of their stomatocysts. The stomatocyst of most *Dinobryon* species is spherical in shape without surface ornamentation and has either a cylindrical or conical collar, which is typical for *D. annulatum*, *D. divergens*, *D. pediforme*, *D. sertularia*, and *D. sociale* (Krieger, 1930; Asmund, 1955; Hilliard and Asmund, 1963; Hilliard, 1966; Sheath et al., 1975; Sandgren, 1983; Duff et al., 1995; Piatek et al., 2012; Piatek et al., 2020). The new species *D. cylindricollarium* has a longer cylindrical collar length (1.5–3.3 μm) than *D. annulatum* (slightly raised collar), *D. divergens* (1.0–1.3 μm), and *D. pediforme* (0.2–0.5 μm), and its collar width (2.3–3.1 μm) is also narrower than that of *D. sertularia* (4.2–4.8 μm). *D. sociale* has a cylindrical collar around a pore, but there is no information on the dimension of the collar yet (Krieger, 1930; Hilliard and Asmund, 1963).

The *D. inclinatum* stomatocyst has a conical, hooked collar similar to that of *D. cylindricum* (Krieger, 1930; Duff et al., 1995), *D. cylindricum* var. *palustre* (Smith and White, 1985), and *D. sertularia* (Mack, 1951). However, the *D. inclinatum* stomatocyst differed from that of *D. cylindricum*, described by Krieger (1930) and Duff et al. (1995), by having a short collar length. Furthermore, the original description of the *D. cylindricum* stomatocyst indicates that it is a spherical cyst without the collar, but has delicately dotted ornamentations on the cyst surface (Lemmermann, 1904). Although Sandgren (1983) reported that the *D. cylindricum* stomatocyst has spine-like ornamentations on cyst surface, it had a relatively longer collar than Lemmernamm’s description. Therefore, what has been described as a stomatocyst of *D. cylindricum* so far is highly likely to be a stomatocyst of another species. Smith and White (1985) reported that the stomatocyst of *D. cylindricum* var. *palustre* has a hooked collar and has a spinous process on its surface. Therefore, this species is similar in that it has a conical, hooked collar, but differs from *D. inclinatum* owing to the presence of spines on the surface of the stomatocyst. The stomatocyst morphology of *D. sertularia* is described in many different forms. Stein (1878) and Mack (1951) represented the stomatocyst with a conical, slightly curved collar, whereas Bütschli and Senn (1900) drew the cyst without the collar region. Lemmermann (1900) and Piatek et al. (2012) also presented another type of stomatocyst with a cylindrical and straight collar. These results probably show the stomatocyst diversity of some morphologically similar *Dinobryon* species, suggesting that there was cryptic diversity in *Dinobryon* species. The stomatocysts of the new species *D. inclinatum* and *D. sertularia*, described by Stein (1878), had similar morphology. The morphological differences between them are that *D. sertularia* has a curved-collared stomatocyst and vase-shaped lorica, whereas *D. inclinatum* had a hook-collared stomatocyst and cylindrical-shaped lorica.

Ornamentations on the stomatocyst surface have been reported in *D. cylindricum*, *D. cylindricum* var. *palustre*, and *D. sociale* var. *americanum* (Sandgren, 1983; Smith and White, 1985; Duff et al., 1995). The species *D. spinum* has irregular spines on the stomatocyst surface and a truncated conical collar with a flat planar annulus. The stomatocysts of *D. cylindricum* and *D. cylindricum* var. *palustre*, described by Sandgren (1983) and Smith and White (1985), respectively, also have spines on the cyst surface and slightly curved, elongated collars. Another species, *D. sociale* var. *americanum*, has irregular verrucae on the cyst surface and an obconical collar. The stomatocysts of the three species *D. cylindricollarium*, *D. inclinatum*, and *D. spinum* species differed from those of *D. crenulatum*, which has an obconical collar (Asmund, 1955), and *D. pediforme*, which has a very short and cylindrical collar (Steinecke, 1915).

### 4.4 Molecular signature

The molecular signatures of the E23-5 helix in the V4 region of nr SSU rRNA and E11-1 helix in the D7b region and E20-1 helix in the D8 region of nr LSU rRNA were designed as taxonomic key characteristics to identify the *Dinobryon* species. Since identifying microalgal taxa using ambiguous and simple morphological characteristics is very difficult, molecular signatures have been efficiently used for the delimitation of species (Marin et al., 2003; Škaloudova and Škaloud, 2013; Kim et al., 2013a; Kim et al., 2013b; Kim et al., 2017; Jeong et al., 2021). In solely using morphological features to identify the species of *Dinobryon*, the lorica morphology was insufficient and the ultrastructure of the stomatocyst are not described in many species. The selected sequences, as molecular signature regions, are well conserved within *Dinobryon* intraspecies, but are variable enough among interspecies. The V4 region of nr SSU rRNA is a well-known genetic marker for understanding microalgal diversity (Coleman, 2009; Zimmermann et al., 2011; Bock et al., 2017; Tragin et al., 2017). However, sequence dissimilarity between *Dinobryon* species in the V4 region of 18S rDNA may be high for some species but not others; for example, the nucleotide sequence of this region was 100% identical between *D. inclinatum* and *D. sertularia* var. *thyrsoideum* Yanghari040921MS12. Therefore, we used a combination of the three domains from nuclear SSU and LSU rDNA sequences to evaluate the delimitation of *Dinobryon* species. Additionally, the molecular signatures are congruent with the phylogenetic relationships as a monophyletic lineage and can help to identify *Dinobryon* species.

## 5 Conclusions

In this study, we performed a systematic study of *Dinobryon* species based on molecular and morphological evidence using both culture and culture-independent materials. Morphological characteristics, including the lorica and stomatocyst, had a few limitations in identifying the species and revealing cryptic diversity. In particular, the lorica morphologies are very similar among closely or distantly related species, making it difficult to differentiate them, while the stomatocyst morphologies showed delicate differences among related species. The phylogenetic tree based on the multigene data showed cryptic diversity as well as phylogenetic relationships among *Dinobryon* species. In addition, classification systems such as series and section, which have been used in previous classification systems, were not congruent with our molecular phylogenetic tree. Based on this evidence, we have improved the taxonomy and the knowledge of species diversity in the genus *Dinobryon*, and describe five new species.

## Data availability statement

The datasets presented in this study can be found in online repositories. The names of the repository/repositories and accession number(s) can be found below: Genbank accession numbers: OQ453601-OQ453650, OQ453391-OQ453484, OQ453222-OQ453370, OQ453551-OQ453600, OQ466159-OQ466208, OQ466209-OQ466258, OQ466259-OQ466307.

## Author contributions

MJ, YW, JIK, and WS conceived and designed the experiments. MJ, YW, and JIK performed LM image recording, DNA extraction, PCR amplification and phylogenetic analysis. MJ and WS performed the SEM-based image recordings. MJ, YW, JIK, and WS interpreted the data and wrote the manuscript. All authors contributed to the article and approved the submitted version.
