# Supplementary figures and images for: Multigene phylogeny reveals a cryptic diversity in the genus Dinobryon (Chrysophyceae) with integrative description of five new species
Source: Front Plant Sci. 2023 Apr 18;14:1150814. doi: 10.3389/fpls.2023.1150814 (PMC10151809; doi:10.3389/fpls.2023.1150814)

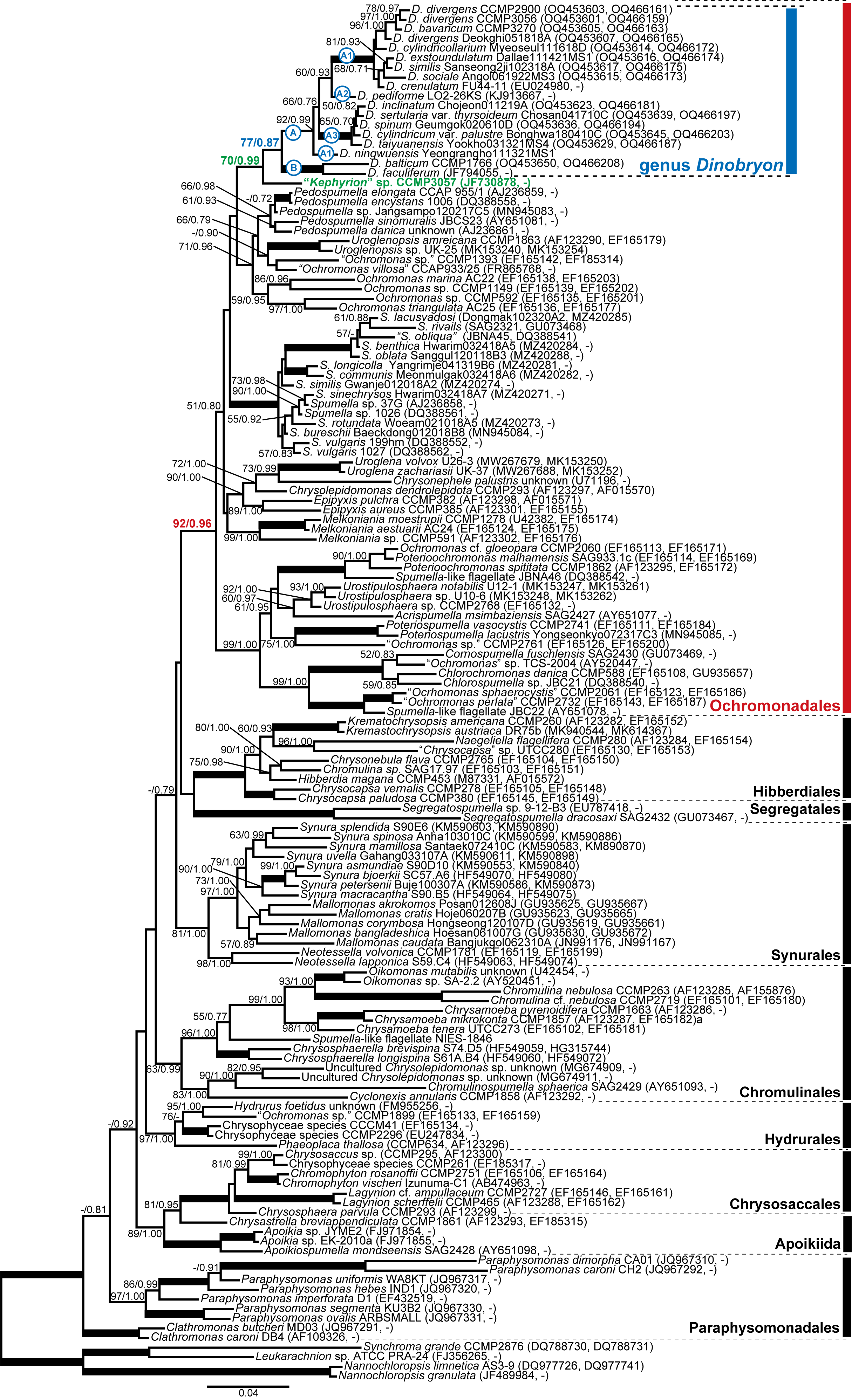

Supplement: Supplementary file 1 [file Image_1.tif]

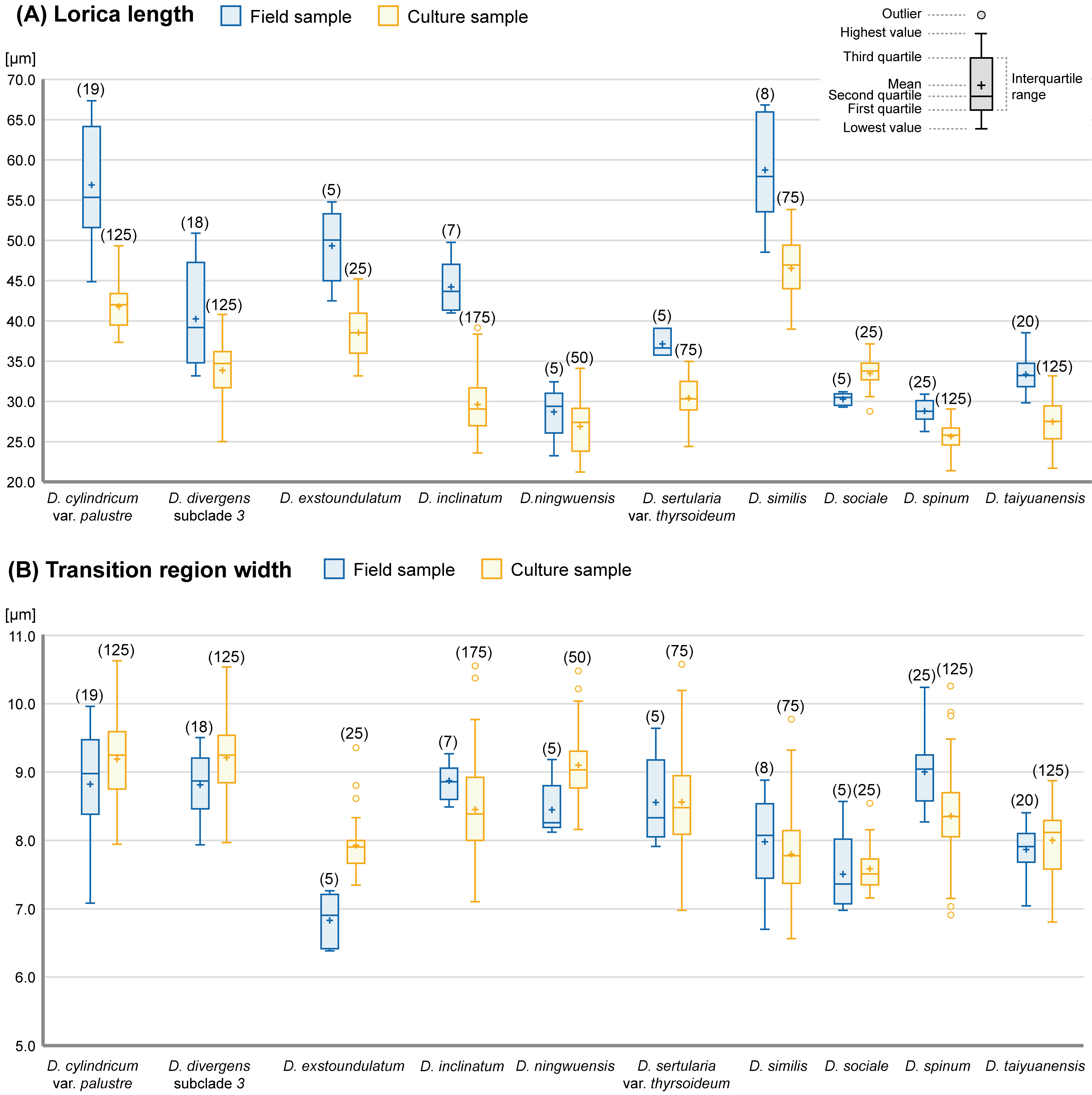

Supplement: Supplementary file 2 [file Image_2.tif]
