# Supplementary material for: Multigene phylogeny reveals a cryptic diversity in the genus Dinobryon (Chrysophyceae) with integrative description of five new species
Source: Front Plant Sci. 2023 Apr 18;14:1150814. doi: 10.3389/fpls.2023.1150814 (PMC10151809; doi:10.3389/fpls.2023.1150814)
Supplement: Supplementary file 3 [file Table_1.docx]

**Supplementary table 1.** Strain information of *Dinobryon* and three outgroup taxa used in this study and the GenBank accession numbers for their nuclear SSU, LSU rRNA and ITS gene, plastid LSU rRNA, *rbc*L and *psa*A gene sequences.

| **Taxon** | **Strain** | **Collection site (GPS coordinates )** |  | **Genbank accession number** | | | | | | |  |
| --- | --- | --- | --- | --- | --- | --- | --- | --- | --- | --- | --- |
|  |  |  | **Nuclear SSU** | | **Nuclear ITS** | **Nuclear**  **LSU** | **Plastid**  **LSU** | **Plastid**  ***rbc*L** | **Plastid**  ***psa*A** | **Mitochondria**  **CO1** |  |
| ***Dinobryon*** |  |  |  | |  |  |  |  |  |  |  |
| *D. balticum* (Schütt) Lemmermann | CCMP1766 | Southern Kattegat, Denmark (56°N 11°W) | OQ453650 | | - | OQ453370 | OQ453600 | OQ466208 | OQ466258 | OQ466307 |  |
| *D. bavaricum* Imhof | CCMP2884 | Lake Ödensee, Styria region, Austria (47.351°N 13.8167°W) | OQ453606 | | OQ453393 | OQ453327 | OQ453556 | OQ466164 | OQ466214 | OQ466264 |  |
| *D. bavaricum* Imhof | CCMP3054 | Lake Fuschlsee, Salzkammergut, Austria (47.81°N 13.256°W) | OQ453604 | | OQ453391 | OQ453325 | OQ453554 | OQ466162 | OQ466212 | OQ466262 |  |
| *D. bavaricum* Imhof | CCMP3270 | Lake Fuschlsee, Salzkammergut, Austria (47.81°N 13.256°W) | OQ453605 | | OQ453392 | OQ453326 | OQ453555 | OQ466163 | OQ466213 | OQ466263 |  |
| *D. bavaricum* Imhof | Fu16_4 | Salzkammergut area of Austria | FN662757 | | | - | - | - | - | FN664006 |  |
| *D. bavaricum* Imhof | Fu44_1 | Lake Fuschlsee, Salzkammergut, Austria | FN662760 | | | - | - | - | - | KJ579422 |  |
| *D. bavaricum* Imhof | Mo33_2 | Lake Fuschlsee, Salzkammergut, Austria | KJ579315 | | | - | - | - | - | KJ579400 |  |
| *D. divergens* Imhof | Bansong-Single | Wolsong-ri, Seo-myeon, Chuncheon-si, Gangwon-do, Korea (37°55'41.2"N 127°41'46.7"E) | - | | | OQ453398 | - | - | - | - | - |
| *D. divergens* Imhof | Bongrim-Single | Jacheon-ri, Hwabuk-myeon, Yeongcheon-si, Gyeongsangbuk-do, Korea (36°07'27.1"N 128°53'56.4"E) | - | | OQ453412 | - | - | - | - | - |  |
| *D. divergens* Imhof | CCMP2900 | Lake Wallersee, Salzburg lake district, Austria (47.9078°N 13.1685°W) | OQ453603 | | OQ453396 | OQ453324 | OQ453553 | OQ466161 | OQ466211 | OQ466261 |  |
| *D. divergens* Imhof | CCMP3055 | Lake Fuschlsee, Salzkammergut, Austria (47.81°N 13.256°W) | OQ453602 | | OQ453395 | OQ453323 | OQ453552 | OQ466160 | OQ466210 | OQ466260 |  |
| *D. divergens* Imhof | CCMP3056 | Lake Fuschlsee, Salzkammergut, Austria (47.81°N 13.256°W) | OQ453601 | | OQ453394 | OQ453322 | OQ453551 | OQ466159 | OQ466209 | OQ466259 |  |
| *D. divergens* Imhof | Dalsan-Single | Jacheon-ri, Hwabuk-myeon, Yeongcheon-si, Gyeongsangbuk-do, Korea (36°06'42.1"N 128°56'08.2"E) | - | | OQ453400 | - | - | - | - | - |  |
| *D. divergens* Imhof | Deokghi051818A | Deokghi-dong. Iksan-si, Jeollabuk-do, Korea (35°58'42.6"N 127°02'07.2"E) | OQ453607 | | OQ453397 | OQ453328 | OQ453557 | OQ466165 | OQ466215 | OQ466265 |  |
| *D. divergens* Imhof | Doonjeon-Single | Moolgab-ri, Ganghyeon-myeon, Yangyang-gun, Gangwon-do, Korea (38°07'22.4"N 128°32'25.4"E) | - | | OQ453409 | - | - | - | - | - |  |
| *D. divergens* Imhof | Eoeun-Single | Eoeun-ri, Geumsu-myeon, Seongju-gun, Gyeongsangbuk-do, Korea (35°56'12.1"N 128°09'54.7"E) | - | | OQ453410 | - | - | - | - | - |  |
| *D. divergens* Imhof | Fu22_2 | Salzkammergut area of Austria | FN662720 | | | - | - | - | - | FN664008 |  |
| *D. divergens* Imhof | Fu22_3 | Lake Fuschlsee, Salzkammergut, Austria | FN662721  - | | | - | - | - |  | FN664009 |  |
| *D. divergens* Imhof | Fu28_7 | Lake Fuschlsee, Salzkammergut, Austria | KJ579324 | | | - | - | - | - | KJ579367 |  |
| *D. divergens* Imhof | Fu40_41 | Salzkammergut area of Austria | FN662746 | | | - | - | - | - | FN663991 |  |
| *D. divergens* Imhof | Ghiyong-Single | Donggok-dong, Gwangsan-gu, Gwangju-si, Korea (35°05'57.8"N 126°45'50.8"E) | - | | OQ453399 | - | - | - | - | - |  |
| *D. divergens* Imhof | HE25_1 | Lake Fuschlsee, Salzkammergut, Austria | KJ579332 | | | - | - | - | - | KJ579386 |  |
| *D. divergens* Imhof | IM39_4 | Lake Fuschlsee, Salzkammergut, Austria | KJ579334 | | | - | - | - | - | KJ579398 |  |
| *D. divergens* Imhof | Meokgol112021MS9 | Oun-ri, Pyeongeun-myeon, Yeongju-si, Gyeongsangbuk-do, Korea (36°44'44.5"N 128°43'50.7"E) | OQ453608 | | OQ453403 | OQ453329 | OQ453558 | OQ466166 | OQ466216 | OQ466266 |  |
| *D. divergens* Imhof | Meokgol-Single1 | Oun-ri, Pyeongeun-myeon, Yeongju-si, Gyeongsangbuk-do, Korea (36°44'44.5"N 128°43'50.7"E) | - | | OQ453402 | - | - | - | - | - |  |
| *D. divergens* Imhof | Ori120521MS2 | Odong-ri, Hwabuk-myeon, Yeongcheon-si, Gyeongsangbuk-do, Korea (36°05'48.9"N 128°54'55.8"E) | OQ453611 | | OQ453404 | OQ453332 | OQ453561 | OQ466169 | OQ466219 | OQ466269 |  |
| *D. divergens* Imhof | Ori-Single | Odong-ri, Hwabuk-myeon, Yeongcheon-si, Gyeongsangbuk-do, Korea (36°05'48.9"N 128°54'55.8"E) | - | | OQ453405 | - | - | - | - | - |  |
| *D. divergens* Imhof | Oun112021MS2 | Oun-ri, Pyeongeun-myeon, Yeongju-si, Gyeongsangbuk-do, Korea (36°43'12.0"N 128°43'29.8"E) | OQ453610 | | OQ453401 | OQ453331 | OQ453560 | OQ466168 | OQ466218 | OQ466268 |  |
| *D. divergens* Imhof | Sinmae-Single | Seosang-ri, Seo-myeon, Chuncheon-si, Gangwon-do, Korea (37°55'58.4"N 127°39'03.2"E) | - | | OQ453408 | - | - | - | - | - |  |
| *D. divergens* Imhof | Wolgok-Single1 | Wolgok-ri, Chojeon-myeon, Seongju-gun, Gyeongsangbuk-do, Korea (35°59'55.0"N 128°14'36.2"E) | - | | OQ453407 | - | - | - | - | - |  |
| *D. divergens* Imhof | Wonchang-Single | Wonchang-ri, Dongsan-myeon, Chuncheon-si, Gangwon-do, Korea (37°48'03.6"N 127°47'20.8"E) | - | | OQ453411 | - | - | - | - | - |  |
| *D. divergens* Imhof | Wo33_4 | Lake Fuschlsee, Salzkammergut, Austria | KJ579346 | | | - | - | - | - | KJ579412 |  |
| *D. divergens* Imhof | Yongghi051818A | Yongghi-ri, Simwon-myeon, Gochang-gun, Jeollabuk-do, Korea (35°32'00.1"N 126°35'04.5"E) | OQ453609 | | OQ453406 | OQ453330 | OQ453559 | OQ466167 | OQ466217 | OQ466267 |  |
| *D. crenulatum* West & West | FU44-11 | unknown | EU024980 | | - | - | - | - | - | - |  |
| *D. cylindricollarium* Jeong, Kim & Shin | Myeoseul111618D | Yangsa-ri, Bibong-myeon, Cheongyang-gun, Chuncheongnam-do, Korea (36°29'17.0"N 126°45'44.9"E) | OQ453614 | | OQ453427 | OQ453335 | OQ453564 | OQ466172 | OQ466222 | OQ466272 |  |
| *D. cylindricollarium* Jeong, Kim & Shin | Gwangok010822MS1 | Anin-ri, Cheongdo-eup, Cheongdo-gun, Gyeongsangbuk-do, Korea (35°40'40.4"N 128°45'39.9"E) | OQ453612 | | OQ453425 | OQ453333 | OQ453562 | OQ466170 | OQ466220 | OQ466270 |  |
| *D. cylindricollarium* Jeong, Kim & Shin | Jeongdong010822MS1 | Daehang-ri, Boobook-myeon, Milyang-si, Gyeongsangnam-do, Korea (35°31'33.9"N 128°41'13.5"E) | OQ453613 | | OQ453426 | OQ453334 | OQ453563 | OQ466171 | OQ466221 | OQ466271 |  |
| *fD. cylindricum* var. *palustre* Lemmermann | Baeteo11021MS2 | Samhyeon-ri, Dong-myeon, Hongcheon-gun, Gangwon-do (37°40'13.4"N 127°54'07.4"E) | OQ453646 | | OQ453453 | OQ453366 | OQ453596 | OQ466204 | OQ466254 | OQ466303 |  |
| *D. cylindricum* var. *palustre* Lemmermann | Baeteo-Single | Samhyeon-ri, Dong-myeon, Hongcheon-gun, Gangwon-do (37°40'13.4"N 127°54'07.4"E) | - | | OQ453452 | - | - | - | - | - |  |
| *D. cylindricum* var. *palustre* Lemmermann | Bonghwa040718C | Pungsan-ri, Pungyang-myeon, Yecheon-gun, Gyeongsangbuk-do, Korea (36°28'47.6"N 128°19'06.1"E) | OQ453645 | | OQ453448 | OQ453365 | OQ453595 | OQ466203 | OQ466253 | OQ466302 |  |
| *D. cylindricum* var. *palustre* Lemmermann | Dowon111321MS2 | Dowon-ri, Toseong-myeon, Goseong-gun, Gangwon-do, Korea (38°16'09.4"N 128°28'57.6"E) | OQ453644 | | OQ453458 | OQ453364 | OQ453594 | OQ466202 | OQ466252 | OQ466301 |  |
| *D. cylindricum* var. *palustre* Lemmermann | Dowon-Single | Dowon-ri, Toseong-myeon, Goseong-gun, Gangwon-do, Korea (38°16'09.4"N 128°28'57.6"E) | - | | OQ453459 | - | - | - | - | - |  |
| *D. cylindricum* var. *palustre* Lemmermann | Inmok-Single | Insan-ri, Cheongdo-myeon, Milyang-si, Gyeongsangnam-do, Korea (35°32'16.4"N 128°38'30.5"E) | - | | OQ453451 | - | - | - | - | - |  |
| *D. cylindricum* var. *palustre* Lemmermann | Gapa120521MS4 | Gacheon-ri, Sinnyeong-myeon, Yeongcheon-si, Gyeongsangbuk-do, Korea (36°04'22.8"N 128°47'47.1"E) | OQ453643 | | OQ453455 | OQ453363 | OQ453593 | OQ466201 | OQ466251 | OQ466300 |  |
| *D. cylindricum* var. *palustre* Lemmermann | Gapa-Single | Gacheon-ri, Sinnyeong-myeon, Yeongcheon-si, Gyeongsangbuk-do, Korea (36°04'22.8"N 128°47'47.1"E) | - | | OQ453456 | - | - | - | - | - |  |
| *D. cylindricum* var. *palustre* Lemmermann | Goemokji-Single | Deoksan-ri, Maejeong-myeon, Cheongdo-gun, Gyeongsangbuk-do, Korea (35°41'43.7"N 128°47'46.7"E) | - | | OQ453454 | - | - | - | - | - |  |
| *D. cylindricum* var. *palustre* Lemmermann | Joogyo2je043021MS12 | Sinheung-ri, Namyang-myeon, Goheung-gun, Jeollanam-do, Korea (34°44'18.0"N 127°21'15.9"E) | OQ453642 | | OQ453457 | OQ453362 | OQ453592 | OQ466200 | OQ466250 | OQ466299 |  |
| *D. cylindricum* var. *palustre* Lemmermann | Meokgol-Single3 | Oun-ri, Pyeongeun-myeon, Yeongju-si, Gyeongsangbuk-do, Korea (36°44'44.5"N 128°43'50.7"E) | - | | OQ453449 | - | - | - | - | - |  |
| *D. cylindricum* var. *palustre* Lemmermann | Wolgok-Single2 | Wolgok-ri, Chojeon-myeon, Seongju-gun, Gyeongsangbuk-do, Korea (35°59'55.0"N 128°14'36.2"E) | - | | OQ453450 | - | - | - | - | - |  |
| *D. exstoundulatum* Jeong, Kim & Shin | Dallae111421MS1 | Hawolcheon-ri, Hyeonnam-myeon, Yangyang-gun, Gangwon-do, Korea (37°55'23.7"N 128°43'58.6"E) | OQ453616 | | OQ453416 | OQ453337 | OQ453566 | OQ466174 | OQ466224 | OQ466274 |  |
| *D. exstoundulatum* Jeong, Kim & Shin | Dallae-Single | Hawolcheon-ri, Hyeonnam-myeon, Yangyang-gun, Gangwon-do, Korea (37°55'23.7"N 128°43'58.6"E) | - | | OQ453417 | - | - | - | - | - |  |
| *D. faculiferum* Willén | - | Beaufort sea | JF794055 | | - | - | - | - | - | - |  |
| *D.inclinatum* Jeong, Kim & Shin | CCMP1859 | Glenmore Reservoir, near Calgary, Alberta, Canada (50.98°N -114.11°W) | OQ453620 | | OQ453439 | OQ453341 | OQ453570 | OQ466178 | OQ466228 | OQ466278 |  |
| *D.inclinatum* Jeong, Kim & Shin | CCMP1860 | lower end of West Harbor Pond, West Boothbay Harbor, Maine, USA (43.847°N -69.647°W) | OQ453621 | | OQ453440 | OQ453342 | OQ453571 | OQ466179 | OQ466229 | OQ466279 |  |
| *D.inclinatum* Jeong, Kim & Shin | CCMP2766 | Glenmore Reservoir, near Calgary, Alberta, Canada (50.98°N -114.11°W) | OQ453622 | | OQ453441 | OQ453343 | OQ453572 | OQ466180 | OQ466230 | OQ466280 |  |
| *D.inclinatum* Jeong, Kim & Shin | Chojeon011219A | Songjeong-ri, Mijo-myeon, Namhae-gun, Gyeongsangnam-do, Korea (34°43'45.1"N 128°01'47.3"E) | OQ453623 | | OQ453443 | OQ453344 | OQ453573 | OQ466181 | OQ466231 | OQ466281 |  |
| *D.inclinatum* Jeong, Kim & Shin | Dogwan033018A | Sambun-ri, Dain-myeon, Uiseong-gun, Gyeongsangbuk-do, Korea (36°25'16.0"N 128°24'15.4"E) | OQ453624 | | OQ453447 | OQ453345 | OQ453574 | OQ466182 | OQ466232 | OQ466282 |  |
| *D.inclinatum* Jeong, Kim & Shin | Hwalgol120318D | Gacheon-ri, Cheongri-myeon, Sangu-si, Gyeongsangbuk-do, Korea (35°33'59.3"N 126°44'18.8"E) | OQ453626 | | OQ453444 | OQ453346 | OQ453575 | OQ466183 | OQ466233 | OQ466283 |  |
| *D.inclinatum* Jeong, Kim & Shin | Jijije022318A | Banwol-ri, Baekgu-myeon, Gimje-si, Jeollabuk-do, Korea  (35°53'07.4"N 126°57'43.8"E) | OQ453625 | | OQ453445 | OQ453347 | OQ453576 | OQ466184 | OQ466234 | OQ466284 |  |
| *D.inclinatum* Jeong, Kim & Shin | Napji-Single | Jukgok-ri, Hwanam-myeon, Yeongcheon-si, Gyeongsangbuk-do, Korea (36°03'30.2"N 128°57'44.3"E) | - | | OQ453442 | - | - | - | - | - |  |
| *D.inclinatum* Jeong, Kim & Shin | Taegok-Single | Songnae-ri, Jeomgok-myeon, Euiseong-gun, Gyeongsangbuk-do, Korea (36°26'08.5"N 128°44'04.2"E) | - | | OQ453446 | - | - | - | - | - |  |
| *D. ningwuensis* Jiang, Feng & Xie | Hwasan-Single | Yeonjeong-ri, Shinnyeong-myeon, Yeongcheon-si, Gyeongsangbuk-do, Korea (36°03'32.8"N 128°48'52.6"E) | - | | OQ453479 | - | - | - | - | - |  |
| *D. ningwuensis* Jiang, Feng & Xie | Japung-Single | Japoong-ri, Hampyeong-eup, Hampyeong-gun, Jeollanam-do, Korea (35°01'40.4"N 126°28'30.7"E) | - | | OQ453482 | - | - | - | - | - |  |
| *D. ningwuensis* Jiang, Feng & Xie | Myeoseul031718C | Yangsa-ri, Bibong-myeon, Cheongyang-gun, Chuncheongnam-do, Korea (36°29'17.0"N 126°45'44.9"E) | OQ453649 | | OQ453481 | OQ453369 | OQ453599 | OQ466207 | OQ466257 | OQ466306 |  |
| *D. ningwuensis* Jiang, Feng & Xie | NW201610 | Lake Pipahai, Shanxi province, China (38°51’40”N, 112°13’7”E) | MH121037 | | - | - | - | - | - | - |  |
| *D. ningwuensis* Jiang, Feng & Xie | Yeokjae092718B | Goam-ri, Hongseong-eup, Honseong-gun, Chungcheongnam-do, Korea  (36°35'35.1"N 126°40'33.4"E) | OQ453647 | | OQ453480 | OQ453367 | OQ453597 | OQ466205 | OQ466255 | OQ466304 |  |
| *D. ningwuensis* Jiang, Feng & Xie | Yeongrangho111321MS1 | Jangsa-dong, Sokcho-si, Gwangwon-do, Korea (38°12'54.1"N 128°34'13.1"E) | OQ453648 | | OQ453484 | OQ453368 | OQ453598 | OQ466205 | OQ466256 | OQ466305 |  |
| *D. ningwuensis* Jiang, Feng & Xie | Yeongrangho-Single | Jangsa-dong, Sokcho-si, Gwangwon-do, Korea (38°12'54.1"N 128°34'13.1"E) | - | | OQ453483 | - | - | - | - | - |  |
| *D. ningwuensis* Jiang, Feng & Xie | 171027 | unknown | MK464017 | | MK487765 | - | - | MK550498 | - | - |  |
| *D. pediforme* (Lemmerman) Steinecke | LO226KS | Lake Fuschlsee, Salzkammergut, Austria | KJ913667 | | | - | - | - | - |  |  |
| *D. pediforme* (Lemmerman) Steinecke | LO236_1 | Lake Fuschlsee, Salzkammergut, Austria | KJ579347 | | | - | - | - | - | - |  |
| *D. pediforme* (Lemmerman) Steinecke | LO128-16 | Lake Loibersbacher Teich 1, Salzkammergut, Austria | EU024992 | | - | - | - | - | - | - |  |
| *D. pediforme* (Lemmerman) Steinecke | LO134-19 | Lake Loibersbacher Teich 1, Salzkammergut, Austria | EU025000 | | - | - | - | - | - | - |  |
| *D. pediforme* (Lemmerman) Steinecke | LO228-77 | Lake Loibersbacher Teich 2, Salzkammergut, Austria | EU025009 | | - | - | - | - | - | - |  |
| *D. sertularia* var. *thyrsoideum* (Chodat) Lemmermann | Chosan041710C | Doam-ri, Dain-myeon, Uiseong-gun, Gyeongsangbuk-do, Korea (36°27'37.0"N 128°21'38.9"E) | OQ453639 | | OQ453436 | OQ453359 | OQ453589 | OQ466197 | OQ466247 | OQ466296 |  |
| *D. sertularia* var. *thyrsoideum* (Chodat) Lemmermann | Geumgang111321MS6 | Chodo-ri, Hyeonnae-myeon, Goseong-gun, Gangwon-do, Korea (38°28'58.6"N 128°25'53.5"E) | OQ453640 | | OQ453438 | OQ453360 | OQ453590 | OQ466198 | OQ466248 | OQ466297 |  |
| *D. sertularia* var. *thyrsoideum* (Chodat) Lemmermann | Geumgang-Single | Chodo-ri, Hyeonnae-myeon, Goseong-gun, Gangwon-do, Korea (38°28'58.6"N 128°25'53.5"E) | - | | OQ453437 | - | - | - | - | - |  |
| *D. sertularia* var. *thyrsoideum* (Chodat) Lemmermann | Yanghari040921MS12 | Sinyang-ri, Nohwa-eup, Wando-gun, Jeollanam-do, Korea (34°12'36.5"N 126°34'45.5"E) | OQ453641 | | OQ453435 | OQ453361 | OQ453591 | OQ466199 | OQ466249 | OQ466298 |  |
| *D. similis* Jeong, Kim & Shin | Beolmot-Single1 | Sacheon-ri, Hwanam-myeon, Yeongcheon-si, Gyeongsangbuk-do, Korea (36°03'02.2"N 128°54'51.5"E) | - | | OQ453418 | - | - | - | - | - |  |
| *D. similis* Jeong, Kim & Shin | Macheon-Single | Jigok-ri, Pyeongeun-myeon, Yeongju-si, Gyeongsangbuk-do, Korea (36°42'13.7"N 128°44'04.9"E) | - | | OQ453424 | - | - | - | - | - |  |
| *D. similis* Jeong, Kim & Shin | Meokgol112021MS16 | Oun-ri, Pyeongeun-myeon, Yeongju-si, Gyeongsangbuk-do, Korea (36°44'44.5"N 128°43'50.7"E) | OQ453619 | | OQ453421 | OQ453340 | OQ453569 | OQ466177 | OQ466227 | OQ466277 |  |
| *D. similis* Jeong, Kim & Shin | Meokgol-Single2 | Oun-ri, Pyeongeun-myeon, Yeongju-si, Gyeongsangbuk-do, Korea (36°44'44.5"N 128°43'50.7"E) | - | | OQ453422 | - | - | - | - | - |  |
| *D. similis* Jeong, Kim & Shin | Sanseong2ji102318A | Sanseong-ri, Janggok-myeon, Hongseong-gun, Chungcheongnam-do, Korea (36°29'33.7"N 126°44'19.9"E) | OQ453617 | | OQ453420 | OQ453338 | OQ453567 | OQ466175 | OQ466225 | OQ466275 |  |
| *D. similis* Jeong, Kim & Shin | Goolun-Single | Goolun-ri, Hwachon-myeon, Hongcheon-gun, Gangwon-do, Korea (37°43'50.2"N 127°57'36.7"E) | - | | OQ453423 | - | - | - | - | - |  |
| *D. similis* Jeong, Kim & Shin | Goemok010822MS2 | Deoksan-ri, Maejeong-myeon, Cheongdo-gun, Gyeongsangbuk-do, Korea (35°41'43.7"N 128°47'46.7"E) | OQ453618 | | OQ453419 | OQ453339 | OQ453568 | OQ466176 | OQ466226 | OQ466276 |  |
| *D. sociale* Ehrenberg | Angol061922MS3 | Soo-ri, Sosu-myeon, Goesan-gun, Chungcheongbuk-do, Korea (36°51'59.4"N 127°45'18.0"E) | OQ453615 | | OQ453415 | OQ453336 | OQ453565 | OQ466173 | OQ466223 | OQ466273 |  |
| *D. sociale* Ehrenberg | Daejirangmot-Single | Samgui-ri, Cheongri-myeon, Sangju-si, Gyeongsangbuk-do, Korea (36°21'48.6"N 128°07'05.2"E) | - | | OQ453414 | - | - | - | - | - |  |
| *D. sociale* Ehrenberg | Gaejeong-Single | Donam-ri, Daega-myeon, Seongju-gun, Gyeongsangbuk-do, Korea (35°54'14.4"N 128°11'06.4"E) | - | | OQ453413 | - | - | - | - | - |  |
| *D. sociale* Ehrenberg | FU32_4 | Lake Fuschlsee, Salzkammergut, Austria | KJ579297 | | | - | - | - | - | - |  |
| *D. sociale* Ehrenberg | FU35_5 | Lake Fuschlsee, Salzkammergut, Austria | KJ579299 | | | - | - | - | - | - |  |
| *D. sociale* Ehrenberg | FU38_1 | Lake Fuschlsee, Salzkammergut, Austria | KJ579300 | | | - | - | - | - | - |  |
| *D. sociale* Ehrenberg | 180802 | unknown | MK464020 | | MK487766 | - | - | - | - | - |  |
| *Dinobryon* sp. | OE28KV | Lake Ödensee, Austria | KX100621 | | - | KX100746 | - | - | - | - |  |
| *D. spinum* Jeong, Kim & Shin | Arong051421MS2 | Jikdong-ri, Jungdong-myeon, Yeongwol-gun, Gangwon-do, Korea (37°11'18.5"N 128°48'32.7"E) | OQ453633 | | OQ453428 | OQ453354 | OQ453583 | OQ466191 | OQ466241 | OQ466291 |  |
| *D. spinum* Jeong, Kim & Shin | Deokghi042118B | Deokghi-dong. Iksan-si, Jeollabuk-do, Korea (35°58'42.6"N 127°02'07.2"E) | OQ453634 | | OQ453430 | OQ453355 | OQ453584 | OQ466192 | OQ466242 | OQ466292 |  |
| *D. spinum* Jeong, Kim & Shin | Geumgok020610D | Geumseong-ri, Hamla-myeon, Iksan-si, Jeollabuk-do, Korea (36°04'14.2"N 126°55'05.9"E) | OQ453636 | | OQ453431 | OQ453357 | OQ453586 | OQ466194 | OQ466243 | OQ466293 |  |
| *D. spinum* Jeong, Kim & Shin | Gwangdaeje031221MS4 | Gwangdae-ri, Bigeum-myeon, Sinan-gun, Jeollanam-do, Korea (34°47'16.3"N 125°59'05.2"E) | OQ453635 | | OQ453429 | OQ453356 | OQ453585 | OQ466193 | OQ466243 | OQ466294 |  |
| *D. spinum* Jeong, Kim & Shin | Myeoseul031718A | Yangsa-ri, Bibong-myeon, Cheongyang-gun, Chuncheongnam-do, Korea (36°29'17.0"N 126°45'44.9"E) | OQ453637 | | OQ453432 | OQ453358 | OQ453587 | OQ466195 | OQ466245 | OQ466295 |  |
| *D. spinum* Jeong, Kim & Shin | Wonyong122822S2 | Yongdu-ri, Jeongsan-myeon, Cheongyang-gun, Chungcheongnam-do, Korea (36°25'12.5"N 126°56'15.0"E) | OQ453638 | | OQ453433 | - | OQ453588 | OQ466196 | OQ466246 | - |  |
| *D. spinum* Jeong, Kim & Shin | Wonyong122822S5 | Yongdu-ri, Jeongsan-myeon, Cheongyang-gun, Chungcheongnam-do, Korea (36°25'12.5"N 126°56'15.0"E) | - | | OQ453434 | - | - | - | - | - |  |
| *D. taiyuanensis* Jiang, Feng & Xie | Baekwoon-Single | Nongsang-ri, Gogeum-myeon, Wando-gun, Jeollanam-do, Korea (34°23'50.6"N 126°47'19.0"E) | - | | OQ453460 | - | - | - | - | - |  |
| *D. taiyuanensis* Jiang, Feng & Xie | Beolmot-Single2 | Sacheon-ri, Hwanam-myeon, Yeongcheon-si, Gyeongsangbuk-do, Korea (36°03'02.2"N 128°54'51.5"E) | - | | OQ453478 | - | - | - | - | - |  |
| *D. taiyuanensis* Jiang, Feng & Xie | Booya-Single | Booya-ri, Cheongdo-eup, Cheongdo-gun, Gyeongsangbuk-do, Korea (35°40'12.0"N 128°47'04.9"E) | - | | OQ453467 | - | - | - | - | - |  |
| *D. taiyuanensis* Jiang, Feng & Xie | Cheonma111321MS4 | Yugok-ri, Bonghwa-eup, Bonghwa-gun, Gyeongsangbuk-do, Korea (36°54'40.3"N 128°46'08.4"E) | OQ453631 | | OQ453475 | OQ453352 | OQ453581 | OQ466189 | OQ466239 | OQ466289 |  |
| *D. taiyuanensis* Jiang, Feng & Xie | Cheonma-Single | Yugok-ri, Bonghwa-eup, Bonghwa-gun, Gyeongsangbuk-do, Korea (36°54'40.3"N 128°46'08.4"E) | - | | OQ453476 | - | - | - | - | - |  |
| *D. taiyuanensis* Jiang, Feng & Xie | Daenae-Single | Shinho-ri, Hwanam-myeon, Yeongcheon-si, Gyeongsangbuk-do, Korea (36°02'33.4"N 128°56'28.7"E) | - | | OQ453462 | - | - | - | - | - |  |
| *D. taiyuanensis* Jiang, Feng & Xie | Dogwan022718C | Sambun-ri, Dain-myeon, Uiseong-gun, Gyeongsangbuk-do, Korea  (36°25'16.0"N 128°24'15.4"E) | OQ453632 | | OQ453473 | OQ453353 | OQ453582 | OQ466190 | OQ466240 | OQ466290 |  |
| *D. taiyuanensis* Jiang, Feng & Xie | Dogwan022718HM1 | Sambun-ri, Dain-myeon, Uiseong-gun, Gyeongsangbuk-do, Korea  (36°25'16.0"N 128°24'15.4"E) | OQ453627 | | OQ453472 | OQ453348 | OQ453577 | OQ466185 | OQ466235 | OQ466285 |  |
| *D. taiyuanensis* Jiang, Feng & Xie | Donggo-Single | Seolmae-ri, Gunnam-myeon, Yeonggwang-gun, Jeollanam-do, Korea (35°13'44.0"N 126°25'11.6"E) | - | | OQ453465 | - | - | - | - | - |  |
| *D. taiyuanensis* Jiang, Feng & Xie | Dongsong-Single | Dongsong-ri, Gaepo-myeon, Yecheon-gun, Gyeongsangbuk-do, Korea (36°36'03.2"N 128°24'18.7"E) | - | | OQ453463 | - | - | - | - | - |  |
| *D. taiyuanensis* Jiang, Feng & Xie | Goochon-Single | Dojang-ri, Jibo-myeon, Yecheon-gun, Gyeongsangbuk-do, Korea (36°31'27.8"N 128°23'42.4"E) | - | | OQ453461 | - | - | - | - | - |  |
| *D. taiyuanensis* Jiang, Feng & Xie | Gwangok-Single | Anin-ri, Cheongdo-eup, Cheongdo-gun, Gyeongsangbuk-do, Korea (35°40'40.4"N 128°45'39.9"E) | - | | OQ453474 | - | - | - | - | - |  |
| *D. taiyuanensis* Jiang, Feng & Xie | Josoo1ri111620MS1 | Josu-ri, Hangyeong-myeon, Jeju-si, Jeju-do, Korea (33°20'08.7"N 126°13'43.3"E) | OQ453628 | | OQ453470 | OQ453349 | OQ453578 | OQ466186 | OQ466236 | OQ466286 |  |
| *D. taiyuanensis* Jiang, Feng & Xie | Myeongdo-Single | Sanggye-ri, Yeomsan-myeon, Yeonggwang-gun, Jeollanam-do, Korea (35°14'01.3"N 126°23'48.1"E) | - | | OQ453469 | - | - | - | - | - |  |
| *D. taiyuanensis* Jiang, Feng & Xie | Naegok-Single | Oonsan-ri, Cheongdo-eup, Cheongdo-gun, Gyeongsangbuk-do, Korea (35°41'27.6"N 128°46'49.4"E) | - | | OQ453464 | - | - | - | - | - |  |
| *D. taiyuanensis* Jiang, Feng & Xie | Wonyong111618A | Yongdu-ri, Jeongsan-myeon, Cheongyang-gun, Chungcheongnam-do, Korea (36°25'12.5"N 126°56'15.0"E) | OQ453630 | | OQ453471 | OQ453351 | OQ453580 | OQ466188 | OQ466238 | OQ466288 |  |
| *D. taiyuanensis* Jiang, Feng & Xie | Yeongok-Single | Booya-ri, Cheongdo-eup, Cheongdo-gun, Gyeongsangbuk-do, Korea (35°39'29.9"N 128°46'41.2"E) | - | | OQ453466 | - | - | - | - | - |  |
| *D. taiyuanensis* Jiang, Feng & Xie | Yookoje031321MS4 | Sinjang-ri, Aphae-eup, Sinan-gun, Jeollanam-do, Korea (34°50'21.7"N 126°21'25.2"E) | OQ453629 | | OQ453477 | OQ453350 | OQ453579 | OQ466187 | OQ466237 | OQ466287 |  |
| *D. taiyuanensis* Jiang, Feng & Xie | Yucheon-Single | Shinga-ri, Geumcheon-myeon, Naju-si, Jeollanam-do, Korea (35°02'37.0"N 126°46'30.0"E) | - | | OQ453468 | - | - | - | - | - |  |
| *D. taiyuanensis* Jiang, Feng & Xie | 180416 | Linde lake, Shanxi province, China (37°80’29”N, 112°59’7”E) | MK464015 | | MK487763 |  |  |  |  |  |  |
| **Outgroup** |  |  |  | |  |  |  |  |  |  |  |
| *Mallomonas splendens* | CCMP1782 | Mount Macedon, Victoria, Australia (37°28'37.2"S 144°33'36.0"E) | JQ955668 | | - | JQ955673 | KM817993 | JQ955663 | KM818071 | OQ557082 |  |
| *Neotessella volvocina* | CCMP1781 | Billabong, Yarra River, Melbourne, Victoria, Australia (34°16'00.1"S 142°13'00.1"E) | EF165119 | | - | KM590691 | KM590765 | EF165199 | KM590834 | OQ557083 |  |
| *Synura petersenii* | S114.C7 | Sweden | OQ549918 | | - | OQ549917 | MH795128 | | | OQ557084 |  |
