# Supplementary material for: Multigene phylogeny reveals a cryptic diversity in the genus Dinobryon (Chrysophyceae) with integrative description of five new species
Source: Front Plant Sci. 2023 Apr 18;14:1150814. doi: 10.3389/fpls.2023.1150814 (PMC10151809; doi:10.3389/fpls.2023.1150814)
Supplement: Supplementary file 4 [file Table_2.docx]

**Supplementary table 2.** Oligonucleotide primer sequences for the PCR amplification and sequencing of each gene.

| Gene | Pimer name | Sequences (5’ to 3’) | Direction | Reference |
| --- | --- | --- | --- | --- |
| Nuclear SSU | EukA | AAC CTG GTT GAT CCT GCC AGT | Forward | Medlin et al. (1988) |
|  | F-566 | CAG CAG CCG CGG TAA TTC C | Forward | Hadziavdic et al. (2014) |
|  | R-1200 | CCC GTG TTG AGT CAA ATT AAG C | Reverse | Hadziavdic et al. (2014) |
|  | EukB | TGA TCC TTC TGC AGG TTC ACC TAC | Reverse | Medlin et al. (1988) |
| Nuclear ITS | ITS_DF | CGC ACC TAC CGA TTG AAT | Forward | Jeong et al. (2021) |
|  | ITS_DR | CCT CCG CCT AGT TAT ATG CTT A | Reverse | Jeong et al. (2021) |
| Nuclear LSU | 28S_25F | ACC CGC TGA ATT TAA GCA TAT A | Forward | Jo et al. (2011) |
|  | 28S_736F | CCC GAA AGA TGG TGA ACT C | Forward | Jo et al. (2011) |
|  | 28S_2038F | GAC AAG GGG AAT CCG ACT | Forward | Jo et al. (2011) |
|  | 28S_861R | GTT CGA TTA GTC TTT CGC CCC T | Reverse | Jo et al. (2011) |
|  | 28S_2160R | CCG CGC TTG GTT GAA TTC | Reverse | Jo et al. (2011) |
|  | 28S_2719R | AAG AAT CAA AAA GCA ACG TCG C | Reverse | In this study |
| Plastid LSU | Chr23S_18F | GCT TGY RAR GGA TCC CTW GGT | Forward | In this study |
|  | Spu23S_790F | CAA TCG AAA CCG GAG CTA | Forward | In this study |
|  | Dino23S_1690F | TGC CGG AAG GTT AAA GAA G | Forward | In this study |
|  | Dino23S_790R | TAG CTC CGG TTT CGA TTG | Reverse | In this study |
|  | Spu23S_1754R | GCT AAA CAG TTG CTT GGA ACT T | Reverse | In this study |
|  | 23S_2742R | GGG CTT CCT ACT TAG ATG CTT T | Reverse | Jo et al. (2011) |
| Plastid *rbc*L | DrbcLF | TGG ACK GTW GTW TGG ACW GAT | Forward | In this study |
|  | DrbcLR | TTA AGA TGC AGA TTS AGT TGG TGT | Reverse | In this study |
| Plastid *psa*A | psaA_130F | AAC WAC TTG GAT TTG GAA | Forward | Jo et al. (2016) |
|  | psaA_580F | CGT GAA TTT ATY TCM ACT TTA TA | Forward | In this study |
|  | psaA_700R | CAT CTG TTA ACC AAA GAC TTG C | Reverse | In this study |
|  | psaA_1760R | CCT CTW CCW GGW CCA TCR CAW GG | Reverse | Jo et al. (2016) |
| Mitochondria CO1 | Coxf_Mod | TCT AGY RTA TTA GGA ACM ACK ATG TC | Forward | In this study |
|  | DinoCox1_109F | CWC ARC CRG GWA AYC ARA T | Forward | In this study |
|  | DinoCox1_1382R | TTC CAW CCW GMR TAW GCR TC | Reverse | In this study |
|  | DinoCox1_1393R | GCY AMA GAA TTC CAA CCR AA | Reverse | In this study |
