# Supplementary material for: Multigene phylogeny reveals a cryptic diversity in the genus Dinobryon (Chrysophyceae) with integrative description of five new species
Source: Front Plant Sci. 2023 Apr 18;14:1150814. doi: 10.3389/fpls.2023.1150814 (PMC10151809; doi:10.3389/fpls.2023.1150814)
Supplement: Supplementary file 5 [file Table_3.docx]

**Supplementary Table 3.** Summary of lorica morphological characteristics of colonial *Dinobryon* species. The numbers in parentheses indicate the investigated number of cells. (-) denotes unrecorded information in literatures.

| **Taxon** | **Strain** | **Habitat** | **Adjoining of daughter cell** | **Lorica morphology** | | | | | |
| --- | --- | --- | --- | --- | --- | --- | --- | --- | --- |
|  |  |  |  | Opening | Upper part | Lower part | Length (µm) | Width  (µm) | Opening width (µm) |
| *D. anulatum*^22^ |  | Freshwater | Internal wall of mother cell | Widened. | Straight or widened to transition region from opening, undulated | Obliquely narrowed to pointed or flattened end | 21.0-27.0 | 8.0-11.0 | - |
|  |  |  | 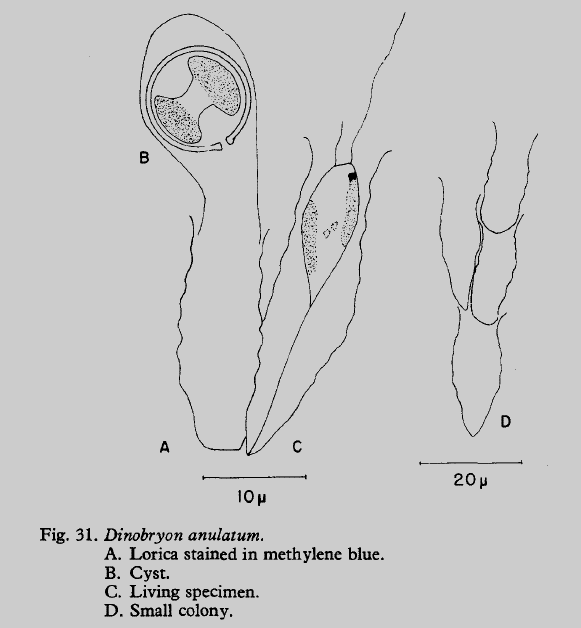(Figs. 31A-D in Hillard & Asmund, 1963) | | | | | | |
| *D. asymmetricum*^22^ |  | Freshwater | External wall of mother cell | Widened. | Straight or widened to transition region from opening, with or without undulation | Obliquely narrowed to pointed or flattened end, with irregular bulging | 23.0-55.0 | 6.0-10.0 | - |
|  |  |  | 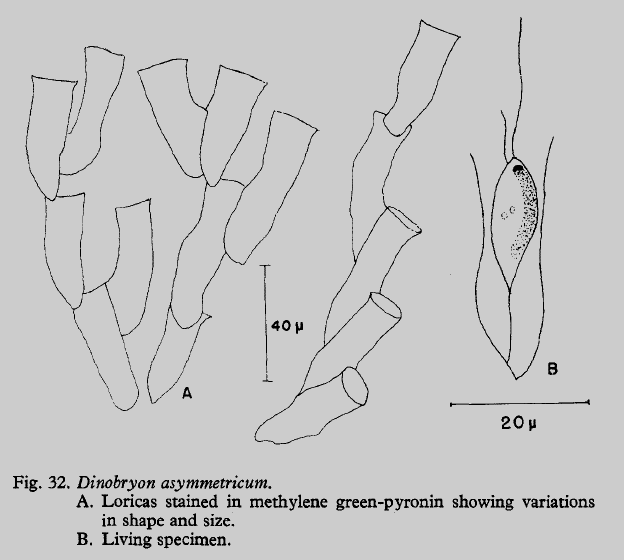 (Figs. 32A-B in Hillard & Asmund, 1963) | | | | | | |
| *D. balticum*^8,18^ |  | ^8^Marine | Internal wall of mother cell | Widened. | Straight to transition region from opening | Obliquely narrowed to pointed end | 50.0-64.0 | 3.0-4.0 | 5.0-6.0 |
|  |  |  | 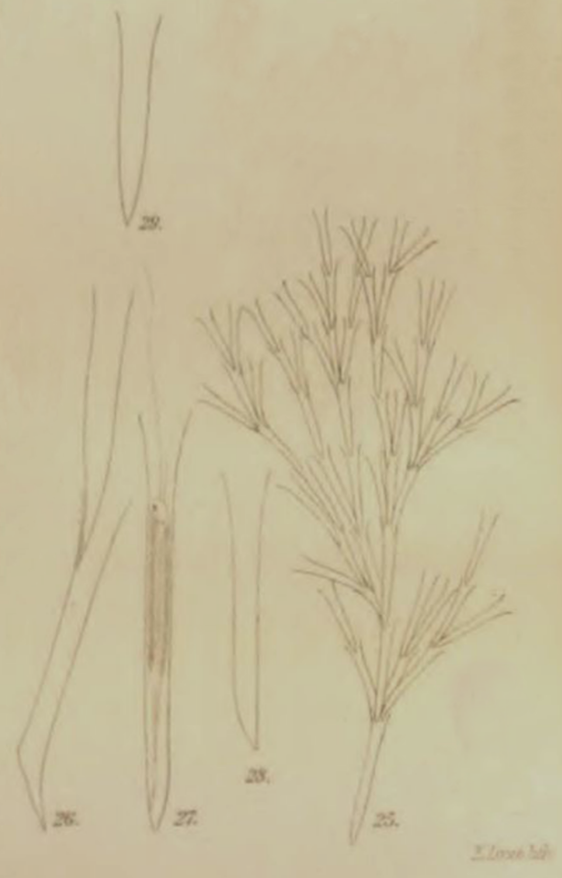 (Table XVIII, Figs. 25-29 in Lemmermann, 1901a) | | | | | | |
|  |  | ^18^Marine | Internal wall of mother cell | Widened | Straight to transition region from opening | Obliquely narrowed to pointed end | 50.0-66.0 | - | 3.0-5.0 |
|  |  |  | 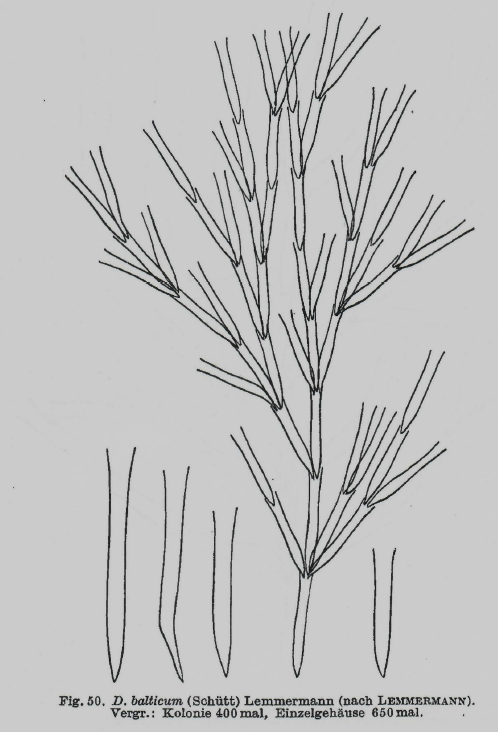(Fig. 50 in Kreiger, 1930) | | | | | | |
| *D. bavaricum*^4,7,8,11,13,14,18,19,33^ |  | ^4^Freshwater |  | Widened. | Slightly widened to transition region from opening, undulated | Attenuate to a long stalk with a sharply pointed end | 80.0-88.0 | 9.2 | - |
|  |  | ^7^Freshwater | Internal wall of mother cell | Widened | Straight to transition region from opening, undulated | Attenuate to a long stalk with a sharply pointed end | 49.0-100.0 | 7.0-9.0 | - |
|  |  |  | 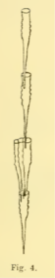 (Fig. 4 in Brunnthaler, 1901) | | | | | | |
|  |  | ^8^Freshwater | - | Widened | Straight to transition region from opening | Attenuate to a long stalk with a sharply pointed end | 80.0-90.0 | 8.0-9.0 | - |
|  |  |  | 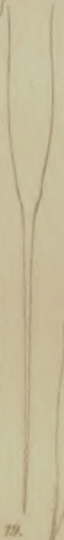 (Table XVIII, Fig. 19 in Lemmermann, 1901a) | | | | | | |
|  |  | ^11^Freshwater | - | - | Straight to transition region from opening, undulated | Attenuate to a long stalk with a sharply pointed end | 46.5-100.0 | - | - |
|  |  |  | 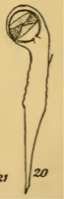 (Fig. 20 in Lemmermann, 1910) | | | | | | |
|  |  | ^13^Freshwater | Internal wall of mother cell | Straight or widened | Straight to transition region from opening, undulated | Attenuate to a long stalk with a sharply pointed end | 90-102 | - | - |
|  |  |  | 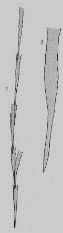 (Table XV, Figs. 1-2 in Bachmann, 1911) | | | | | | |
|  |  | ^14^Freshwater | - | Straight | Straight to transition region from opening, undulated | Attenuate to a long stalk with a sharply pointed end | 45-100 | - | - |
|  |  |  | 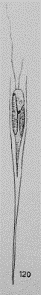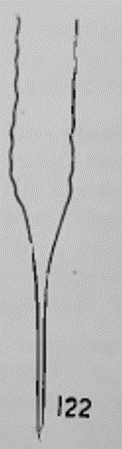 (Fig. 120, 122 in Pascher, 1913) | | | | | | |
|  |  | ^18^Freshwater | Internal wall of mother cell | Straight or widened | Suddenly or gradually narrowed to transition region, undulated | Attenuate to a long stalk with a sharply pointed end | 50-120 | 6-10 | - |
|  |  |  | 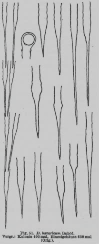 (Fig. 51 in Kreiger, 1930) | | | | | | |
|  |  | ^19^Freshwater | Internal wall of mother cell | Widened | Straight to transition region from opening, weakly or strongly undulated depending on individual | Attenuate to a long stalk with a sharply pointed end | 38.0-119.0 | 6.5-10.0 | - |
|  |  |  | 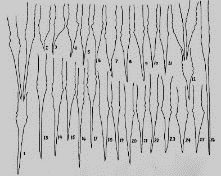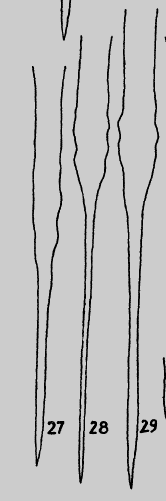  (Plate III, Figs. 1-29 in Ahlstrom, 1937) | | | | | | |
|  | CCMP3054 (n=25) | ^33^Freshwater | Internal wall of mother cell | Straight or widened | Straight to transition region or narrowed to middle of upper part, then widened to transition region, undulated | Attenuate to a long, or short stalk with a sharply pointed end | 34.4-71.7 | 7.4-11.1 | 6.9-10.4 |
|  |  |  | 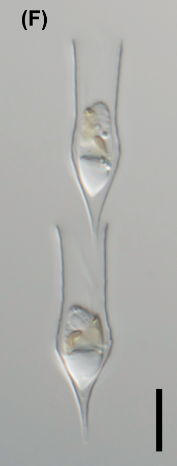 (Fig. 1F in this study. Scale bar = 10 µm) | | | | | | |
|  | CCMP3270 (n=25) | ^33^Freshwater | Internal wall of mother cell | Straight or widened | Straight to transition region or narrowed to middle of upper part, then widened to transition region, undulated | Attenuate to a long, or short stalk with a sharply pointed end | 29.0-66.8 | 7.3-9.5 | 7.0-9.9 |
|  |  |  | 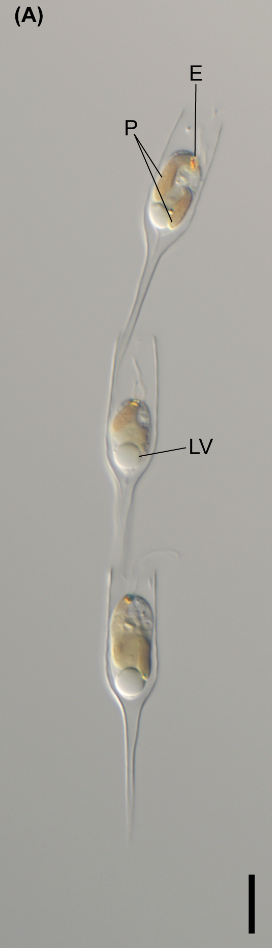 (Fig. 1A in this study. Scale bar = 10 µm) | | | | | | |
|  | CCMP2884 (n=25) | ^33^Freshwater | Internal wall of mother cell | Widened flaring | Narrowed to middle of upper part, then widened to transition region | Attenuate to relatively short, sharply pointed end | 24.5-35.1 | 6.7-9.4 | 7.2-11.8 |
|  |  |  | 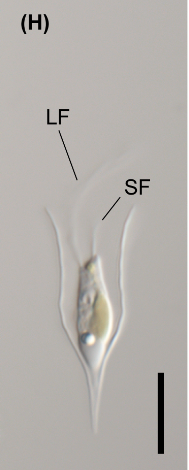 (Fig. 1H in this study. Scale bar = 10 µm) | | | | | | |
| *D. bavaricum* var. *medium*^14,18^ |  | ^14^Freshwater | Internal wall of mother cell | Widened | Straight to transition region undulated | Attenuate to end from transition region, undulated | - | - | - |
|  |  |  | 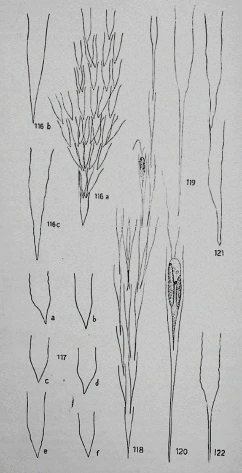 (Fig. 121 in Pashcer, 1913) | | | | | | |
|  |  | ^18^reshwater | Internal wall of mother cell | Straight or widened | Straight to transition region or narrowed to middle of upper part, then widened to transition region, undulated | Attenuate to lanceolate end from transition region | 50.0-90.0 | 7.0-8.0 | - |
|  |  |  | 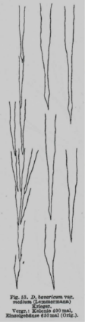 (Fig. 53, Kreiger, 1930) | | | | | | |
| *D. bavaricum* var. *vanhöffenii*^16,18,19^ |  | ^16^Freshwater | Internal wall of mother cell | Widened | Slightly narrowed to middle of upper part from opening, then widened to transition region, sometimes undulated | Attenuate to lanceolate end from transition region | 100-120 | 8 | - |
|  |  | ^18^Freshwater | Internal wall of mother cell | Straight or widened | Slightly widened to transition region from opening, undulated | Attenuate to lanceolate end from transition region, end of its lower sometimes bent | 50.0-80.0 | 8.0-10.0 |  |
|  |  |  | 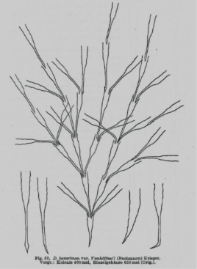 (Fig.52 in Kreiger, 1930) | | | | | | |
|  |  | ^19^Freshwater | - | Widened flaring | Slightly narrowed to middle of upper part from opening, then widened to transition region, undulated | Attenuate to lanceolate end from transition region, end of its lower sometimes bent | 60.0-110.0 | 11.0-13.3 | - |
|  |  |  | 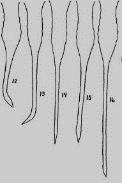 (Plate IV, Figs. 12-16 in Ahlstrom, 1937) | | | | | | |
| *D. behningii*^18^ |  | Freshwater | Internal wall of mother cell | Obliquely narrowed to pointed end having spine from widened opening; funnel shape. | | | 56.0-61.0 | - | 15.0-25.5 |
|  |  |  | 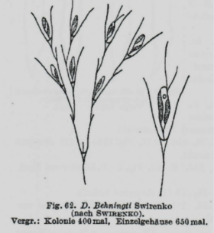 (Fig. 62 in Kreiger, 1930) | | | | | | |
| *D. belgicae*^12,24^ |  | ^12^Marine | Internal wall of mother cell | Widened, slightly flaring | Straight to transition region from opening | Obliquely narrowed to pointed end | - | - | - |
|  |  |  | 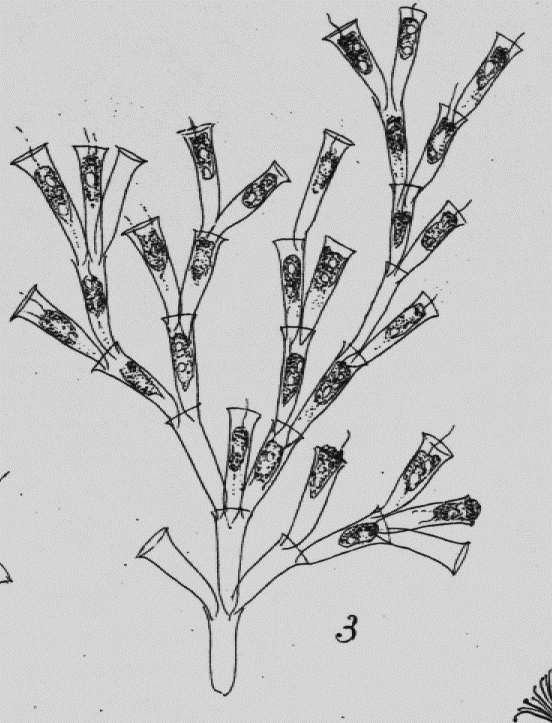 (Fig. 3 in Meunier, 1910) | | | | | | |
|  |  | ^24^Marine | Internal wall of mother cell | Widened | Straight to transition region from opening | Obliquely narrowed to apex from transition region. | 24.0 | 7.0 | - |
| *D. campanulostipitum*^19^ |  | Freshwater | Internal wall of mother cell | Widened flaring | Widened to transition region from opening, undulated | Obliquely narrowed from transition region, then sharply narrowed to blunt apex | 25.0-54.0 | - | 10.0-11.5 |
|  |  |  | 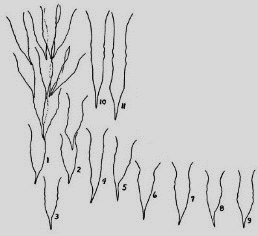 (Plate IV, Figs. 1-11 in Ahlstrom, 1937) | | | | | | |
| *D. coalescens*^14,18^ |  | ^14^Marine | External wall of mother cell | Widened flaring | Slightly narrowed to middle of upper part from opening, then widened to transition region | Narrowed from transition region, then to be a cone or long stem. | 50.0-60.0 | - | - |
|  |  |  | 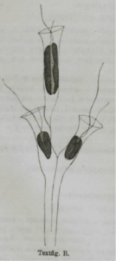 (Fig. B in Schiller, 1925) | | | | | | |
|  |  | ^18^Marine | External wall of mother cell | Widened | Slightly narrowed to middle of upper part from opening, then widened to transition region | Narrowed from transition region, then to be a cone or long stem. | 50.0-60.0 | - | - |
|  |  |  | 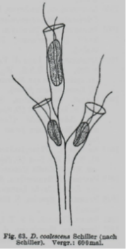 (Fig. 63 in Kreiger, 1930) | | | | | | |
| *D. crenulatum*^10,21^ |  | ^10^Freshwater | - | Widened | Slightly widened to transition region from opening, undulated | Obliquely narrowed to sharply pointed, spine-like end | 31.0-32.0 | 9.5-10.0 | 8.0-8.5 |
|  |  |  | 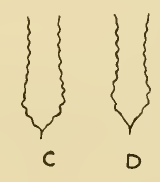 (Figs. 7C-D in West & West, 1909) | | | | | | |
|  |  | ^21^Freshwater | Internal wall of mother cell | Widened | Straight to transition region from opening, undulated | Obliquely narrowed to sharply pointed end | 30.0-50.0 | 8.0-10.0 | - |
|  |  |  | 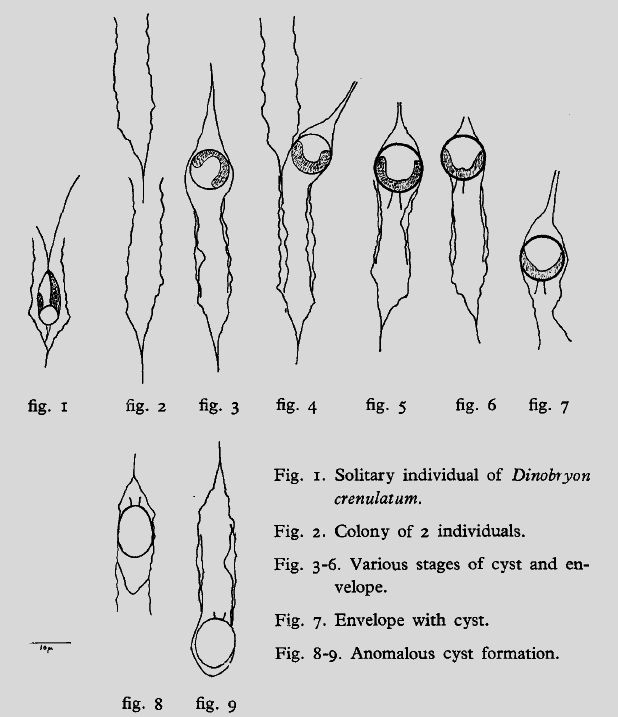 (Figs. 1-9 in Asmund, 1955a) | | | | | | |
| *D. cylindricollarium*^33^ | Myeoseul111618D (n=25) | Freshwater | Internal wall of mother cell | Widened | Straight to transition region or slightly narrowed to the middle of upper part, then widened to transition region from opening, undulated | Abruptly attenuate to pointed end | 37.7-52.6 | 7.2-8.6 | 5.6-8.2 |
| *D. cylindricum*^3,5,6,7,9,11,13,14,18,19^ |  | ^3^Freshwater | - | Widened | Straight to transition region from opening. | Sometimes slightly curved, obliquely narrowed to pointed end | 84.0-118.0 | 10.0 | - |
|  |  | ^5^Freshwater | - | Widened | Straight to transition region or slightly narrowed to then middle of upper part, then widened to transition region from opening | Obliquely narrowed to pointed end | 70.0-75.0 | 10.0-12.0 | - |
|  |  |  | 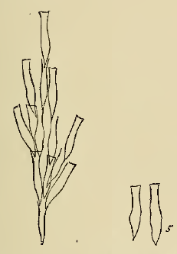 (Figs. 1, 5 in Chodat, 1897) | | | | | | |
|  |  | ^6^Freshwater | - | Widened | Straight to transition region from opening. | Sometimes slightly curved, obliquely narrowed to pointed end | 84.0-118.0 | - | - |
|  |  | ^7^Freshwater | Internal wall of mother cell | Widened | Straight to transition region from opening | Obliquely narrowed to pointed end | 60.0-119.0 | 10.0-12.0 | - |
|  |  |  | 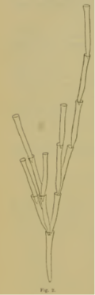 (Fig. 2 in Brunnthaler, 1901) | | | | | | |
|  |  | ^8^Freshwater | Internal wall of mother cell | Widened | Straight to transition region or slightly narrowed to then middle of upper part, then widened to transition region from opening | Obliquely narrowed to pointed apex from | 61.0-118.0 | 10.0-12.0 | - |
|  |  |  | 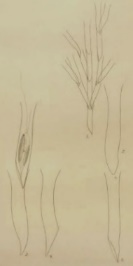 (Table XIX, Figs. 1-5 in Lemmermann, 1901a) | | | | | | |
|  |  | ^9^Freshwater | Internal wall of mother cell | Widened | Straight to transition region or slightly narrowed to then middle of upper part, then widened to transition region from opening | Obliquely narrowed to pointed apex from |  |  |  |
|  |  |  | 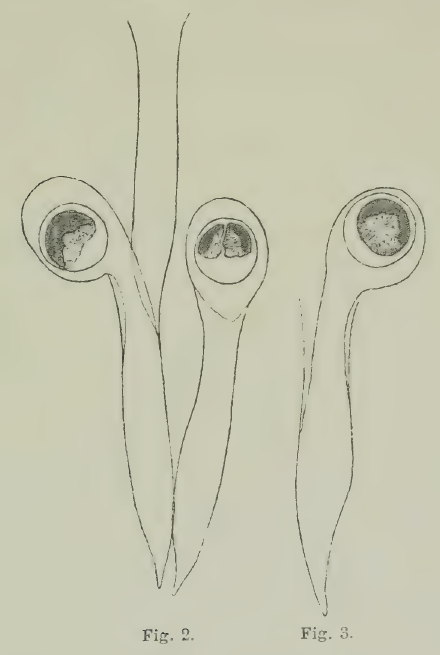 (Figs. 2-3 in Lemmermann, 1904) | | | | | | |
|  |  | ^11^Freshwater | Internal wall of mother cell | Widened | Straight to transition region or slightly narrowed to then middle of upper part, then widened to transition region from opening | Obliquely narrowed to pointed end | 61.0-118.0 | 10.0-12.0 | - |
|  |  |  | 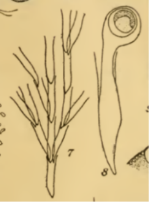 (Figs. 7-8 in Lemmermann, 1910) | | | | | | |
|  |  | ^13^Freshwater | Internal wall of mother cell | Widened | Straight to transition region from opening | Obliquely narrowed to pointed end | 57.0-72.0 | - | - |
|  |  |  | 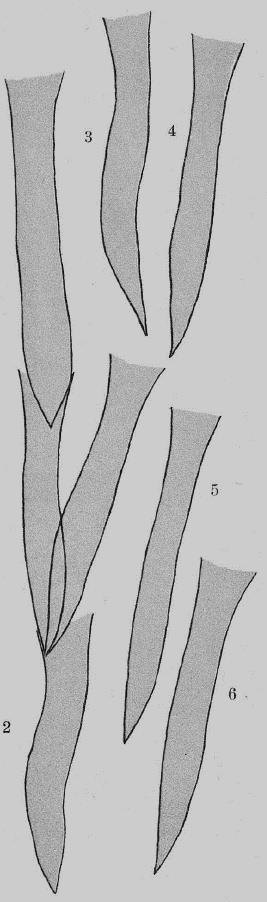 (Table XII, Figs. 2-6 in Bachmann, 1911) | | | | | | |
|  |  | ^14^Freshwater | Internal wall of mother cell | Widened | Straight to transition region or slightly narrowed to the middle of upper part, then widened to transition region from opening | Obliquely narrowed to pointed end | Up to 115 | - | - |
|  |  |  | 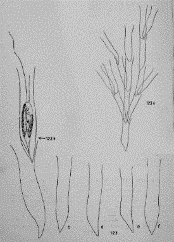 (Figs. 123a-f in Pashcer, 1913) | | | | | | |
|  |  | ^18^Freshwater | Internal wall of mother cell | Widened | Straight to transition region or slightly narrowed to the middle of upper part, then widened to transition region from opening | Obliquely narrowed to pointed end | 40-115 | - | - |
|  |  |  | 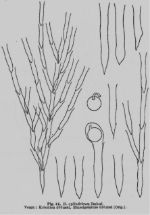 (Fig. 47 in Kreiger, 1930) | | | | | | |
|  |  | ^19^Freshwater | - | Widened flaring | Slightly narrowed to the middle of upper part, then widened to transition region from opening | Obliquely narrowed to blunt end | 30.0-77.0 | 8.5-12.5 | - |
|  |  |  | 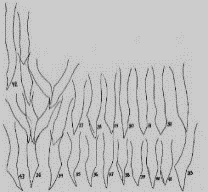 (Plate II, Figs. 26-43 in Ahlstrom, 1937) | | | | | | |
| *D. cylindricum*  var. *alpinum*^3,13,18^ |  | ^3^Freshwater | - | Widened | Slightly narrowed to the middle of upper part, then widened to transition region. | Obliquely narrowed to pointed end | 44.0-64.0 | 10.0 | - |
|  |  | ^13^Freshwater | Internal wall of mother cell | Widened | Slightly narrowed to the middle of upper part, then widened to transition region | Obliquely narrowed to pointed end | 44.0-64.0 | 10.0 | - |
|  |  |  | 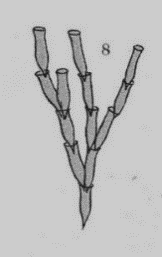 (Plate XII, Fig. 8 in Bachmann, 1911) | | | | | | |
|  |  | ^18^Freshwater | Internal wall of mother cell | Widened | Slightly narrowed to the middle of upper part, then widened to transition region. | Obliquely narrowed to pointed end | 40.0-64.0 | - | - |
|  |  |  | 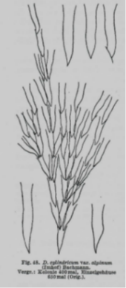 (Fig. 48 in Kreiger, 1930) | | | | | | |
| *D. cylindricum* var. *ceylonicum*^11^ |  | Freshwater | Internal wall of mother cell | Widened | Straight to transition region from opening, undulated (exceptionally strongly undulated at the transition region) | Slightly curved, obliquely narrowed, then attenuate to pointed end | 41.0-46.5 | 6.0-7.0 | 8.0-9.0 |
| *D. cylindricum* var. *holsaticum*^11,14^ |  | ^11^Freshwater | Internal wall of mother cell | Widened | Straight to transition region from opening | Slightly curved, obliquely narrowed to pointed end | 43.0 | 8.0 | 9.5 |
|  |  |  | 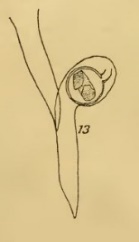 (Fig. 13 in Lemmermann, 1910) | | | | | | |
|  |  | ^14^Freshwater | Internal wall of mother cell | Widened | Straight to transition region from opening | Slightly curved, obliquely narrowed to pointed end | - | - | - |
|  |  |  | 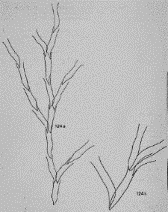 (Fig, 124a-b in Pascher, 1913) | | | | | | |
| *D. cylindricum* var. *kossogoinensis*^11^ |  | Freshwater | - | Widened flaring | Straight to transition region from opening | Narrowed to pointed end, one side was concave and opposite side was convex | 95.0-120.0 | 8.0-9.0 | 15.0 |
| *D. cylindricum* var. *palustre*^8,11,18,32,33^ |  | ^8^Freshwater | - | Widened | Straight to transition region from opening | Obliquely narrowed to pointed end | 49.0-68.0 | 8.0 | 11.0 |
|  |  |  | 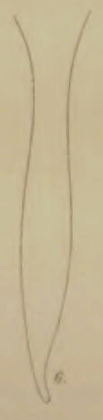 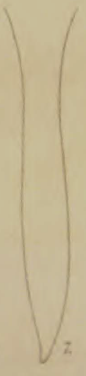 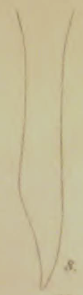 (Table XIX, Figs. 6-8 in Lemmermann, 1901a) | | | | | | |
|  |  | ^11^Freshwater | - | Widened | Straight to transition region from opening. | Obliquely narrowed to pointed end | 49.0-68.0 | 8.0 | 11.0 |
|  |  | ^18^Freshwater | Internal wall of mother cell | Widened | Straight to transition region or slightly narrowed to the middle of upper part, then widened to transition region | Sometimes curved, obliquely narrowed to pointed end | 45.0-100.0 | - | - |
|  |  |  | 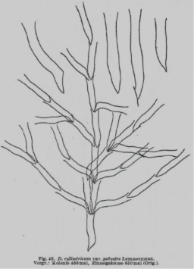 (Fig. 49 in Kreiger, 1930) | | | | | | |
|  | Bonghwa040718C (n=25) | ^32^Freshwater | Internal wall of mother cell | Widened | Slightly narrowed to the middle of upper part, then widened to undulated transition region | Curved, obliquely narrowed to pointed end, but present protuberances at both sides | 37.5-44.3 | 8.8-10.6 | 8.4-11.7 |
|  |  |  | 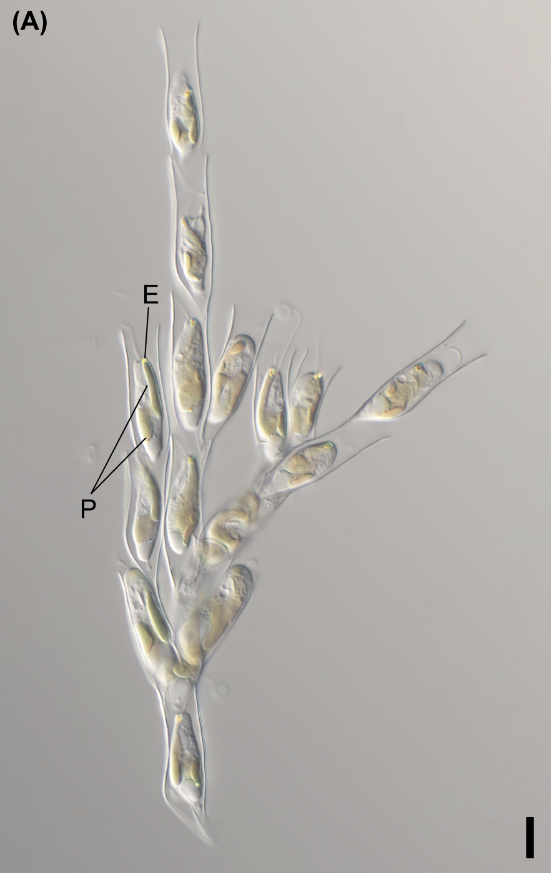 (Fig. 9A in this study. Scale bar = 10 µm) | | | | | | |
|  | (n=12) | ^33^Freshwater | Internal wall of mother cell | Widened | Slightly narrowed from opening to middle of upper part then widened to undulated transition region | Curved, obliquely narrowed to pointed end, but present protuberances at both sides | 50.0-67.3 | 7.1-10.0 | - |
|  |  |  | 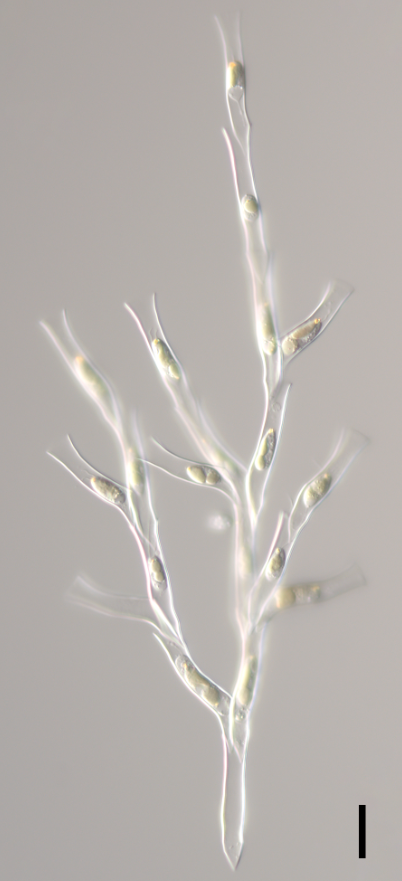 (Field sample in this study. Scale bar = 20 µm) | | | | | | |
| *D. divergens*^3,5,7,11,14,18,19^ |  | ^3^Freshwater | - | Widened | Straight to undulated transition region from opening | Attenuate to pointed end | 56.0 | - | - |
|  |  | ^5^Freshwater | Internal wall of mother cell | Widened | Slightly narrowed to the middle of upper part, then widened to undulated transition region | Attenuate to pointed end | 40.0-45.0 | 10.0-12.0 | - |
|  |  |  | 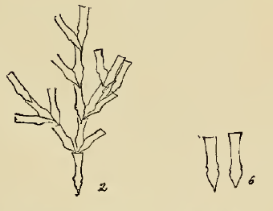 (Figs. 2, 6 in Chodat, 1897) | | | | | | |
|  |  | ^7^Freshwater | - | Widened | Straight to undulated transition region, slightly undulated | Attenuate to pointed end | 35.0-48.0 | 7.0-8.0 | - |
|  |  | ^8^Freshwater | Internal wall of mother cell | Widened | Slightly narrowed to the middle of upper part, then widened to undulated transition region | Curved, attenuate to pointed end | 35.0-47.0 | 7.0-8.0 | - |
|  |  |  | 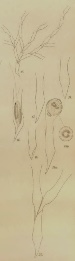 (Table XIX, Figs. 15-20 in Lemmermann, 1901a) | | | | | | |
|  |  | ^11^Freshwater | Internal wall of mother cell | Widened | Straight to transition region, undulated | Sometimes curved, attenuate to pointed end | 35.0-47.0 | 7.0-8.0 | - |
|  |  |  | 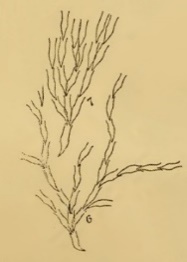 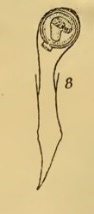 (Figs. 6-8 in Lemmermann, 1910) | | | | | | |
|  |  | ^14^Freshwater | Internal wall of mother cell | Widened | Straight to transition region, undulated | Curved, attenuate to pointed end | 35.0-50.0 | - | - |
|  |  |  | 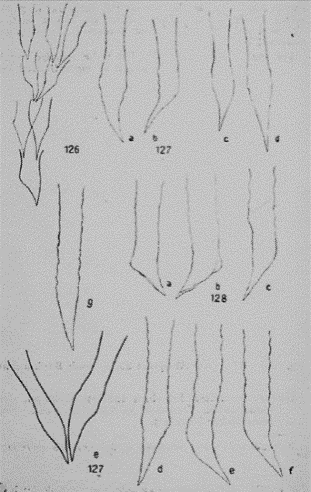 (Figs. 127a-d in Pashcer, 1913) | | | | | | |
|  |  | ^18^Freshwater | Internal wall of mother cell | Widened | Slightly narrowed to the middle of upper part, then widened to undulated transition region | Sometimes curved, attenuate to pointed end | 30.0-65.0 | 8.0-11.0 | - |
|  |  |  | 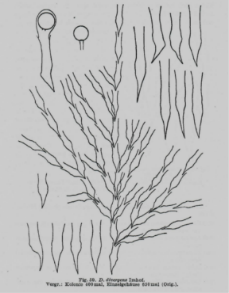 (Fig. 59 in Kreiger, 1930) | | | | | | |
|  |  | ^19^Freshwater | Internal wall of mother cell | Widened | Slightly narrowed to the middle of upper part, then widened to undulated transition region | Curved, attenuate to pointed end | 26.0-68.0 | - | - |
|  |  |  | 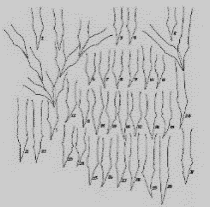 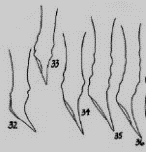 (Plate I, Figs. 1-36 in Ahlstrom, 1937) | | | | | | |
|  | CCMP2900 | ^32^Freshwater | Internal wall of mother cell | Widened | Slightly narrowed to the middle of upper part, then widened to transition region, undulated. | Sometimes curved, attenuate to pointed end | 35.4-43.2 | 8.6-9.8 | 6.1-9.8 |
|  |  |  | 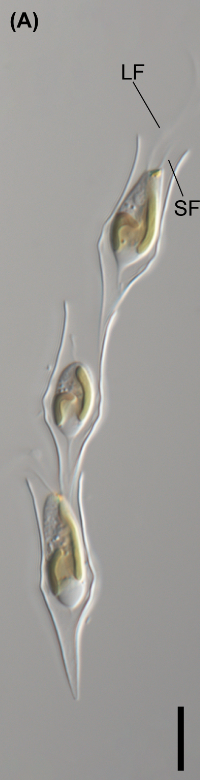 (Fig. 5A in this study. Scale bar = 10 µm) | | | | | | |
|  | CCMP3056 | ^32^Freshwater | Internal wall of mother cell | Widened | Slightly narrowed to the middle of upper part, then widened to transition region, undulated. | Sometimes curved, attenuate to pointed end | 31.6-39.4 | 7.8-8.7 | 6.1-8.5 |
|  |  |  | 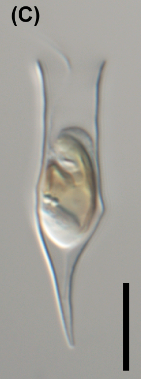 (Fig. 5C in this study. Scale bar = 10 µm) | | | | | | |
|  | Deokghi051818A (n=25) | ^32^Freshwater | Internal wall of mother cell | Widened | Straight to transition region or slightly narrowed to the middle of upper part, then widened to transition region, undulated | Sometimes slightly curved, attenuate to pointed end | 33.1-39.9 | 8.2-10.0 | 7.5-9.2 |
|  |  |  | 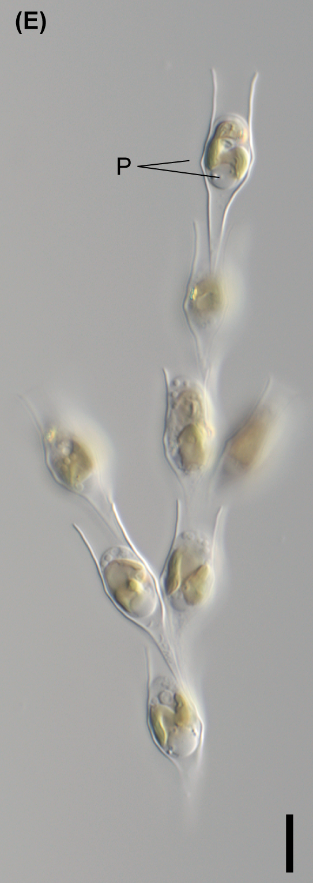 (Fig.. 5E in this study. Scale bar = 10 µm) |  |  |  |  |  |  |
|  | (n=18) | ^33^Freshwater | Internal wall of mother cell | Widened | Straight to transition region or slightly narrowed to the middle of upper part, then widened to transition region. | Sometimes slightly curved, attenuate to pointed end | 34.5-50.9 | 7.9-9.5 | - |
|  |  |  | 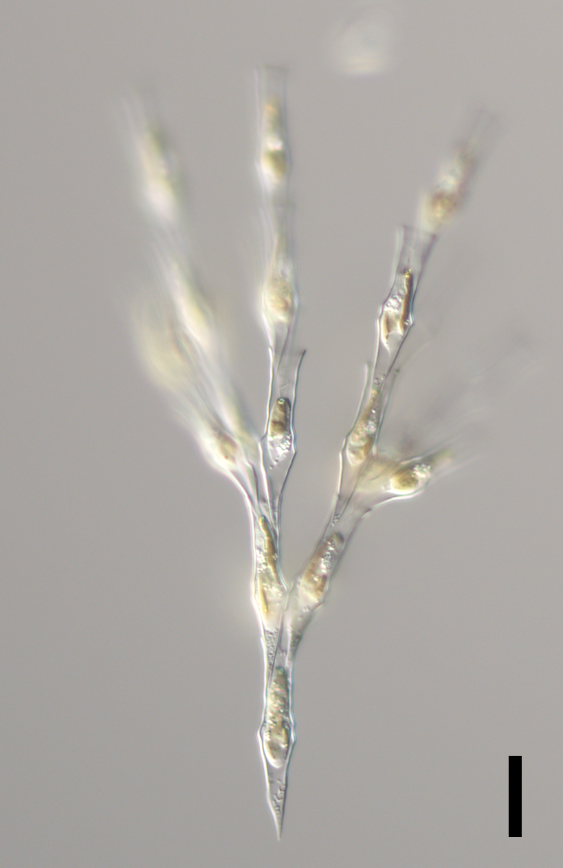 (Field sample in this study. Scale bar = 20 µm) | | | | | | |
| *D. divergens* var. *angulatum*^7,8,18^ |  | ^7^Freshwater | - | Widened | Straight to transition region, but not undulated | Sometimes slightly curved, attenuate to pointed end | 32.0 | - | - |
|  |  | ^8^Freshwater | Internal wall of mother cell | Widened | Slightly narrowed from opening to the middle of upper part, then widened to transition region | Attenuate to pointed end | - | - | - |
|  |  |  | 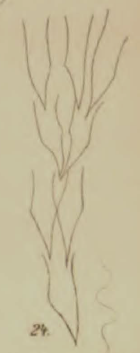 (Table XVIII, Fig. 24 in Lemmermann, 1901a) | | | | | | |
|  |  | ^18^Freshwater | Internal wall of mother cell | Widened | Slightly narrowed to the middle of upper part, then widened to transition region, sharp corner at the transition region | Sometimes slightly curved, attenuate to pointed end | 30.0-40.0 | - | - |
|  |  |  | 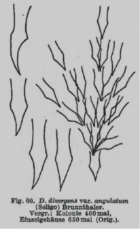 (Fig. 60 in Kreiger, 1930) | | | | | | |
| *D. divergens* var. *schauinslandii*^7,8,13,14,18^ |  | ^7^Freshwater | Internal wall of mother cell | Widened | Straight to transition region, undulated | Curved, attenuate to pointed end | 40.0-44.0 | 8.0 | 10.0-11.0 |
|  |  | ^8^Freshwater | Internal wall of mother cell | Widened | Straight to transition region | Slightly curved, attenuate to pointed end | 60.0-66.0 | 8.0 | 10.0-11.0 |
|  |  |  | 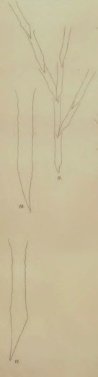 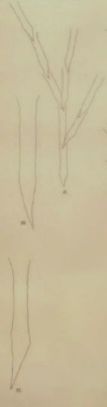 (Table XIX, Figs. 9-11 in Lemmermann, 1901a) | | | | | | |
|  |  | ^13^Freshwater | Internal wall of mother cell | - | Straight to transition region, undulated | Slightly curved, attenuate to pointed end | 64.0-65.0 | - | - |
|  |  |  | 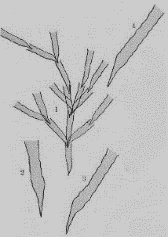 (Figs. 1-4 in Bachmann, 1911) | | | | | | |
|  |  | ^14^Freshwater | - | Straight or widened | Straight to transition region, undulated | Curved, attenuate to pointed end | 29.0-44.0 | - | - |
|  |  |  | 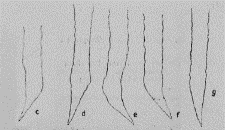 (Figs. 128c-g in Pascher, 1913) | | | | | | |
|  |  | ^18^Freshwater | - | Widened | Straight to transition region or widened at transition region, undulated | Curved, attenuate to pointed end | 35.0-65.0 | 8.0-9.0 | - |
|  |  |  | 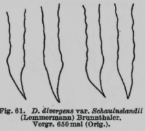 (Fig. 61 in Kreiger, 1930) | | | | | | |
| *D. exstoundulatum*^32,33^ | Dallae111421MS1 (n=25) | ^32^Freshwater | Internal wall of mother cell | Widened | Straight or slightly widened to transition region, undulated | Attenuate to pointed end | 33.2-45.2 | 7.4-9.4 | 6.2-8.3 |
|  |  |  | 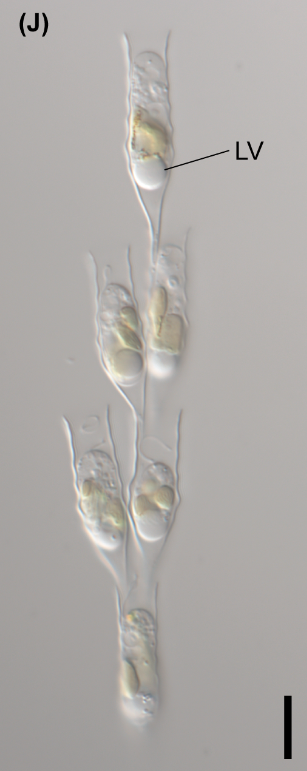 (Fig. 6J in this study. Scale bar = 10 µm) | | | | | | |
|  | (n=5) | ^33^Freshwater | Internal wall of mother cell | Widened | Straight or slightly widened to transition region from opening with undulation on lorica surface. | Attenuate to pointed tip from transition region | 42.5-54.9 | 6.4-7.3 | - |
|  |  |  | 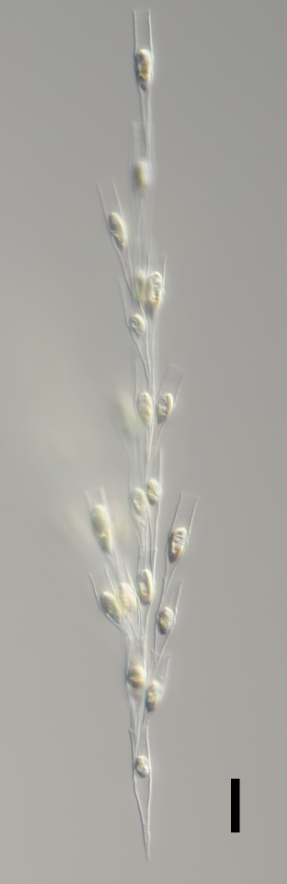 (Field sample in this study. Scale bar = 20 µm) | | | | | | |
| *D. inclinatum*^32,33^ | Chojeon011219A (n=25) | ^32^Freshwater | Internal wall of mother cell | Widened | Slightly narrowed to the middle of upper part, from opening, then widened to transition region, undulated | Inclined, obliquely narrowed to pointed end | 29.1-39.1 | 7.4-10.6 | 8.1-12.4 |
|  |  |  | 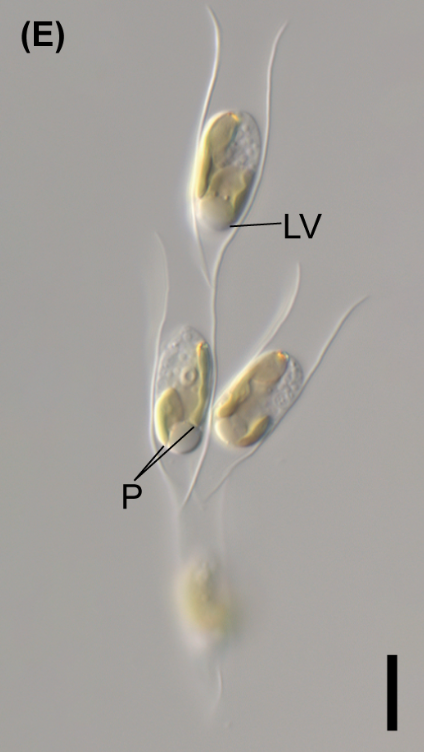 (Fig 7E in this study. Scale bar = 10 µm) | | | | | | |
|  | (n=7) | ^33^Freshwater | Internal wall of mother cell | Widened | Slightly narrowed to the middle of upper part, then widened to transition region. | Obliquely narrowed to pointed end | 41.0-49.7 | 8.6-9.3 | - |
|  |  |  | 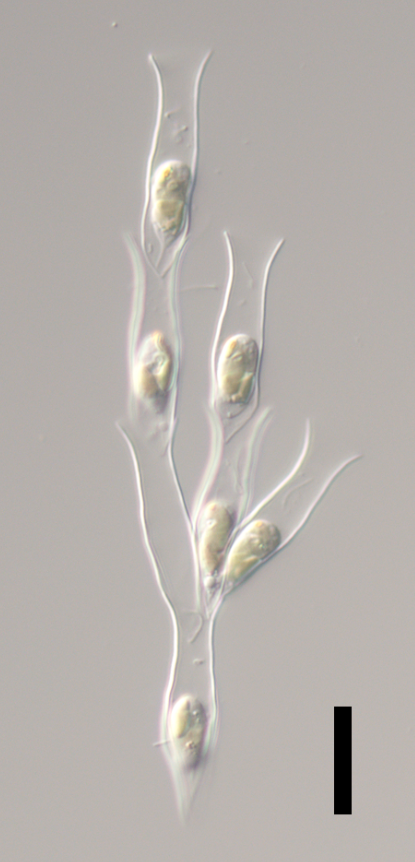 (Field sample in this study. Scale bar = 20 µm) | | | | | | |
| *D. lauzonicum*^19^ |  | Freshwater | - | Straight | Straight to transition region | Attenuate to swelling and pointed end from transition region | 69.0-100.0 | 10.0 | - |
|  |  |  | 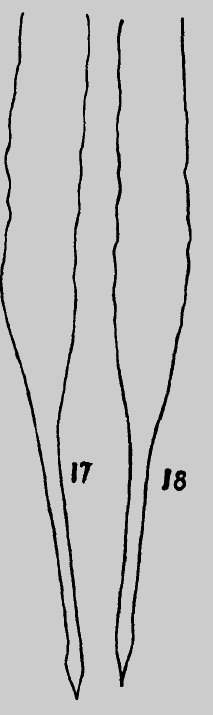 (Plate IV, Figs 17-18 in Ahlstrom, 1937) | | | | | | |
| *D. ningwuensis*^29,32,33^ |  | ^29^Freshwater | Internal wall of mother cell | Widened | Slightly narrowed to middle of upper part, then widened to transition region. | Obliquely narrowed to end | 23.0-36.0 | 8.0-12.0 | - |
|  |  |  |  (Figs. 2b-d in Jiang et al. 2019, scale bars = 20 μm (b), 10 μm (c-d)) | | | | | | |
|  | Myeoseul031718C (n=25) | ^32^Freshwater | Internal wall of mother cell | Widened | Straight to transition region or slightly narrowed to middle of upper part, then widened to transition region. | Obliquely narrowed to and one side was concave | 21.2-30.4 | 8.2-9.5 | 7.0-10.4 |
|  |  |  | 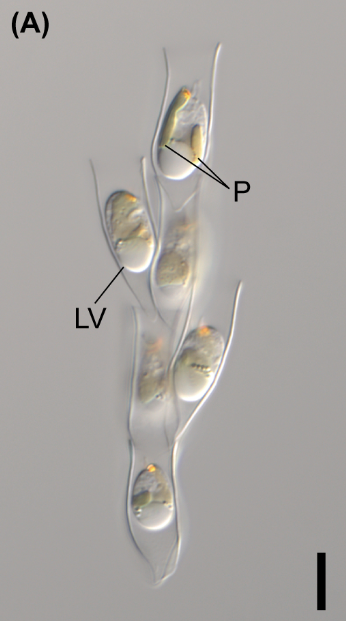 (Fig. 7A in this study. Scale bar = 10 µm) | | | | | | |
|  | (n=5) | ^33^Freshwater | Internal wall of mother cell | Widened | Straight to transition region or slightly narrowed to middle of upper part, then widened to transition region. | Obliquely narrowed to end | 23.2-32.4 | 7.0-8.6 | - |
|  |  |  | 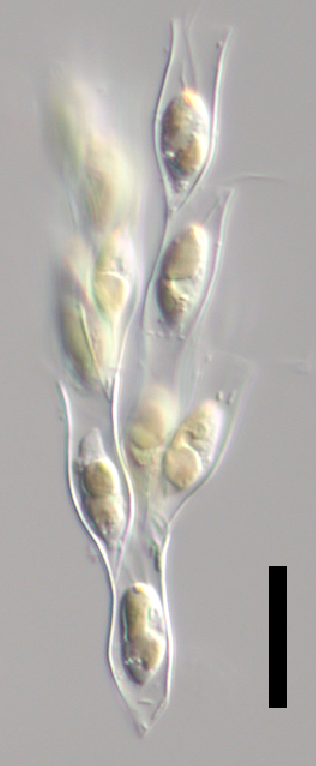 (Field sample in this study. Scale bar = 20 µm) | | | | | | |
| *D. pediforme*^7,8,11,14,15,18,19,31^ |  | ^7^Freshwater | - | Widened | Straight to transition region from opening, undulated, and present protuberance on one side at transition region | Obliquely attenuate to pointed end and one side is concave | 36.0-44.0 | 7.0 | - |
|  |  | ^8^Freshwater | Internal wall of mother cell | Widened | Straight to transition region, and present protuberance on one side at transition region | Curved, obliquely attenuate to pointed end and one side is concave | 36.0-44.0 | 7.0 | - |
|  |  |  | 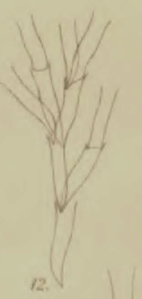 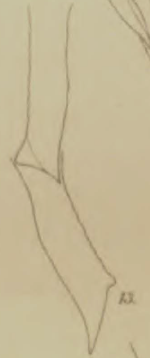 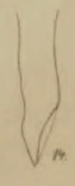 (Table XIX, Figs. 12-14 in Lemmermann, 1901a) | | | | | | |
|  |  | ^11^Freshwater | Internal wall of mother cell | Widened | Straight to transition region, and present protuberance on one side at transition region | Obliquely attenuate to pointed end and one side is concave | 36.0-44.0 | 7.0 | - |
|  |  |  | 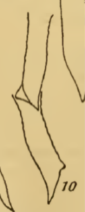 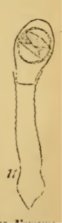 (Figs. 10-11 in Lemmermann, 1910) | | | | | | |
|  |  | ^14^Freshwater | - | Straight | Straight to transition region, undulated, and presnt protuberance on one side at transition region | Curved, obliquely attenuate to pointed end | - | - | - |
|  |  | ^15^Freshwater | - | Widened | Straight to transition region, and present protuberance on one side at transition region | Obliquely attenuate to pointed end | 29.0-35.0 | 7.0-9.0 | - |
|  |  |  | 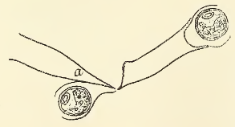 (Fig. 10a in Steinecke, 1915) | | | | | | |
|  |  | ^18^Freshwater | Internal wall of mother cell | Widened | Straight to transition region, sometimes undulated, and present protuberance on one side at transition region | Obliquely attenuate to pointed end and one side is concave | 35.0-45.0 | 8.0-9.0 | - |
|  |  |  | 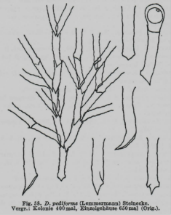 (Fig. 58 in Kreiger, 1930) | | | | | | |
|  |  | ^19^Freshwater | Internal wall of mother cell | Widened | Straight to transition region, sometimes undulated, and present protuberance on one side or both side at transition region | Obliquely attenuate to pointed end and one side is concave | 24.0-48.0 | - | - |
|  |  |  | 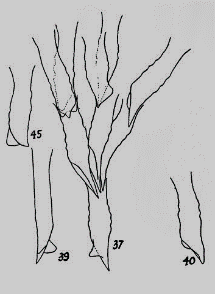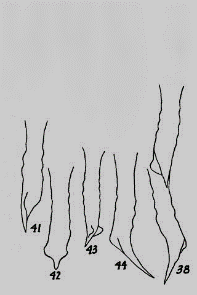 (Plate I, Figs. 37-45 in Ahlstrom, 1937) | | | | | | |
|  |  | ^31^Freshwater | Internal wall of mother cell | Sometimes widened | Straight to transition region, sometimes undulated, and present protuberance on one side or both side at transition region | Obliquely attenuate to pointed end and one side is concave | 27.1-49.0 | 5.0-8.3 | 7.1-11.5 |
|  |  |  |  (Figs 2a,c,e in Piatek et al., 2020) | | | | | | |
| *D. porrectum*^17,18^ |  | ^17^Marine | Internal wall of mother cell | Widened | Obliquely narrowed to transition region from opening | One side is sloping while opposite side is straight to pointed end. | 26.0-40.0 | 6.0-7.0 | - |
|  |  |  | 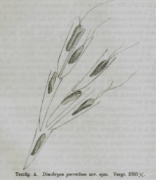 (Fig. A in Schiller, 1925) | | | | | | |
|  |  | ^18^Marine | Internal wall of mother cell | Widened | Obliquely narrowed to transition region from opening | One side is sloping while opposite side is straight to pointed end. | - | - | - |
|  |  |  | 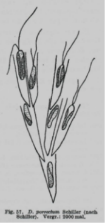 (Fig. 57 in Kreiger, 1930) | | | | | | |
| *D. praecambrianum*^25^ |  | Freshwater | Internal wall of mother cell | Widened flaring | Straight to transition region or slightly narrowed to the middle of upper part, then widened to transition region, undulated | Curved, attenuate to pointed end | 26.0-37.0 | - | 10.0-15.0 |
|  |  |  |   (Figs. 5-6, 15-16 in Nicholls, 2000) | | | | | | |
| *D. sertularia*^1,7,8,11,13,14,18,27^ |  | ^1^Freshwater | Internal wall of mother cell | Widened | Slightly narrowed to the middle of upper part, then widened to transition region. | Obliquely narrowed to end | 52.9 | - | - |
|  |  |  | 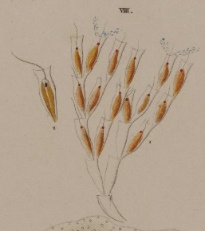 (Plate VIII, Figs. VIII1-2 in Ehrenberg, 1838) | | | | | | |
|  |  | ^7^Freshwater | Internal wall of mother cell | Widened | Slightly narrowed to the middle of upper part, then widened to transition region. | Obliquely narrowed to end | up to 47.0 | 13.0 | 10.0-11.0 |
|  |  | ^8^Freshwater | Internal wall of mother cell | Widened | Slightly narrowed to the middle of upper part, then widened to transition region. | Obliquely narrowed to end | 44.0 | 13.0 | 13.0 |
|  |  |  | 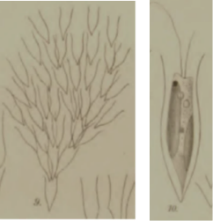 (Table XVIII, Figs. 9-10 in Lemmermann, 1901a) | | | | | | |
|  |  | ^11^Freshwater | Internal wall of mother cell | Widened | Slightly narrowed to middle of upper part, then widened to transition region. | Obliquely narrowed to pointed end | 30.0-44.0 | 10.0-13.0 | - |
|  |  |  | 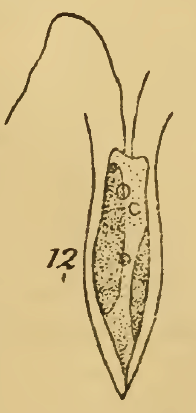 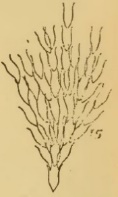 (Figs. 12,15 in Lemmermann, 1910) | | | | | | |
|  |  | ^13^Freshwater | Internal wall of mother cell | Widened | Slightly narrowed to middle of upper part, then widened to transition region | Obliquely narrowed to pointed end | 27.0-50.0 | Up to 13.0 | - |
|  |  |  | 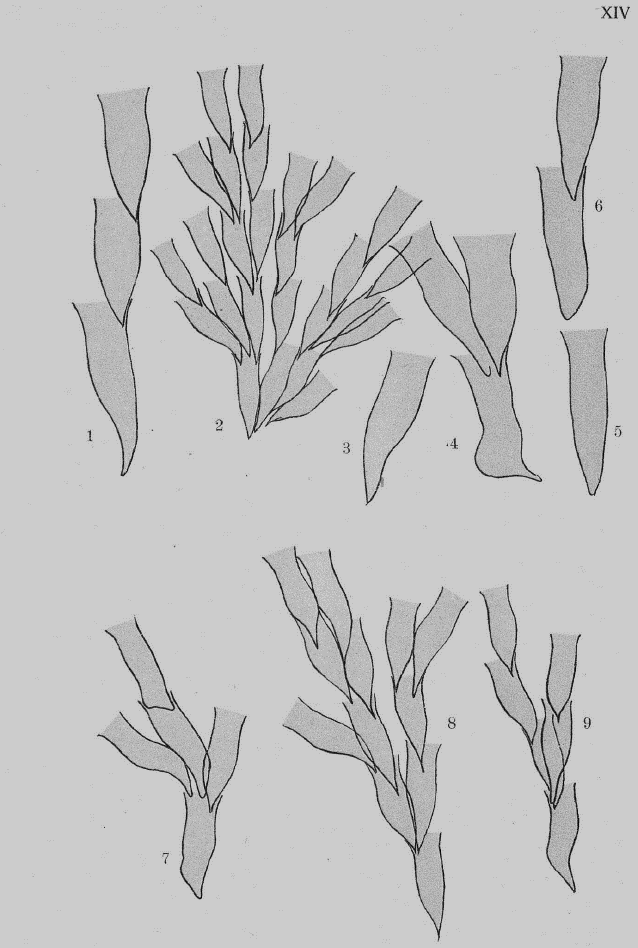 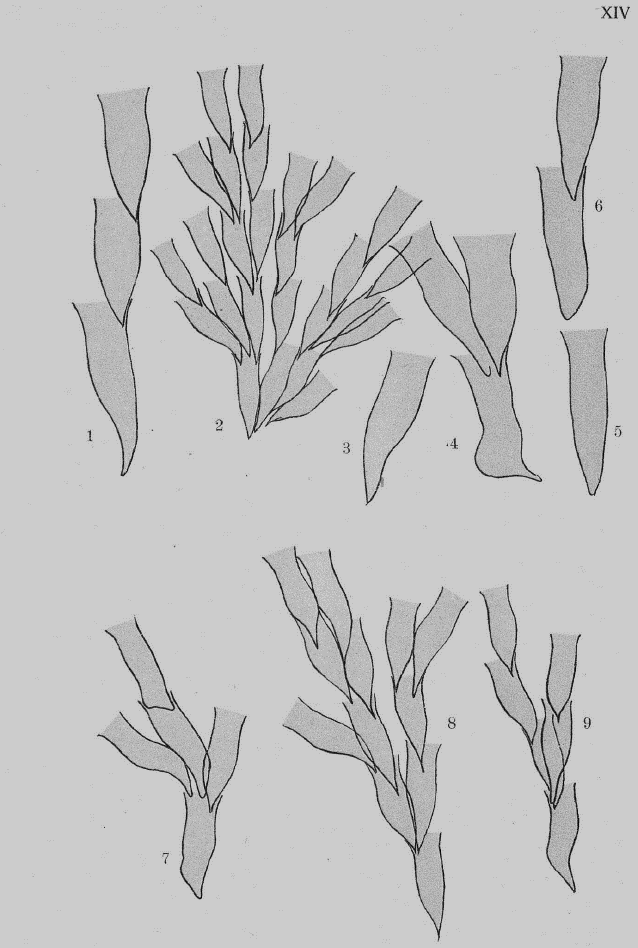 (Plate XIV, Figs. 1-9 in Bachmann, 1910) | | | | | | |
|  |  | ^14^Freshwater | Internal wall of mother cell | Widened | Slightly narrowed to middle of upper part, then widened to transition region | Obliquely narrowed to pointed end | 30.0-44.0 | 10.0-14.0 | - |
|  |  |  | 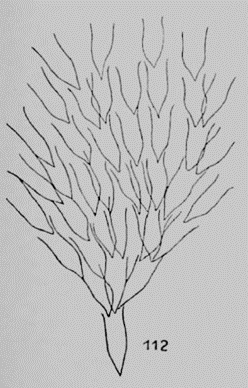 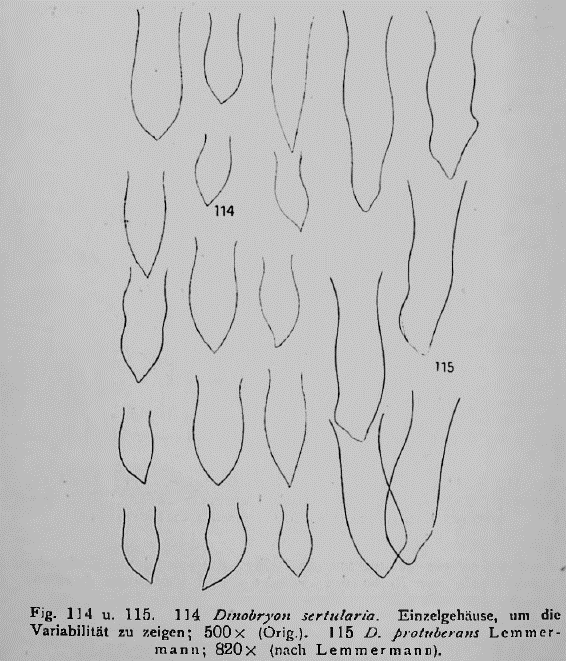 (Fig. 112, 114 in Pashcer, 1913) | | | | | | |
|  |  | ^18^Freshwater | Internal wall of mother cell | Widened | Slightly narrowed to middle of upper part, then widened to transition region | Obliquely narrowed to end | 30.0-40.0 | 10.0-12.0 | - |
|  |  |  | 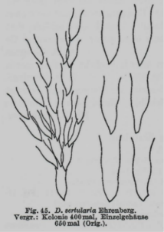 (Fig. 45 in Kreiger, 1930) | | | | | | |
|  |  | ^19^Freshwater | Internal wall of mother cell | Widened flaring | Slightly narrowed to middle of upper part, then widened to transition region. | Sometimes curved, obliquely narrowed to blunt end | 23.0-43.0 | - | - |
|  |  |  | 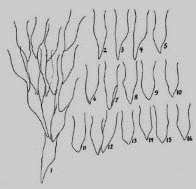 (Plate II, Figs. 1-16 in Ahlstrom, 1937) | | | | | | |
|  |  | ^27^Freshwater | Internal wall of mother cell | Widened | Slightly narrowed to middle of upper part, then widened to transition region. | Obliquely narrowed to end | 27.5-43.7 | 8.0-11.2 | - |
|  |  |  |    (Figs. 1-7 in Piatek et al., 2012, scale bars = 10 μm) | | | | | | |
| *D. sertularia* var. *protuberans*^8,11,14,18^ |  | ^8^Freshwater | Internal wall of mother cell | Widened | Slightly narrowed to middle of upper part, then widened to transition region. | Obliquely narrowed to pointed end with irregular protuberances. | 37.0-40.0 | 7.0-10.0 | 10.0-11.0 |
|  |  |  | 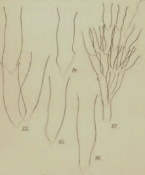 (Table XVIII, Figs. 12-16 in Lemmermann, 1901a) | | | | | | |
|  |  | ^11^Freshwater | Internal wall of mother cell | Widened | Slightly narrowed to middle of upper part, then widened to transition region. | Obliquely narrowed to pointed end with protuberances. | 37.0-40.0 | 7.0 | 10.0-11.0 |
|  |  |  | 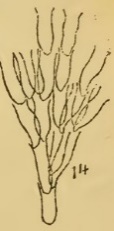 (Fig. 14 in Lemmermann, 1910) | | | | | | |
|  |  | ^14^Freshwater | Internal wall of mother cell | Widened | Slightly narrowed to middle of upper part, then widened to transition region. | Obliquely narrowed to pointed end with irregular protuberances. | 35.0-40.0 | 7.0-10.0 | - |
|  |  |  | 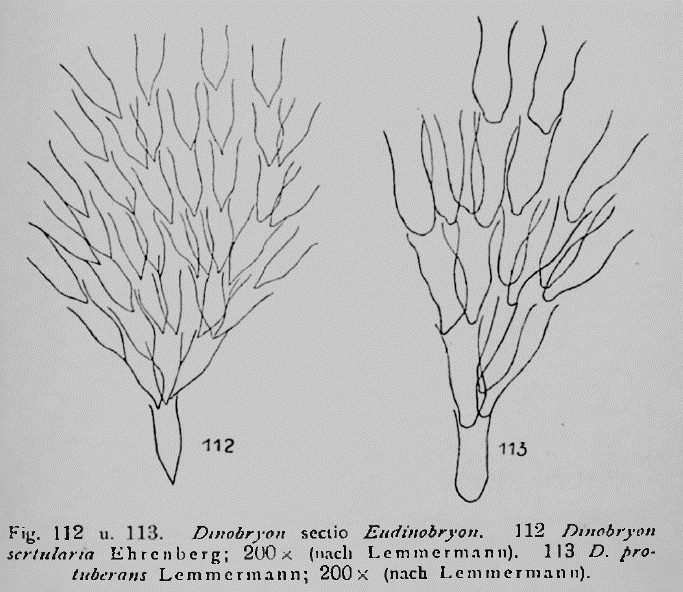 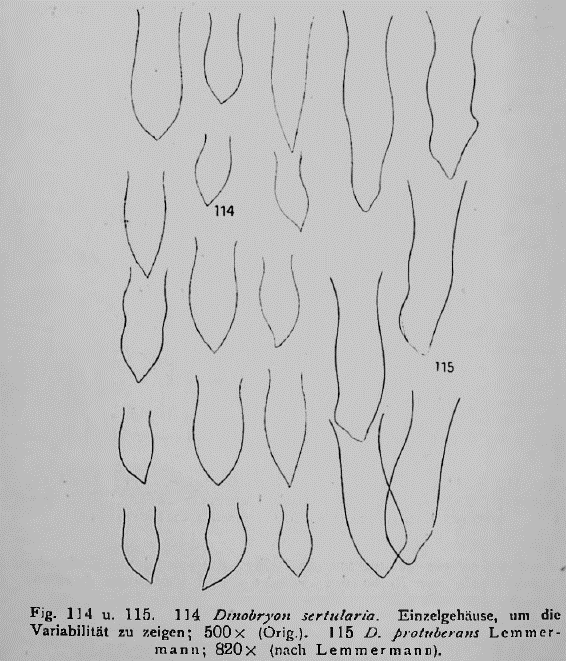 (Figs. 113, 115 in Pashcer, 1913) | | | | | | |
|  |  | ^18^Freshwater | Internal wall of mother cell | Widened | Slightly narrowed to middle of upper part, then widened to transition region. | Obliquely narrowed to pointed end with irregular protuberances. | 30.0-40.0 | 10.0-12.0 | - |
|  |  |  | 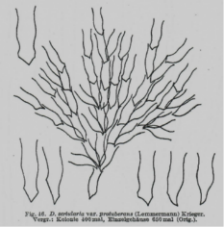 (Fig. 46 in Kreiger, 1930) | | | | | | |
| *D. sertularia* var. *thyrsoideum*^5,8,32,33^ |  | ^5^Freshwater | Internal wall of mother cell | Widened | Slightly narrowed to middle of upper part, then widened to transition region | Obliquely narrowed to end | 30.0-40.0 | 10.0-12.0 | - |
|  |  |  | 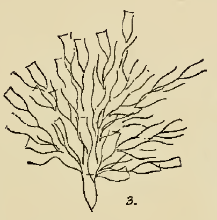 (Fig. 3 in Chodat, 1897) | | | | | | |
|  |  | ^8^Freshwater | - | Widened | Slightly narrowed to middle of upper part, then widened to transition region. | Obliquely narrowed to pointed end | 30.0-40.0 | 10.0-12.0 | - |
|  |  |  | 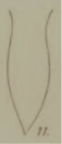 (Table XVIII, Fig. 11 in Lemmermann, 1901a) | | | | | | |
|  | Chosan041710C (n=25) | ^32^Freshwater | Internal wall of mother cell | Widened | Slightly narrowed to middle of upper part, then widened to transition region, undulated | Obliquely narrowed to pointed end, and rarely present protuberance at transition region | 24.4-33.2 | 7.5-9.0 | 7.7-9.7 |
|  |  |  | (Fig. 8F in this study. Scale bar = 10 µm) | | | | | | |
|  |  | ^33^Freshwater | Internal wall of mother cell | Widened | Slightly narrowed to middle of upper part, then widened to transition region, undulated | Obliquely narrowed to pointed end, and rarely present protuberance at transition region | 28.8-38.7 | 7.9-9.7 |  |
|  |  |  | (Field sample in this study. Scale bar = 20 µm) | | | | | | |
| *D. sertularia* var. *vindobonensis*^20^ |  | Freshwater | Internal wall of mother cell | Widened | Slightly narrowed to middle of upper part, then widened to transition region. | Obliquely narrowed to pointed end | 25.0-35.0 | 8.0-10.0 | - |
|  |  |  | (Fig. 2a in Mack, 1951) | | | | | | |
| *D. sertularoides*^28^ |  | Freshwater | Internal wall of mother cell | Widened flaring | Slightly narrowed to middle of upper part, then widened to transition region, and conspicuous protuberance present at one side of transition region. | Attenuate to pointed end | 22.0-26.0 | 11.0-16.0 | 9.0-11.0 |
|  |  |  | (Figs 2A-B in Hall & Karl, 2016) | | | | | | |
| *D. sociale*^1,7,8,11,13,14,18,19,32,33^ |  | ^1^Freshwater | Internal wall of mother cell | Obliquely narrowed to pointed apex from opening; conical shape. | | | 35.3 | - | - |
|  |  |  | (Plate VIII, Fig. IX in Ehrenberg, 1838) | | | | | | |
|  |  | ^7^Freshwater | - | Obliquely narrowed to pointed apex from widened opening; conical shape. | | | 32.0 | 7.0-8.0 | - |
|  |  | ^8^Freshwater | Internal wall of mother cell | Obliquely narrowed to pointed apex from widened opening; conical shape. | | | 34.0-45.0 | - | 7.0-8.0 |
|  |  |  | (Table XVIII, Figs. 17-18 in Lemmermann, 1901a) | | | | | | |
|  |  | ^11^Freshwater | - | Obliquely narrowed to pointed apex from widened opening; conical shape. | | | 30.0-68.0 | - | 7.0-8.0 |
|  |  |  | (Figs. 2-5 in Lemmermann, 1910) | | | | | | |
|  |  | ^13^Freshwater | Internal wall of mother cell | Widened | Slightly narrowed to middle of upper part, then widened to transition region | Attenuate to pointed end | 40.5-58.5 | - | - |
|  |  |  | (Plate XI, Figs. 1-9, 17-27 in Bachmann, 1911) | | | | | | |
|  |  | ^14^Freshwater | Obliquely narrowed to pointed apex from widened opening; conical shape, sometimes undulated | | | | 30.0-41.0 | 7.0-8.0 | - |
|  |  |  | (Figs. 116a-c in Pashcer, 1913) | | | | | | |
|  |  | ^18^Freshwater | Obliquely narrowed to pointed apex from straight or widened opening; conical shape, sometime one third of lower part is curved. | | | | 30.0-50.0 | 8.0-10.0 | - |
|  |  |  | (Fig. 54 in Kreiger, 1930) | | | | | | |
|  |  | ^19^Freshwater | - | Widened | Slightly narrowed to middle of upper part, then widened to transition region, rarely undulated | Attenuate to pointed end | 28.0-76.0 | - | - |
|  |  |  | (Plate III, Figs. 37-52 in Ahlstrom, 1937) | | | | | | |
|  | Angol061922MS3 (n=25) | ^32^Freshwater | Internal wall of mother cell | Widened | Slightly narrowed to middle of upper part, then widened to transition region | Attenuate to pointed end | 28.8-37.1 | 7.2-8.6 | 7.8-10.4 |
|  |  |  | (Fig. 6D in this study. Scale bar = 10 µm) | | | | | | |
|  | (n=5) | ^33^Freshwater | Internal wall of mother cell | Widened | Slightly narrowed to middle of upper part, then widened to transition region | Attenuate to pointed end | 29.3-31.2 | 7.0-8.6 | - |
|  |  |  | (Field smaple in this study. Scale bar = 20 µm) | | | | | | |
| *D. sociale* var. *americanum*^7,13,11,18,26^ |  | ^7^Freshwater | - | Widened | Slightly narrowed to middle of upper part, then widened to transition region | Attenuate to pointed end | 30.0-39.0 | 9.0-10.0 | - |
|  |  |  | (Fig. 3 in Brunnthaler, 1901) | | | | | | |
|  |  | ^11^Freshwater | - | Widened | Straight to transition region without undulation on lorica surface | Attenuate to pointed end | 30.0-39.0 | 9.0-10.0 | - |
|  |  | ^13^Freshwater | Internal wall of mother cell | Widened | Slightly narrowed to middle of upper part, then widened to transition region | Attenuate to pointed end | 34.5-37.5 | - | - |
|  |  |  | (Plate XI, Figs. 10-13, 28-31, 35-37 in Bachmann, 1911) | | | | | | |
|  |  | ^14^Freshwater | - | Widened | Slightly narrowed to middle of upper part, then widened to transition region | Obliquely narrowed to pointed end | - | - | - |
|  |  |  | (Fig. 117 in Pashcer, 1913) | | | | | | |
|  |  | ^18^Freshwater | Internal wall of mother cell | Widened | Slightly narrowed to middle of upper part, then widened to transition region | Attenuate to pointed end | 25.0-40.0 | 8.0-10.0 | - |
|  |  |  | (Fig. 56 in Kreiger, 1930) | | | | | | |
|  |  | ^19^Freshwater | Internal wall of mother cell | Widened | Slightly narrowed to middle of upper part, then widened to transition region | Attenuate to pointed end | 21.0-40.0 | - | - |
|  |  |  | (Plate III, Figs. 30-36 in Ahlstrom, 1937) | | | | | | |
|  |  | ^26^Freshwater | Internal wall of mother cell | Widened | Slightly narrowed to middle of upper part, then widened to transition region | Attenuate to pointed end | 24.0-40.0 | - | 7.1-9.6 |
|  |  |  | (Figs. 1-11 in Piatek, 2008, scale bars = 10 μm) | | | | | | |
| *D. sociale* var. *elongatum*^3,8,13^ |  | ^3^Freshwater | - | Widened | Narrowed to middle of upper part, then widened to transition region | Attenuate to pointed end | 56.0-92.0 | - | - |
|  |  | ^5^Freshwater | Internal wall of mother cell | Obliquely narrowed to pointed apex from opening; conical shape. | | | 56.0-98.0 | 5.0-9.0 | - |
|  |  |  | (Fig. 5 in Brunnthaler, 1901) | | | | | | |
|  |  | ^8^Freshwater | Internal wall of mother cell | Straight or slightly widened | Straight to transition region | Attenuate to pointed end | 56.0-96.0 | 5.0-9.0 | - |
|  |  |  | (Table XVIII, Fig. 20 in Lemmermann, 1901a) | | | | | | |
|  |  | ^11^Freshwater | - | Obliquely narrowed to pointed apex from opening without undulation on lorica surface; conical shape. | | | 56.0-92.0 | 5.0-9.0 | - |
|  |  | ^13^Freshwater | Internal wall of mother cell | Obliquely narrowed to pointed apex from opening without undulation on lorica surface; conical shape. | | | 56.0-92.0 | 5.0-9.0 | - |
| *D. sociale* var. *medium*^11^ |  | Freshwater | - | Obliquely narrowed to pointed apex from opening; conical shape. | | | 34.0-71.0 | 7.0 | 8.0 |
| *D. sociale* var. *stipitatum*^2,11,13,18^ |  | ^2^Freshwater | Internal wall of mother cell | Widened | Narrowed to middle of upper part, then widened to transition region | Attenuate to a long with a sharply pointed end | - | - | - |
|  |  |  | (Table XII, Fig. 5 in Stein, 1878) | | | | | | |
|  |  | ^11^Freshwater | - | Obliquely narrowed to pointed apex from opening; conical shape, slightly narrowed below the opening | | | 56.0-96.0 | - | - |
|  |  | ^13^Freshwater | Internal wall of mother cell | Widened | Narrowed to middle of upper part, then widened to transition region | Attenuate to a long with a sharply pointed end | 60.0 | - | - |
|  |  |  | (Plate XI, Figs 14-16 in Bachmann, 1911) | | | | | | |
|  |  | ^18^Freshwater | Internal wall of mother cell | Obliquely narrowed to pointed apex from opening; conical shape, sometimes one half of lower part is curved | | | 35.0-70.0 | 7.0-9.0 | - |
|  |  |  | (Fig. 55 in Kreiger, 1930) | | | | | | |
| *D. similis*^32,33^ | Sanseong2ji102318A (n=25) | ^32^Freshwater | Internal wall of mother cell | Widened | Straight or slightly widened to transition region, undulated | Attenuate to pointed end | 42.0-53.8 | 6.6-8.8 | 5.8-8.2 |
|  |  |  | (Fig 6G in this study. Scale bar = 10 µm) | | | | | | |
|  | (n=8) | ^33^Freshwater | Internal wall of mother cell | Widened | Straight or slightly widened to transition region, undulated | Attenuate to pointed end | 48.5-66.8 | 6.7-8.9 | - |
|  |  |  | (Field sample in this study. Scale bar = 20 µm) | | | | | | |
| *)D. spinum*^32^ | Geumgok020610D (n=25) | Freshwater | Internal wall of mother cell | Widened | Slightly narrowed to middle of upper part, then widened to transition region, undulated | Obliquely narrowed to pointed or flattened end, unusual presence of protuberance. | 22.0-29.1 | 6.9-9.0 | 6.9-10.0 |
| *D. taiyuanensis*^30,32,33^ |  | ^30^Freshwater | Internal wall of mother cell | Widened | Slightly narrowed to middle of upper part, then widened to transition region. | Obliquely narrowed to pointed or flattened end with protuberance. | 20-28 | 6.5-8.0 | - |
|  |  |  | (Figure 2a-b in Jiang et al. 2019, , scale bars = 20 µm (a), 10 µm (b)) | | | | | | |
|  | Yookhoje031321MS4 (n=25) | ^32^Freshwater | Internal wall of mother cell | Widened | Slightly narrowed to middle of upper part, then widened to transition region. | Obliquely narrowed to pointed or flattened end, present protuberance at both sides. | 26.2-33.2 | 7.3-8.7 | 7.3-9.8 |
|  |  |  | (Fig. 7C in this study. Scale bar = 10 µm) | | | | | | |
|  | (n=17) | ^33^Freshwater | Internal wall of mother cell | Widened | Slightly narrowed to middle of upper part, then widened to transition region | Obliquely narrowed to pointed or flattened end | 31.6-38.5 | 7.0-8.4 | - |
|  |  |  | (Field sample in this study. Scale bar = 20 µm) | | | | | | |
| *D. ungeuntariforme*^23^ |  | Freshwater | Internal wall of mother cell | Narrowed | Obliquely widened to transition region from opening. | Obliquely narrowed to apex from transition region. | 53.0-60.0 | 7.0-9.0 | 2.0-3.0 |
|  |  |  | (Figs. 1-6 in Croome et al. 1988, scale bars = 10 μm) | | | | | | |

1. Ehrenberg, 1838, 2. Stein, 1878, 3. Imhof 1887, 4. Imhof 1890, 5. Chodat, 1897, 6. Bachmann, 1901, 7. Brunnthaler, 1901, 8. Lemmermann, 1901a, 9. Lemmermann, 1904, 10. West & West, 1909, 11. Lemmerman, 1910, 12. Meunier, 1910, 13. Bachmann, 1911, 14. Pascher, 1913 15. Steinecke, 1915, 16. Bachmann, 1921 17. Schiller, 1925, 18. Kreiger, 1930, 19. Ahlstrom, 1937, 20. Mack, 1951, 21. Asmund, 1955a, 22. Hillard & Asmund, 1963, 23. Croome et al. 1988, 24. Throdsen, 1997, 25.Nicholls, 2000, 26. Piatek et al. 2008, 27. Piatek et al. 2012, 28. Hall & Karol, 2016, 29. Jiang et al. 2019a, 30. Jiang et al. 2019b, 31. Piatek at al. 2020, 32. Culture sample, 33. Field sample.
