# Supplementary material for: Multigene phylogeny reveals a cryptic diversity in the genus Dinobryon (Chrysophyceae) with integrative description of five new species
Source: Front Plant Sci. 2023 Apr 18;14:1150814. doi: 10.3389/fpls.2023.1150814 (PMC10151809; doi:10.3389/fpls.2023.1150814)
Supplement: Supplementary file 6 [file Table_4.docx]

**Supplementary Table 4.** Summary of stomatocyst morphological characteristics of colonial *Dinobryon* species. (-) denotes unrecorded information in literatures.

| Taxon | Strain | **Stomatocyst morphology** | | | | | | | | | | | | |
| --- | --- | --- | --- | --- | --- | --- | --- | --- | --- | --- | --- | --- | --- | --- |
|  |  | Cyst body | Length of cyst body (µm) | Width of cyst body (µm) | | | Collar | | Height of collar (µm) | | Diameter of collar (µm) | Pore | Stomatocyst number | |
| *D. annulatum*^10^ |  | Oval to round | 10-12 in diameter | | | | | Slightly raised | - | | - | - | | - |
|  |  | 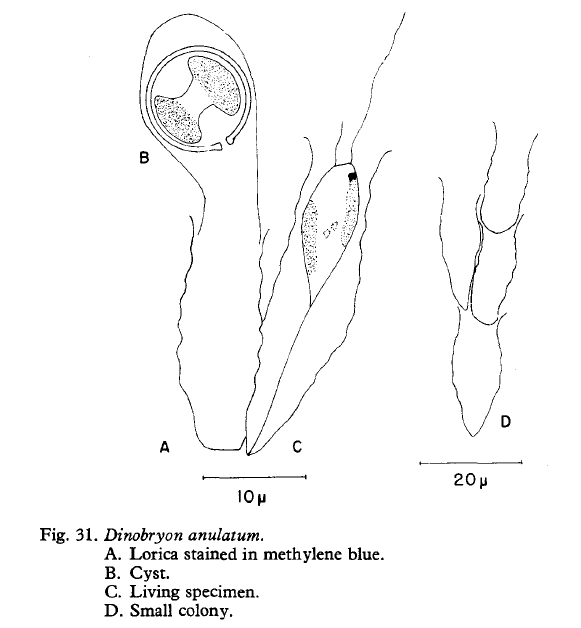 (Figs. 31B in Hillard & Asmund, 1963) | | | | | | | | | | | | |
| *D. bavaricum*^5,7^ |  | ^5^Spherical | 8-12 in diameter | | | | | Obconical | - | | - | - | - | |
|  |  | 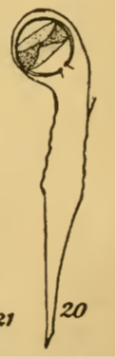 (Fig. 20 in Lemmermann, 1910) | | | | | | | | | | | | |
|  |  | ^7^Spherical | 11 in diameter | | | | | Cylindrical | - | | - | - | - | |
|  |  | 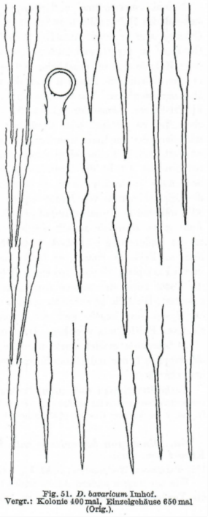 (Fig. 51 in Kreiger, 1930) | | | | | | | | | | | | |
| *D. crenulatum*^9^ |  | Spherical | 10 in diameter | | | | | Obconical | 3-5 | | - | - | - | |
|  |  | 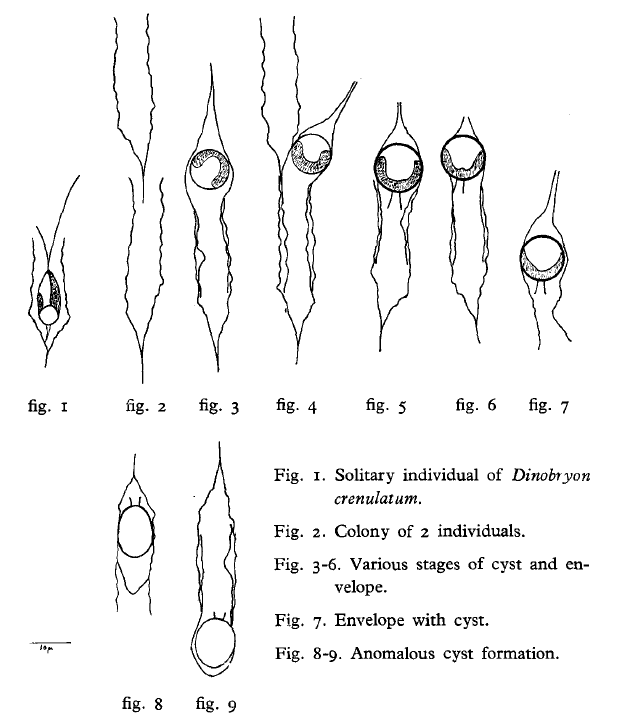 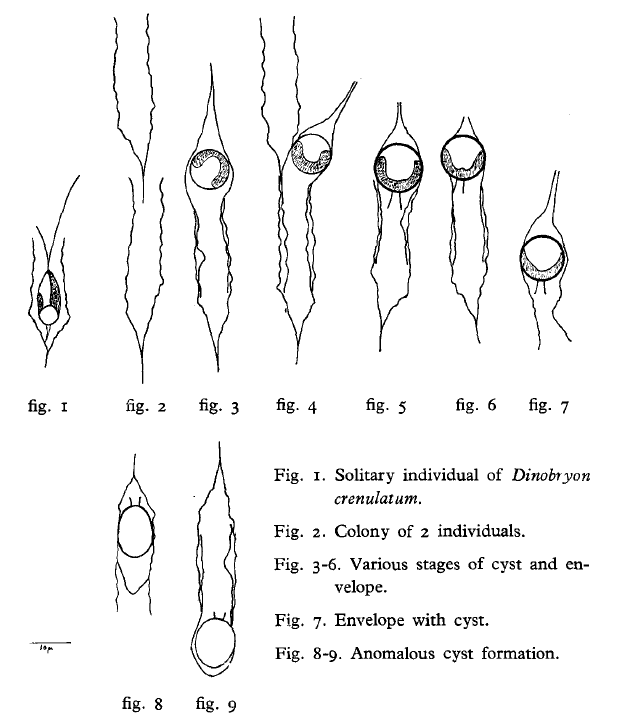 (Figs. 1-9 in Asmund, 1955a) | | | | | | | | | | | | |
| *D. cylindricollarium*^21^ | Myeoseul111618D | Spherical with smooth surface | 9.8-12.8 | 9.8-13.0 | | | | Flat planar annulus | 1.5-3.3 | | 2.3-3.1 | Regular | - | |
|  |  | 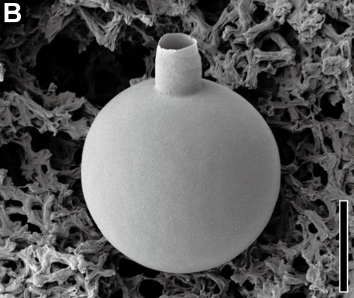 (Fig. 10B in this study. Scale bar = 5 µm) | | | | | | | | | | | | |
| *D. cylindricum*^4,7,13,16^ |  | ^4^Spherical with dotted surface | 12 in diameter | | | | | Absence | - | | - | - | - | |
|  |  | 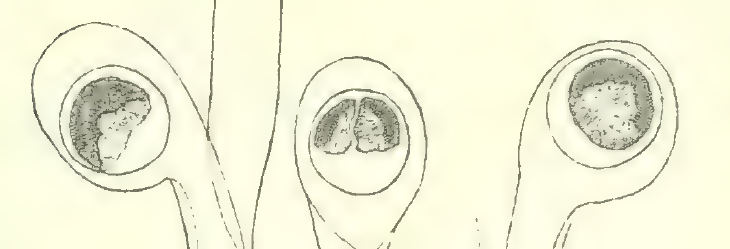 (Figs. 2,3 in Lemmermann, 1904) | | | | | | | | | | | | |
|  |  | ^7^Spherical | 12-14 in diameter | | | | | Hooked | - | | - | - | - | |
|  |  | 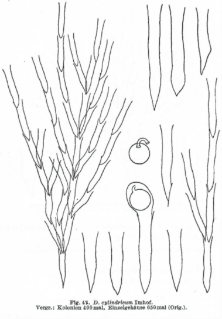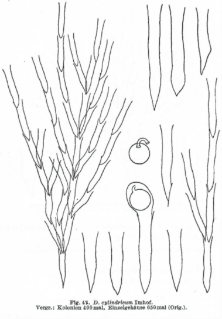 (Fig. 47 in Kreiger, 1930) | | | | | | | | | | | | |
|  |  | ^13^Spherical with spines on surface | 7.5-13.5 in diameter | | | | | Conical collar base with hooked apex. | - | | - | - | - | |
|  |  |  (Figs. 2-7 in Sandergren, 1983) | | | | | | | | | | | | |
|  |  | ^16^Spherical to obovate | 9.5-12.6 in diameter | | | | | Conical collar base with hooked apex | 9.6 | | 2.4-4.5 | - | 41 | |
|  |  | 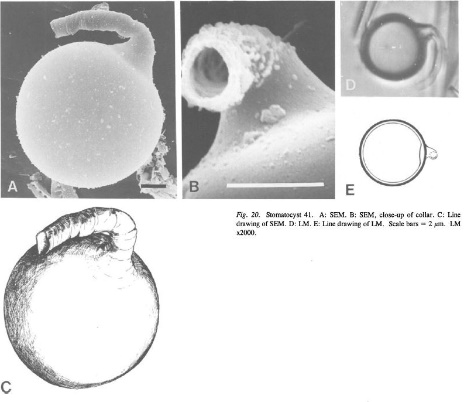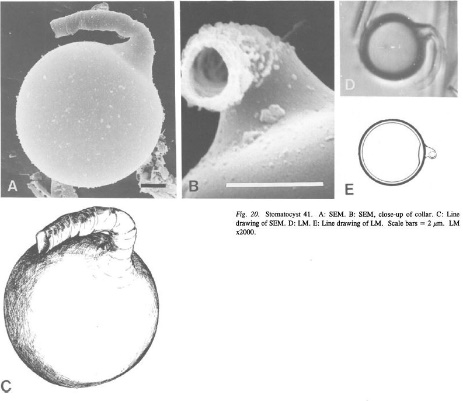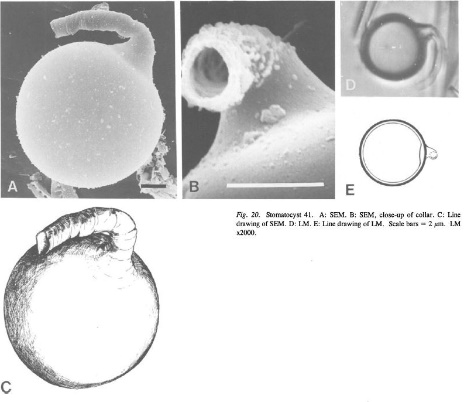 (Figs. 20A-E in Duff et al. 1991) | | | | | | | | | | | | |
| *D. cylindricum* var. *holsaticum*^5^ |  | Spherical |  |  | | | | Cylindrical collar with curved apex | - | | Basal 2.5-3.7  Apical 1.4-2.2 | - | - | |
|  |  | 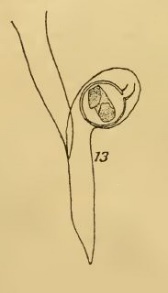 (Fig. 13 in Lemmermann, 1910) | | | | | | | | | | | | |
| *D. cylindricum* var. *palustre*^14, 21^ |  | ^14^Spherical with spines on surface | - | - | | | | Hooked apex (?) | - | | - | - | - | |
|  |  |  (Fig. 25 in Smith & White, 1985) | | | | | | | | | | | | |
|  | Bonghwa040718C | ^21^Spherical with smooth surface | 11.0-13.5 | | 11.8-13.6 | | | Flat planar annulus with conical base and wrinkled apex | 1.4-2.6 | | - | Regular | - | |
|  |  | 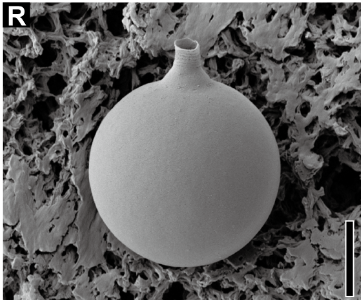 (Fig. 10V in this study. Scale bar = 5 µm) | | | | | | | | | | | | |
| *D. divergens*^3,4,7,,12,16^ |  | ^3,4^Spherical | 14-15 in diameter | | | | | Cylindrical | - | | - | - | - | |
|  |  | 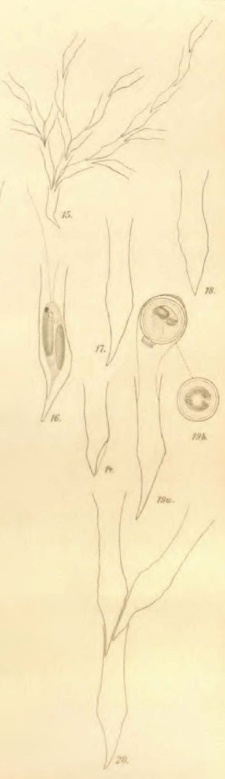 (Table XIX, Figs 19a-b in Lemmermann, 1900) | | | | | | | | | | | | |
|  |  | ^7^Spherical | 11.0-13.0 | | | | | Cylindrical | - | | - | - | - | |
|  |  | 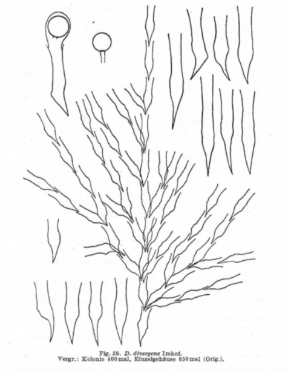 (Fig. 59 in Kreiger, 1930) | | | | | | | | | | | | |
|  |  | ^12^Spherical with smooth surface | 11.5-13 in diameter | | | | | Cylindrical | 1.0-1.3 | | - | - | - | |
|  |  |  (Figs 6-7 in Sheath, 1975) | | | | | | | | | | | | |
|  |  | ^16^Spherical to slightly oval | 9.6-12.7 in diameter | | | | | Cylindrical | 1.0-2.8 | | 2.1-3.1 | - | 161 | |
|  |  | 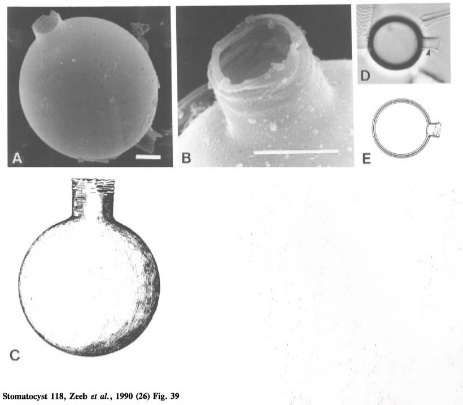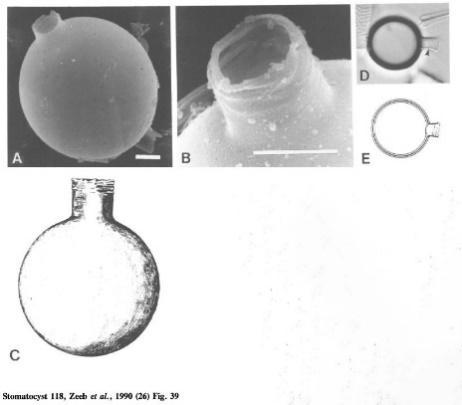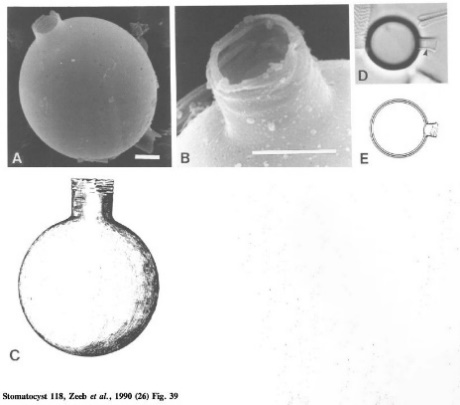(Figs.38A-E in Duff et al. 1991) | | | | | | | | | | | | |
| *D. inclinatum*^21^ | Chojeon011219A | Spherical to oblate with smooth surface | 9.9-13.7 | | 10.6-14.3 | | | Hooked, flat planar annulus with conical base and wrinkled apex | Collar base 1.1-1.9  Hooked apex 1.5-2.7 | | Basal 1.4-3.6  Apical 1.0-2.4 | Regular | - | |
|  |  | 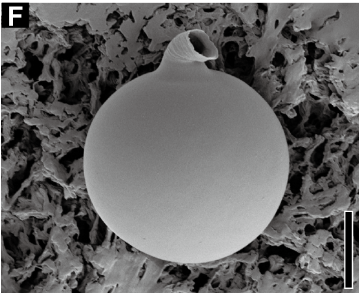 (Fig. 10F in this study. Scale bar = 5 µm) | | | | | | | | | | | | |
| *D. pediforme*^5,6,20^ |  | ^5^Spherical | 11 in diameter | | | | | Absence | - | - | | - | - | |
|  |  | 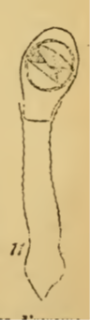 (Fig. 11 in Lemmermann, 1910) | | | | | | | | | | | | |
|  |  | ^6^Spherical | - | | | - | | Absence | - | | - | - | - | |
|  |  | 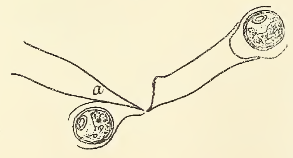 (Fig. 10a in Steinecke, 1915) | | | | | | | | | | | | |
|  |  | ^20^Spherical with smooth surface | 9.0-11.4 in diameter | | | | | Conical, sometimes with flat planar annulus | 0.2-0.5 | | Basal 1.2-2.9  Apical 1.7-2.2 | - | 61 | |
|  |  |  (Figs. 2b, 2d in Piatek et al., 2020) | | | | | | | | | | | | |
| *D. praecambrianum*^17^ |  | Spherical | 9.0-11.0 in diameter | | | | | Cylindrical | 1.0 | | 2.0 | - | - | |
|  |  |  (Fig. 16 in Nicholls, 2000) | | | | | | | | | | | | |
| *D. sertularia*^1,2,4,,8,19^ |  | ^1^Spherical |  | | | | |  | - | | - | - | - | |
|  |  | 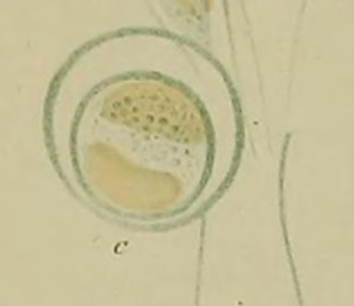 (Fig. 11a-c in Butschlii, 1878) | | | | | | | | | | | | |
|  |  | ^2^Spherical to widely elliptic (?) |  | | | | |  |  | |  |  |  | |
|  |  | 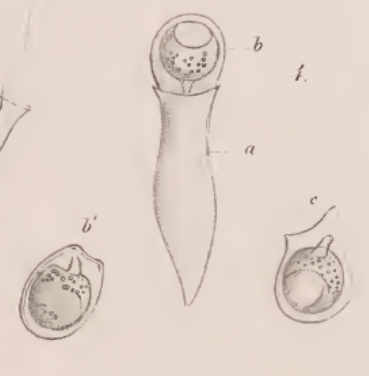 (Fig. 4 in Stein, 1878) | | | | | | | | | | | | |
|  |  | ^4^Spherical | 14-16 in diameter | | | | | Neck-like |  | |  |  |  | |
|  |  | ^8^Spherical | - | | | - | | Hooked | - | | - | - | - | |
|  |  |  (Fig. 2d in Mack, 1951) | | | | | | | | | | | | |
|  |  | ^19^Spherical with smooth surface. | 13.4-16.4 in diameter | | | | | Cylindrical | 1.2-1.6 | | 4.2-4.8 | - | 48 | |
|  |  |  (Figs. 10-11 in Piatek et al., 2012) | | | | | | | | | | | | |
| *D. sertularia* var. *vindobonensis*^7^ |  | Spherical | 12-15 in diameter | | | | | Cylindrical, sometimes slightly curved. | - | | - | - | - | |
|  |  |  (Fig. 2b in Mack, 1951) | | | | | | | | | | | | |
| *D. sociale*^5,7,11^ |  | ^5^Spherical or somewhat elongated | 12.0-14.0 in diameter | | | | | - | - | | - | - | - | |
|  |  | ^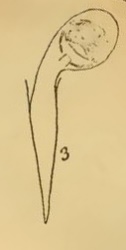^ (Fig. 2 in Lemmermann, 1910) | | | | | | | | | | | | |
|  |  | ^7^Spherical | 12.0-14.0 | | | | | Cylindrical | - | | - | - | - | |
|  |  | 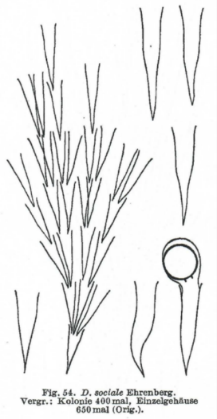(Fig. 54 in Kreiger, 1930) | | | | | | | | | | | | |
|  |  | ^11^Spherical | 12.0 in diameter | | | | | Cylindrical | - | | - | - | - | |
|  |  |  (Fig. 10 in Kristiansen, 1964) | | | | | | | | | | | | |
| *D. sociale* var. *americanum*^16,18^ |  | ^16^Spherical with irregular verrucae, sometimes baculate spines on stomatocyst surface | 7.7-13.1 in diameter | | | | | Obconical | 1.5-4.4 | | 1.9-3.8 | - | 79 | |
|  |  | 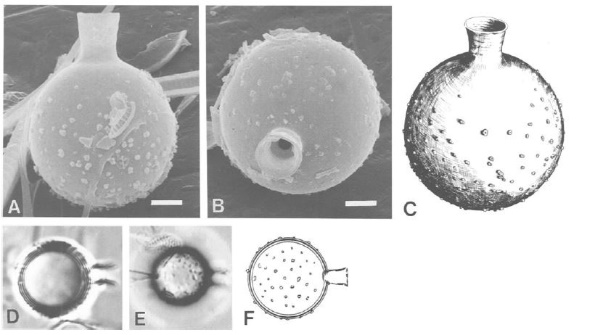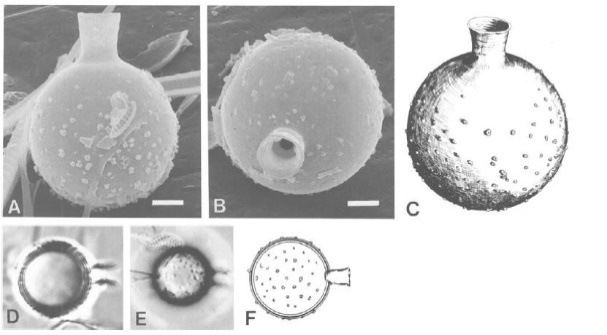(Figs. 58A-F in Duff et al., 1991) | | | | | | | | | | | | |
|  |  | ^18^Spherical with scabrae and verrucae | 10.5-11.3 in diameter | | | | | Obconical | 1.4-2.0 | | 2.5-3.1 | - | 79 | |
|  |  |  (Figs. 14-17 in Piatek & Kowalska, 2008) | | | | | | | | | | | | |
| *D. spinum*^21,22^ | Geumgok020610D | Spherical with echinate spines on cyst body surface | 10.2-12.2 | | 9.6-12.2 | | | Flat planar annulus with conical base and wrinkled apex | 1.2-2.3 | | Basal 2.7-3.4  Apical 1.3-2.0 | Swollen pseudoannulus | - | |
|  |  | 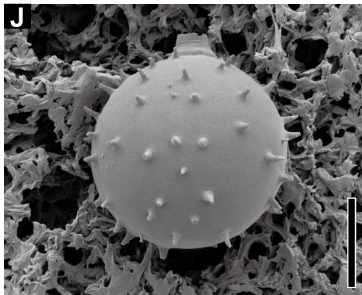 (Fig. 10J in this study. Scale bar = 5 µm) | | | | | | | | | | | | |
|  | (n=25) | Spherical with echinate spines on cyst body surface | 9.8-11.8 | | 10.4-11.7 | | | Flat planar annulus with conical base and elongated apex | 4.0-7.2 | | Basal 2.0-3.0  Apical 1.0-2.7 |  |  | |
|  |  | 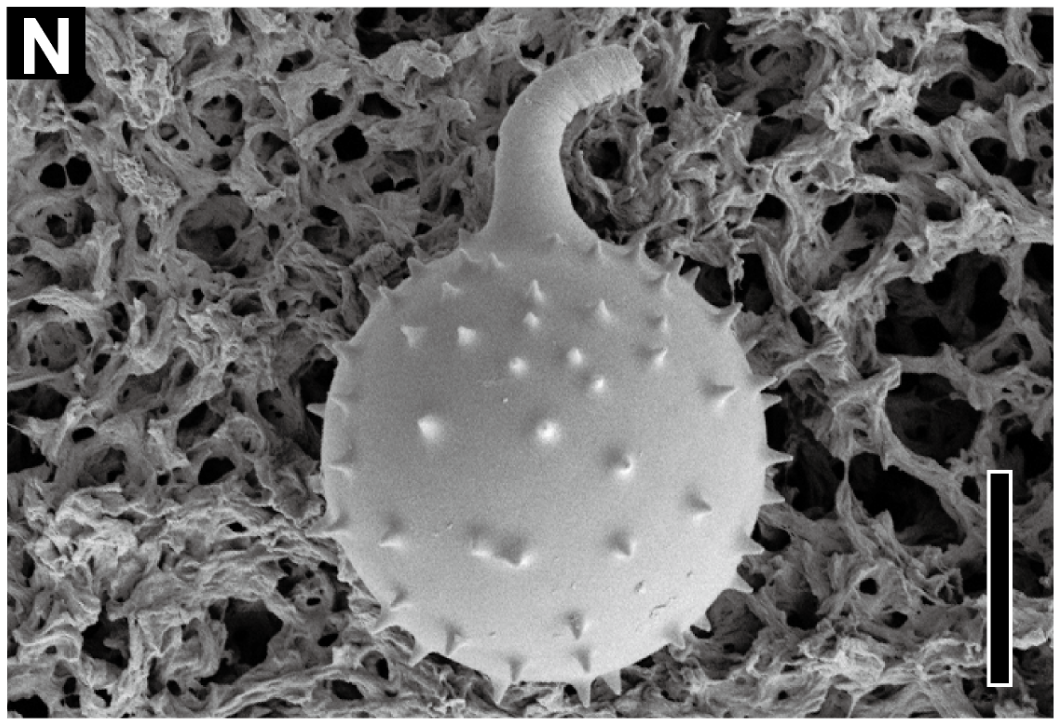 (Fig. 10N in this study. Scale bar = 5 µm) | | | | | | | | | | | | |
| *D. taiyuanensis*^21^ |  | Spherical with smooth surface | 9.3-12.1 | | 9.8-12.6 | | | Slightly curved flat planar annulus | 2.1-5.8 | | 2.6-3.2 | Regular | - | |
|  |  | 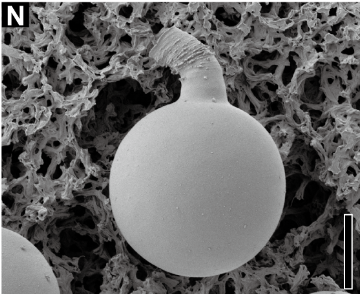 (Fig. 10R in this study. Scale bar = 5 µm) | | | | | | | | | | | | |
| *D. ungeuntariforme*^15^ |  | Spherical with smooth surface | 12 in diameter | | | | | - | - | - | | - | - | |
|  |  |  (Fig. 3 in Croome et al. 1988) | | | | | | | | | | | | |

1. Butschlii, 1878, 2. Stein, 1878, 3. Lemmermann, 1900, 4. Lemmermann, 1904, 5. Lemmermann, 1910, 6. Steinecke, 1915, 7. Kreiger, 1930, 8. Mack, 1951, 9. Asmund, 1955a, 10. Hillard & Asmund, 1963, 11. Kristiansen, 1964, 12. Sheath et al., 1975, 13. Sandgren, 1983a, 14. Smith & White, 1985, 15. Croome et al. 1988, 16. Duff et al., 1995, 17. Nicholls, 2000, 18. Piatek & kowalska, 2008, 19. Piatek et al. 2012, 20. Piatek at al. 2020, 21. Culture sample, 22. Field sample.
