# Supplementary material for: Multigene phylogeny reveals a cryptic diversity in the genus Dinobryon (Chrysophyceae) with integrative description of five new species
Source: Front Plant Sci. 2023 Apr 18;14:1150814. doi: 10.3389/fpls.2023.1150814 (PMC10151809; doi:10.3389/fpls.2023.1150814)
Supplement: Supplementary file 7 [file Table_5.docx]

# Supplementary Material References

Bachmann, H. (1901). Beitrag zur Kenntnis der Schwebeflora der Schweizerseen. *Biol. Zentralbl*. 5, 193-209.

Bachmann, H. (1911). *Das Phytoplankton des Süsswassers mit besonderer Berücksichtigung des Vierwaldstättersees*. Luzern: Buchdruckerei J. Schills Erben.

Bachmann, H. (1921). *Beiträge zur Algenflora des Süsswassers von Westgrönland*. Luzern: Kommissionsverlag Eugen Haag.

Brunnthaler, J. (1901). Die coloniebildenden *Dinobryon* arten (Subgenus Eudinobryon Lauterborn). *Verh. K.K. Zool.-Bot. Ges. Wien*. 293-306.

Chodat, R. (1897). Etudes de biologie lacustre. *Bull. Herb. Boissier* 5, 289-314.

Croome, L. R., Ling, U. H. and Tyler, A. P. (1988). *Dinobryon unguentariforme* (Chrysophyceae), a new species from Australia. *Br. Phycol. J*. 23, 129-133. doi: 10.1080/00071618800650151

Hadziavdic, K., Lekang, K., Lanzen, A., Jonassen, I., Thompson, M. E. and Troedsson, C. (2014). Characterization of the 18S rRNA gne for dsigning uiversal ekaryote secific pimers. *PLoS One* 9(2), e87624. doi: 10.1371/journal.pone.0087624

Hall, D. J. and Karol, G. K. (2016). An inventory of the algae (excluding diatoms) of lakes and ponds of Harriman and Bear Mountain State Parks (Rockland and Orange Counties, New York, U.S.A.). *Brittonia* 68(2), 148-169. doi: 10.1007/s12228-016-9409-5

Jo, B. Y., Shin, W., Boo, S. M., Kim, H. S. and Siver, P. A. (2011). Studies on ultrastructure and three-gene phylogeny of the genus *Mallomonas* (Synurophyceae). *J. Phycol.* 47, 415–425. doi: 10.1111/j.1529-8817.2010.00953.x

Medlin, L., Elwood, J. H., Stickel, S. and Sogin, L. M. (1988). The characterization of enzymatically amplified eukaryotic 16S-like rRNA-coding regions. *Gene* 71(2), 491-499. doi: 10.1016/0378-1119(88)90066-2

Nicholls, H. K. (2000). Three new freshwater species of *Dinobryon* (Chrysophyceae). *Phycologia* 39(2), 134-138. doi: 10.2216/i0031-8884-39-2-134.1

West, W. and West, G. S. (1909). The phytoplankton of the English lake district. *Naturalist* 34, 323-331.
